# Synthesis, Anticancer Evaluation and Structure-Activity Analysis of Novel (*E*)- 5-(2-Arylvinyl)-1,3,4-oxadiazol-2-yl)benzenesulfonamides

**DOI:** 10.3390/ijms21062235

**Published:** 2020-03-23

**Authors:** Krzysztof Szafrański, Jarosław Sławiński, Łukasz Tomorowicz, Anna Kawiak

**Affiliations:** 1 Department of Organic Chemistry, Faculty of Pharmacy, Medical University of Gdańsk, Al. Gen. J. Hallera 107, 80-416 Gdańsk, Poland; jaroslaw.slawinski@gumed.edu.pl (J.S.); lukasz.tomorowicz@gumed.edu.pl (Ł.T.); 2 Department of Biotechnology, Intercollegiate Faculty of Biotechnology, University of Gdańsk and Medical University of Gdańsk, ul. Abrahama 58, 80-307 Gdańsk, Poland; anna.kawiak@biotech.ug.edu.pl

**Keywords:** benzenesulfonamide, synthesis, 1,3,4-oxadiazole, anticancer activity, QSAR, cluster analysis

## Abstract

To learn more about the structure–activity relationships of (*E*)-3-(5-styryl-1,3,4-oxadiazol-2-yl)benzenesulfonamide derivatives, which in our previous research displayed promising in vitro anticancer activity, we have synthesized a group of novel (*E*)-5-[(5-(2-arylvinyl)-1,3,4-oxadiazol-2-yl)]-4-chloro-2-R^1^-benzenesulfonamides **7**–**36** as well as (*E*)-4-[5-styryl1,3,4-oxadiazol-2-yl]benzenesulfonamides **47**–**50** and (*E*)-2-(2,4-dichlorophenyl)-5-(2-arylvinyl)-1,3,4-oxadiazols **51**–**55**. All target derivatives were evaluated for their anticancer activity on HeLa, HCT-116, and MCF-7 human tumor cell lines. The obtained results were analyzed in order to explain the influence of a structure of the 2-aryl-vinyl substituent and benzenesulfonamide scaffold on the anti-tumor activity. Compound **31**, bearing 5-nitrothiophene moiety, exhibited the most potent anticancer activity against the HCT-116, MCF-7, and HeLa cell lines, with IC50 values of 0.5, 4, and 4.5 µM, respectively. Analysis of structure-activity relationship showed significant differences in activity depending on the substituent in position 3 of the benzenesulfonamide ring and indicated as the optimal *meta* position of the sulfonamide moiety relative to the oxadizole ring. In the next stage, chemometric analysis was performed basing on a set of computed molecular descriptors. Hierarchical cluster analysis was used to examine the internal structure of the obtained data and the quantitative structure–activity relationship (QSAR) analysis with multiple linear regression (MLR) method allowed for finding statistically significant models for predicting activity towards all three cancer cell lines.

## 1. Introduction

Malignant diseases are one of the major threats to global public health. Based on the GLOBOCAN 2018 database, which gathered estimates of incidence and mortality for 36 types of cancer in 185 countries, the number of new cases of cancer in 2018 was estimated to be 18.1 million and the number of deaths from cancer was 9.6 million worldwide [1]. In 2009, the medical and indirect costs of new cancer cases globally were estimated to be 286 billion $ [2]. Despite advanced work on new methods of cancer therapy such as immunotherapy, nanoscale and nanostructure-based therapeutics or gene therapy, the main methods of choice remain surgery, radiotherapy and chemotherapy [3]. Therefore, the search for novel small molecule chemotherapeutic drugs is one of the major challenges of modern medicinal chemistry.

**Figure 1:**
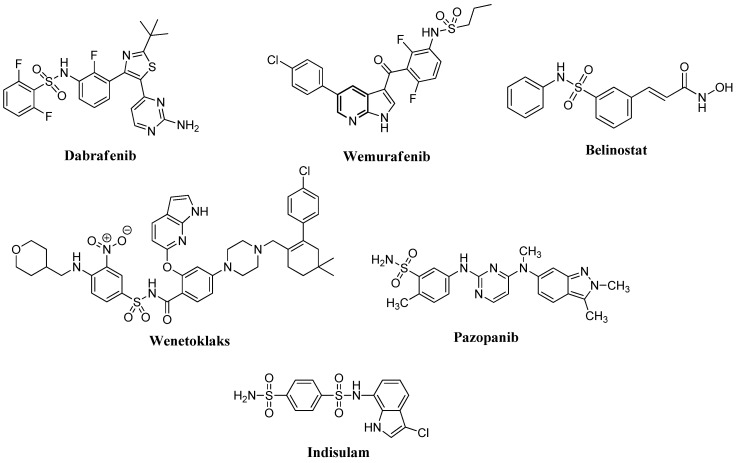
Sulfonamides with proven anticancer activity.

Sulfonamides as a class of biological active compounds have been widely used since the 1940s, mainly as antibacterial, hypoglycemical, diuretics and antiglucoma drugs. Furthermore, sulfonamide moiety is an important part of the molecular structure of drugs for treatment of inflamation (Celecoxib), viral infections (Simeprevir, Amprenavir), erectile dysfunction (Sildenafil), and arrhythmia (Dofetilide). Recently, several sulfonamide derivatives have been approved by the FDA for use in the therapy of cancer; moreover, there are numerous reports of anticancer sulfonamides in the preclinical tests [4,5,6,7,8]. Derivatives containing a sulfonamide moiety so far have demonstrated several diverse molecular mechanisms of anticancer action. Some of the most important of them are: inhibition of vascular endothelial growth factor receptor (VEGFR) (Pazopanib) [9], inhibition of prosurvival BCL-2 family proteins (Wenetoklaks) [10], inhibition of histone deacetylases (Belinostat) [11], selective and specific inhibition of mutated serine/threonine-protein kinase BRAF V600 (Vemurafenib and Dabrafenib) [12] and the disturbing of the splicing process (Indisulam) [13] (Figure 1).

Our efforts for a long time have been focused on extensive research on the antineoplastic activity of 2-mercaptobenzenensulfonamide derivatives [14,15,16,17]. Recently, we synthesized a large group of 2-(benzylthio)-4-chloro-5-[(5-(substituted)-1,3,4-oxadiazol-2-yl)]benzenesulfonamides, and found that among them a series of (*E*)-2-(benzylthio)-4-chloro-5-(5-styryl-1,3,4-oxadiazol-2-yl)benzenesulfonamides display significant antitumor activity [18].

**Figure 2:**
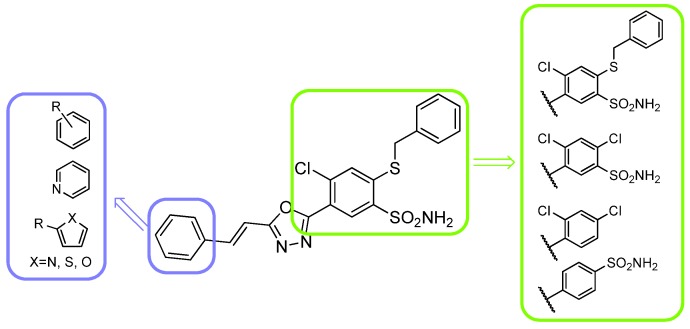
Hit compound and modification directions of the aryl ring (blue frame) and the sulfonamide scaffold (green frame).

Based on obtained information, we decided to optimize the “hit” structure to better understand the structure–activity relationships in this group of promising anticancer compounds. Therefore, firstly we decided to test the effect of the change in the aryl ring in the vinyl position (Ar) by synthesis derivatives differentiated with substituted benzene ring and 5- or 6-membered heterocycles in that position. Secondly, we checked the significance of the benzylthio substituent at position 2 of the benzenesulfonamide scaffold by comparing compounds containing benzylthio substituent or a chlorine atom. Finally, we decided to check the significance of the sulfonamide moiety in the *meta* position relative to the oxadiazole ring by synthesizing analogous compounds based on the 4-benzenesulfonamide and 2,4-dichlorobenzene scaffold (Figure 2).

## 2. Results and Discussion

### 2.1. Chemistry

**Scheme 1:**
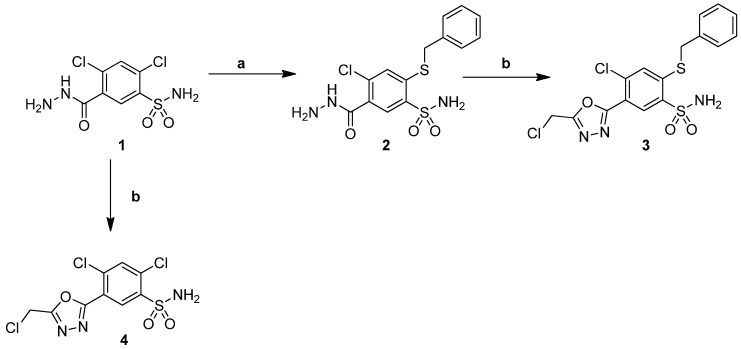
Synthesis of compounds **2-4**. *Reagents and conditions:* (**a**) K_2_CO_3_ (2.1 equiv.), TBAI (0.01 equiv.), phenylmethanethiol (1.0 equiv.), MeCN/H_2_O, r.t., 24 h; (**b**) 2-chloro-1,1,1-trimethoxyethane (1.5 equiv.), 1,4-dioxane, reflux, 18 h.

Recently, we described how the treatment of 2,4-dichloro-5-(carbazoyl)benzenesulfonamide **1** with phenylmethanethiol in the presence of tetrabutylammonium iodide (TBAI) as a phase transfer catalyst resulted in the selective substitution of chlorine atom in position 2 of the benzene ring, yielding 2-benzylthio-4-chloro-5-(carbazoyl)benzenesulfonamide **2** [18]. Then, reacting hydrazide **1** or **2** with triethyl orthochloroacetate led to a cyclization product, i.e., corresponding 2-(chloromethyl)-1,3,4-oxadiazole derivatives **4** or **3** (Scheme 1).

**Scheme 2:**
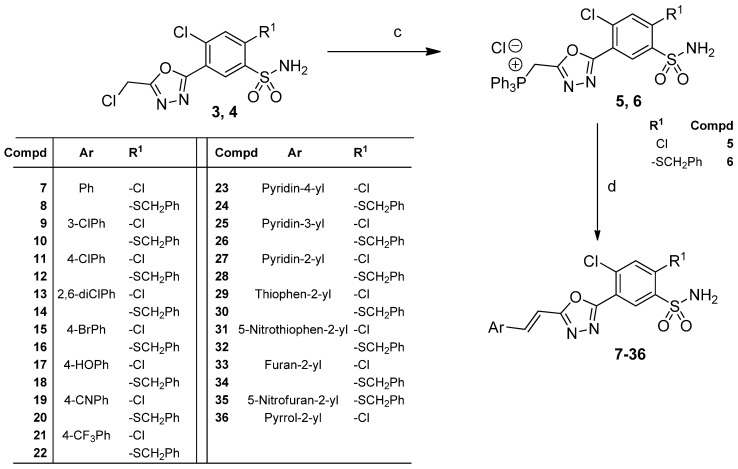
Synthesis of compounds **5**–**38**. *Reagents and conditions:* (**c**) PPh_3_ (1.15 equiv.), MeCN, reflux 18 h; (**d**) aromatic aldehyde (1.2 equiv.), DIPEA (2 equiv.), CH_2_Cl_2_, r.t. 24 h.

To obtain a series of (*E*)-5-[5-(2-arylvinyl)-1,3,4-oxadiazol-2-yl]benzenesulfonamides **7-36** in Wittig reaction, compounds **3** and **4** were first transformed into triphenylphosphonium chlorides **5** and **6** by refluxing with triphenylphosphine in dry acetonitrile. Further Wittig reactions of **5** and **6** with various arylaldehydes in dichloromethane with the presence of *N*,*N*-diisopropylethylamine under inert atmosphere afforded the desired (*E*)-5-[(5-(2-arylvinyl)-1,3,4-oxadiazol-2-yl)]-4-chloro-2-R^1^-benzenesulfonamides **7**–**36** (Scheme 2).

**Scheme 3:**
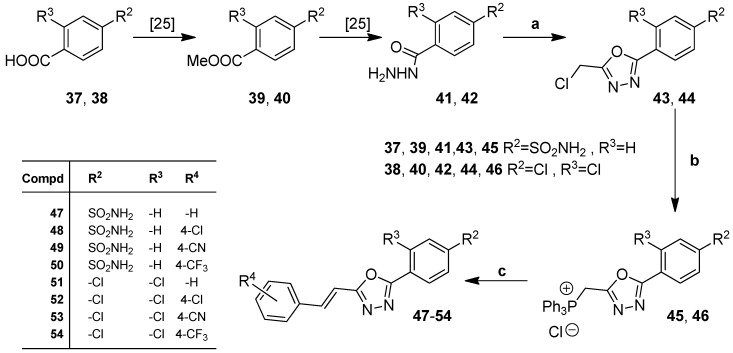
Synthesis of compounds **39-54**. *Reagents and conditions:* (**a**) 2-chloro-1,1,1-trimethoxyethane (1.5 equiv.), 1,4-dioxane, reflux, 18 h; (**b**) PPh_3_ (1.15 equiv.), MeCN, reflux 18 h; (**c**) aromatic aldehyde (1.2 equiv.), DIPEA (2 equiv.), CH_2_Cl_2_, r.t. 24 h.

In turn, a series of either (*E*)-4-[5-(2arylvinyl)-1,3,4-oxadiazol-2-yl)benzenesulfonamides **47**–**50** or 2-(2,4-dichlorophenyl)-5-(2-arylvinyl)-1,3,4-oxadiazoles **51**–**54** were prepared according to the reaction sequence shown in Scheme 3.

Thus, first we obtained methyl esters **39**, **40,** which were transformed into hydrazides **41** and **42**. Next, analogously to previous derivatives (**1**–**36**), hydrazides **41** and **42** were submitted to cyclization with 2-chloro-1,1,1-trimethoxyethane. Obtained 2-(chloromethyl)-5-phenyl-1,3,4-oxadiazoles **43** and **44** were used to produce triphenylphosphonium chlorides **45** and **46,** which in Wittig reaction with R^4^-benzaldehydes were converted to final compounds **47**–**54**.

Although standard crystallization of crude product from glacial acetic acid in general yielded in pure *E* isomer, for several compounds we obtained an *E*/*Z* mixture in proportion 1/0.2-1/1 (according to ^1^H NMR integration and HPLC analysis). Thus, taking effort to obtain a pure isomer, we observed that refluxing *E*/*Z* mixture in acetic acid for 24–48 h results in isomerization to pure *E* isomer. Isomer assignment was based on the value of ^1^H NMR coupling constant between vinyl protons. According to Karplus relation for alkenyl vicinal protons, coupling constant is about 6-12 Hz for *Z* (*cis*) and 12-18 Hz for *E* (*trans*) isomer. For target derivatives **7**–**36** and **47**–**54,** we observed coupling constants about 16 Hz for pure *E* isomer and in range 12-13 Hz for *Z* isomer (obtained only in inseparable *E/Z* mixture). The structures of all compounds were confirmed with spectroscopic methods (IR, ^1^H NMR, ^13^C NMR) and elemental analyses, as shown in the Materials and Methods section.

### 2.2. Anticancer Activity

Compounds **7**–**36** and **47**–**54** were tested regarding their effect on growth of three human cancer cell lines: colon cancer HCT-116, breast cancer MCF-7 and cervical cancer HeLa, as well as on noncancerous keratinocyte cell line HaCaT. Cell viability was measured with MTT assay after 72 h of incubation with tested compound in five concentration 1–100 μM. The MTT test enables us to quantify the living cells by measuring the activity of mitochondrial enzymes, enables reducing tetrazolium dye MTT to purple formazan. In this manner, IC_50_—molar concentration [µM] that inhibits 50% net cell growth—was determined (Table 1 and Table 2).

### 2.3. Structure-Activity Relationship 

Based on the results of anticancer screening presented in Table 1, several important conclusions on the structure-activity relationship of compounds **7**–**36** can be made:

First of all, it can be easily seen that the human colon cancer cell line HCT-116 is the most susceptible to the tested compounds since 50% of them exhibit IC_50_ ≤ 25µM.

Among all tested compounds, **31** (Ar = 5-nitrothiphene R^1^=Cl) exhibits outstanding cytotoxic activity with 0.5, 4, and 4.5 µM IC_50_ values for HCT-116, MCF-7, and HeLa, respectively.

When comparing compounds **29** and **31**, it can be seen that an addition of a nitro group to thiophene ring causes increasing of activity towards HCT-116 over 200 times, towards MCF-7 43 times and towards HeLa 54 times. In contrast, for benzylthio derivatives **30** and **32**, the same modification of Ar substituent does not cause a significant change in activity. On the other hand, a high increase in activity and selectivity for HCT-116 is observed when replacing 5-nitrothiophen of **32** to 5-nitrofuran of **35**, unfortunately we did not manage to obtain similar 5-nitrofuran derivatives with R^1^ = Cl.

Among derivatives with 2-benzylthio substituent in position 2 of benzenesulfonamide, the highest overall activity in the range 9–12 µM against all three cancer lines is exhibited by compound **20** (Ar = 4-CNPh), while compound **35** exhibits very high activity selectively to HCT-116 (IC_50_ = 4 µM).

The introduction of the pyridine ring as an Ar substituent (**23**–**28**) significantly weakens activity; however, low activity of 25-85 µM against HCT-116, for compounds with the R^1^ = SBn substituent (**24**, **26**, **28**), is still observed.

The introduction of halogen atoms in the benzene ring of the Ar substituent, for compounds **9**, **11**, **13**, **15**, for which R^1^ = Cl, has a beneficial effect on activity against all three lines compared to compound **7**. However, the activity of these compounds is still very varied and is in a wide range from 16 to 150 µM for all three cancer lines. On the other hand, for compounds **10**, **12**, **14** and **16**, for which R^1^ = SBn, the introduction of halogen atoms does not significantly increase activity relative to the unsubstituted Ar ring of compound **8**. As a consequence, the activity of these compounds is in a narrow range, namely 11–40 µM.

Analyzing the influence of R^1^ substituent, it can be clearly seen that compounds with benzylthio substituent (R^1^ = SBn) (**8**–**34** even number, and **35**) almost always show higher activity to each cell line than their counterparts with a chlorine atom in the R^1^ position (R^1^ = Cl) (**7**–**33** odd number, and **36**). Consequently, compounds with R^1^ = SBn globally show lower average of activity than R^1^ = Cl derivatives (Table 3). On the other hand, the main feature of compounds with the R^1^ = Cl substituent is that the small change in the Ar ring causes a much more significant change in activity than for R^1^ = SBn analogs. This property of a large variety of activities of R^1^ = Cl group is visible in the wider range of IC_50_ values and higher standard deviation (Table 3). Furthermore, each compound with R^1^ = Cl shows greater differentiation in activity between the tested cell lines.

Considering the selectivity of the tested compounds, we can see that the most selective is compound **35**, which is 22 times more active against HCT-116 than HaCaT, 37 times more than MCF-7 and 17 times more than HeLa. In general, when R^1^ = Cl, selectivity index SI (which represents IC_50_ for HaCaT cell line/IC_50_ for cancerous cell line) for active derivatives (for which IC_50_ for cancer line ≤ 25 µM) is in the range from 2 to 16, depending on the cell line. Meanwhile, if R^1^ is SBn, it reaches 13 (**14**) and 22 (**35**); however, in this group there are also several cases (**8**, **10**, **18**) where activity against HaCaT is at the same level as against cancer lines.

**Figure 3:**
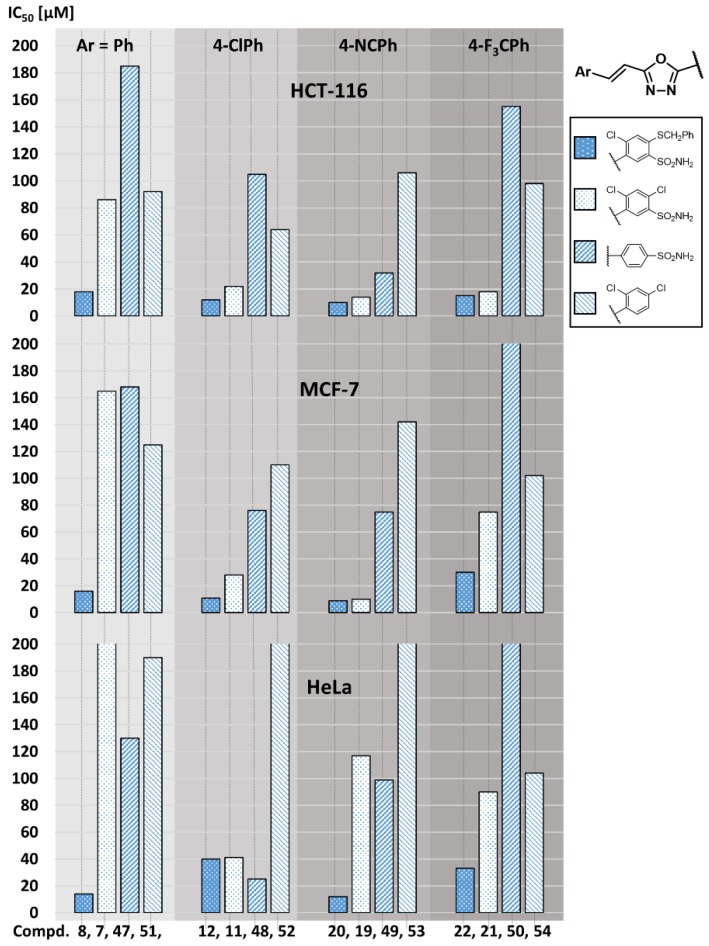
Comparison of cytostatic activity of compounds with the same Ar substituent but differing in main scaffold: 2-(benzylthio)-4-chlorobenzenesulfonamide (**8**, **12**, **20**, **22**), 2,4-dichlorobenzenesulfonamide (**7**, **11**, **19**, **21**) 4-benzenesulfonamide (**47**–**50**) and 2,4-dichlorobenzene (**51**–**54**).

In the next step, to investigate the significance of the sulfonamide moiety we compared (Figure 3) compounds with the same pattern of vinyl substitution (Ph, 4-ClPh, 4-NCPh or 4-F_3_CPh) but built on the different scaffolds: 2-(benzylthio)-4-chloro-5-(1,3,4-oxadiazol-2-yl)benzenesulfonamide (**8**, **12 20** and **22**), 2,4-dichloro-5-(1,3,4-oxadiazol-2-yl)benzenesulfonamide (**7**, **11**, **19** and **21**) 4-(1,3,4-oxadiazol-2-yl)benzenesulfonamide (**47**–**50**) or 2-(2,4-dichlorophenyl)-1,3,4-oxadiazole (**51**–**54**). 

Similarly, as previously established, compounds build on 2-(benzylthio)-4-chlorobenzenesulfonamide scaffold proved to be substantially more active than others. 

Comparing compounds built on 2,4-dichlorobenzene (**51**–**54**) and 2,4-dichlorobenzenesulfonamide scaffold (**7**, **11**, **19** and **21**), it can be seen that the presence of sulfonamide moiety always increases the activity against HCT-116, regardless of Ar substituent, while the MCF-7 and HeLa cell lines compounds without sulfonamide display higher, but generally weak, activity only when Ar = Ph (comp **51** compering to **7**). When the phenyl ring is substituted with 4-Cl, 4-CN or 4-CF_3_, the compounds with sulfonamide moiety are always more active (**11**, **19** and **21**).

On the other hand, compounds possessing sulfonamide moiety in the *para* position to oxadiazole ring (**47**–**50**) are less active towards MCF-7 and HCT-116 than compounds with a sulfonamide in the *meta* position, both with SBn or Cl R^1^substituent (**7**, **8 11**, **12**, **19**–**22**). Some exceptions are observed only for HeLa cell lines, especially for compound **48**, which, for this cell line, displays the lowest IC_50_ value of 25 µM.

### 2.4. Quantitative Structure-Activity Relationship Analysis

In order to explain the reasons for the observed variability in the anticancer activity of compounds **7**–**36**, determine the most important parameters controlling pharmacological effects and obtain QSAR equations that will allow us to predict activity of a future compound, we conducted a statistical analysis basing on a set of computed quantitative molecular descriptors. As the values of many descriptors strictly depend on the three-dimensional structure, we performed a two-step procedure to determine the lowest-energy conformation. First, it consists of searching the conformational space of compounds using molecular mechanics. Then, the lowest energy conformations were treated as input conformations for semi-empirical geometry optimization using the PM6 method, which ensures an optimal ratio of precision to computation time. Compounds with optimal conformation were sent to the Molecular Operating Environment software (Chemical Computing Group) to calculate a set of 293 quantitative molecular descriptors.

**Figure 4:**
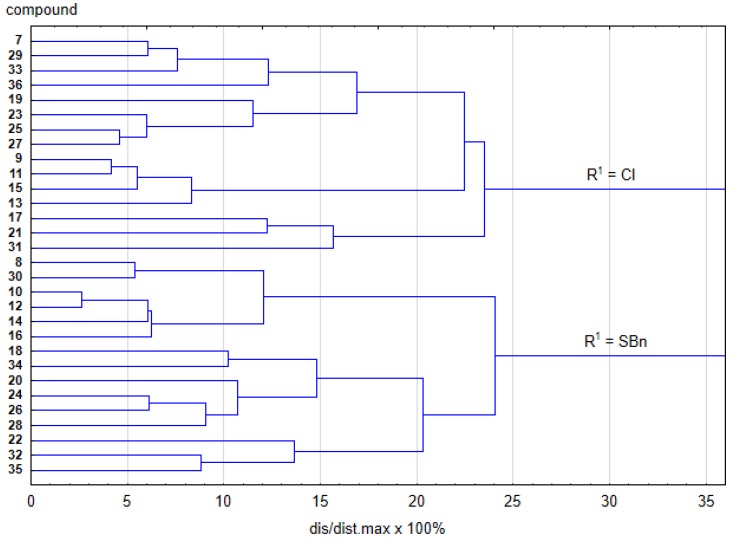
Dendrogram of hierarchical cluster analysis performed with Ward’s method for compounds **7**–**36** in view of their molecular descriptors.

To check the internal structure of the obtained data, we used hierarchical cluster analysis to group compounds **7**–**36** based on the received descriptors. Cluster analysis (CA) was used for the natural grouping of samples in clusters that are not known beforehand, with a common property characterized by the values of a set of variables [19]. In QSAR modelling, it is widely used to check out the homogeneity of data, identify some unusual data points, detect patterns, and represent potentially interesting relationships in the data [20,21]. The results are presented in the form of a dendrogram (Figure 4) which displays the distance level at which there was a combination of objects and clusters—the more on left the clusters merge, the more dissimilarity they show.

The most visible and unambiguous observation is the division into compounds with the substituent R^1^ = Cl and R^1^ = SBn, which are grouped at the end of the dendrite. This indicates a large difference between these two groups in terms of molecular properties, which, along with the differences shown in Table 3, gives us the basis to treat these two groups separately when looking for QSAR equations.

Analyzing the dendrite, we also see that the most active compound **31** (Ar = 5-nitrothiphene) shows significant dissimilarity from other compounds clustering as the latest single compound with **17** (Ar = 4-HOPh) and **21** (Ar = 4-NCPh). This leads us to the conclusion that in terms of molecular parameters, it exhibits features of outliers, which may explain the exceptionally high activity of this compound. 

Surprisingly, CA analysis indicates a substantial influence on molecular properties of the substitution pattern of the Ar moiety in the R^1^ = Cl group of compounds. Thus, we can see that compound **7** (Ar = Ph) is more similar to compounds with unsubstituted heterocyclic Ar ring **29** and **33** and next to pyridine derivatives **23**, **25** and **27** rather than to halogen derivatives **9**, **11**, **15** and **13**. Similar relationships have been observed in the anti-cancer activity of these compounds.

In the next step of analysis, a search for QSAR models was performed with the multiple linear regression (MLR) technique along with stepwise algorithm. As a result, we obtained six statistically significant equations (**E1**–**E6**) describing the activity, expressed as pIC_50_ = −logIC_50_[M] (to get its normal distribution), separately for group R^1^ = Cl and group R^2^ = SBn (Table 4).

**Figure 5:**
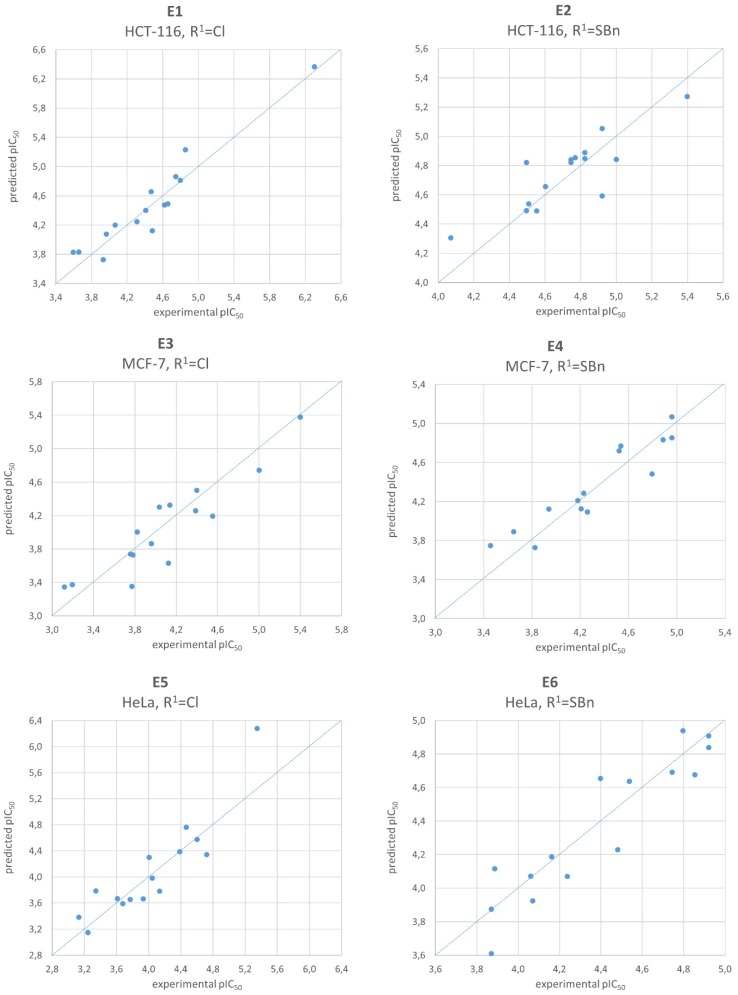
Plots of experimental vs predicted in 5-fold cross validation values of IC_50_ for equations **E1**–**E6**.

The QSAR model must be validated to be considered predictive [22,23]. Due to the small number of observations resulting from dividing the initial set of compounds **7**–**36** into two groups, we decided to apply five-fold-cross-validation (Figure 5) in which each set of 15 compounds is randomly divided into five three-member subsets and in turn each of them is treated as a test set, and the other as a training set. The squared correlation coefficient (Q^2^) obtained in this way are in the range 0.72–0.91 and indicate that our models can be used to confidently predict the activity of newly synthesized compounds.

Besides activity prediction, the QSAR equations indicate which properties of the molecule are favorable for obtaining high activity. In Equations **E2**, **E4** and **E5**, descriptors with the highest influence on activity—with highest regression coefficients—refer to the negatively charged surface of the molecule. These are the fractional charge-weighted negative surface area (FCASA)(**E2**), fractional negative van der Waals surface area (PEOE_VSA_FNEG)(**E4**) and the fractional negative water accessible surface area (FASA)(**E5**). Similarly, in Equations **E1** and **E3**, the descriptors describing the size of the molecule’s surface are the most important for activity: SMR_VSA2 = sum of van der Waals surface of atoms with contribution to Molar Refractivity in the range 0.26–0.35 (**E1**) and vsurf_Wp2 = Polar volume at −0.5 (**E3**). Only activity towards HeLa cell line in R^1^ = SBn group (Equation **E6**) shows a deviation from this regularity, as it depends mostly on Kier and Hall connectivity indices descriptor chi1v_C = carbon valence connectivity index.

## 3. Materials and Methods

### 3.1. Synthesis

The following instruments and parameters were used: melting points were recorded on the Boetius HMK apparatus; (Franz Kustner Nacht KG, Dresden, Germany) and were uncorrected, IR spectra were measured on Thermo Mattson Satellite FTIR spectrometer (Thermo Fisher Scientific, Waltham, MA, USA) in KBr pellets, and the absorption range was 400–4000 cm^−1^. ^1^H NMR and ^13^C NMR spectra were recorded on a Varian Unity Plus 500 apparatus (Varian, Palo Alto, CA, USA), chemical shifts are expressed at δ values relative to Me_4_Si (TMS) as an internal standard.

Purity of compounds was analyzed by RP-HPLC on Shimadzu (Model LC-10AD) HPLC system (Shimadzu Corporation, Kyoto, Japan); Column: Gemini 4.6 × 250 mm; C6-phenyl; 5 µm; 110 Å, Mobile Phase: A-grade water with 0.1% (v/v) trifluoroacetic acid, B - 80% acetonitrile-water containing 0.08% (v/v) trifluoroacetic acid, linear gradient 5–100% B in 60 min, flow rate: 1 mL/min.

Elemental analyses were performed on PerkinElmer 2400 Series II CHN Elemental Analyzer (PerkinElmer, Inc. Waltham, MA USA).

The 2,4-dichloro-5-(carbazoyl)benzenesulfonamide **1** was obtained according to the method described previously [24]. 

Synthesis and spectral data for compounds **2**, **3**, **6**, **8**, **10**, **12, 14** and **16** were reported in our previous work [18].

Methyl esters **39**, **40** hydrazides **41**, **42** were prepared according to methods described in [25] and their melting points and IR spectra were in accordance with generally available literature data. 

2-(Chloromethyl)-5-(2,4-dichlorophenyl)-1,3,4-oxadiazole **44** was prepared by the same method as **3**, **4** and **43** and its melting point and spectra were in accordance with the literature [26].

#### 3.1.1. Procedure for the Preparation of 2,4-Dichloro-5-(5-(Chloromethyl)-1,3,4-Oxadiazol-2-yl])Benzenesulfonamide (**4**)

A mixture of 2,4-dichloro-5-(carbazoyl)benzenesulfonamide (**1**) (2.84 g, 10 mmol) and 2-chloro-1,1,1-trimethoxyethane (2.31 g, 15 mmol) in a mixture of 1,4-dioxane (30 mL) and glacial acetic acid (7.5 mL) was refluxed for 4 h. Next, the solvent was evaporated under reduced pressure, ethanol (5 mL) was added and the suspension was stirred vigorously at room temperature for 1 h. The precipitate was filtered off, washed with cold ethanol and dried, and for analytical purposes crystallization from ethyl acetate was performed. Yield 2.68 g (78%) as a white powder; mp 192–194 °C; (KBr) ν_max_: 3386, 3204 (NH); 3083, 3029 (C_Ar_-H); 2974, 2925 (C-H), 1593, 1575, 1456 (C=C, C=N); 1359, 1176 (SO_2_) cm^−1^; ^1^H NMR (DMSO-*d*_6_, 500 MHz) δ: 5.19 (s, 2H, CH_2_), 7.96 (s, 2 H, NH_2_), 8.20 (s, 1 H, H-3) 8.52 (s, 1 H, H-6)ppm; Anal. C 31.55, H 1.77, N 12.27% calcd for C_9_H_6_Cl_3_N_3_O_3_S (342.59), C 31.54, H 1.67, N 11.87%.

#### 3.1.2. Procedure for the Preparation of ((5-(2,4-Dichloro-5-Sulfamoylphenyl)-1,3,4-Oxadiazol-2-yl)Methyl)Triphenylphosphonium chloride (**5**)

A mixture of compound **4** (2.05 g, 6 mmol) and triphenylphosphine (1.89 g, 7.2 mmol) in dry acetonitrile (60 mL) was refluxed for 24 h. After cooling to room temperature, the precipitated solid was filtered off, and washed with acetonitrile and diethyl ether. 

Yield 3.31 g (93%) as a white powder mp 130°C decomp.; IR (KBr) ν_max_ 3084, 3050, 3017 (C_Ar_-H), 2946, 2898 (C-H), 1589, 1559, 1459 (C=C, C=N), 1341, 1151 (SO_2_) cm^−1^; ^1^H NMR (DMSO-*d_6_*, 500 MHz) δ 6.10 (d, 2H, P^+^CH_2_), 7.77–7.81 (m, 6H, Ar), 7.86 – 7.94 (m, 9H, Ar) 7.97 (s, 2H, NH_2_), 8.13 (s, 1H, H-3) 8.34 (s, 1H, H-6) ppm (Supplementary Materials: Spectrum 1.). Anal. C 53.57, H 3.61, N 7.56% calcd for C_27_H_21_Cl_3_N_3_O_3_PS (604.87), C 53.61, H 3.50, N 6.95%

#### 3.1.3. General Procedure for the Preparation of (*E*)-5-(5-(2-Arylvinyl)-1,3,4-Oxadiazol-2-yl) -4-Chloro-2-R^1^-Benzenesulfonamides (**7**–**36**) 

Triphenylphosphonium chloride **5** or **6** (0.5 mmol) and appropriate aldehyde (0.6 mmol) in 15 mL of dry dichloromethane was put under nitrogen atmosphere and solution of *N*,*N*-diisopropylethylamine (0.13 g, 1mmol) in 5 mL of dichloromethane was added. The reaction mixture was stirred at room temperature for 24 h. Next, the solvent was evaporated under reduced pressure, ethanol (4 mL) was added and the suspension was stirred vigorously at room temperature for 1 h. The precipitate was filtered off and crude product was purified as follows:

(*E*)-2,4-Dichloro-5-(5-styryl-1,3,4-oxadiazol-2-yl)benzenesulfonamide (**7**). 

Using benzaldehyde (0.06 g) and crystalizing the crude product from glacial acetic acid, the title compound **7** was obtained (0.11 g, 55%) as a white powder: mp 263–268 °C; IR (KBr) ν_max_: 3369, 3264 (NH); 3096, 3052, 3022 (C_Ar_-H); 1639, 1560, 1527 (C=C, C=N); 1346, 1162 (SO_2_) cm^−1^; ^1^H NMR (DMSO-*d*_6_, 500 MHz) δ: 7.44–7.48 (m, 4H, C=CH, H-4Ph, H-3,5Ph), 7.71 (d, *J*=16.1 Hz, 1H, CH=C), 7.82 (d, *J*=6.8Hz, 2 H, H-2,6Ph), 7.97 (s, 2H, NH_2_), 8.17 (s, 1 H, H-3), 8.62 (s, 1 H, H-6) ppm(Supplementary Materials: Spectrum 2.); ^13^C NMR (DMSO-*d_6_*, 125 MHz) δ: 110.15, 122.05, 128.51, 129.44, 130.66, 131.07, 134.33, 134.50, 134.94, 136.06, 140.27, 140.99, 160.68, 165.10 ppm (Supplementary Materials: Spectrum 3.).

Anal. C 48.50, H 2.80, N 10.60% calcd for C_16_H_11_Cl_2_N_3_O_3_S (396.24), C 48.29, H 2.82, N 10.44%

(*E*)-2,4-Dichloro-5-(5-(3-chlorostyryl)-1,3,4-oxadiazol-2-yl)benzenesulfonamide (**9**). 

Using 3-chlorobenzaldehyde (0.08 g) and crystalizing the crude product from glacial acetic acid, the title compound **9** was obtained (0.13 g, 61%) as white powder: mp 247-250°C; IR (KBr) ν_max_: 3374, 3270 (NH); 3098, 3055, 3019 (C_Ar_-H); 1590, 1547, 1526 (C=C, C=N); 1343, 1159 (SO_2_) cm^-1^; ^1^H NMR (DMSO-*d*_6_, 500 MHz) δ: 7.47–7.48 (m, 2H, Ph), 7.58 (d, *J*=16.5 Hz, 1H, CH=C), 7.71 (d, *J*=16.5 Hz, 1H, CH=C), 7.80 (t, 1H, H-5Ph), 7.97–7.98 (m, 3H, NH_2_, Ph), 8.18 (s, 1H, H-3), 8.62 (s, 1H, H-6) ppm; Anal. C 44.62, H 2.34, N 9.79 % calcd for C_16_H_10_Cl_3_N_3_O_3_S (430.68), C 44.25, H 2.32, N 9.41% 

(*E*)-2,4-Dichloro-5-(5-(4-chlorostyryl)-1,3,4-oxadiazol-2-yl)benzenesulfonamide (**11**). 

Using 4-chlorobenzaldehyde (0.08 g), the crude product was dissolved in 30 mL glacial acetic acid and refluxed for 36 h; next, the solution was concentrated under reduced pressure to about 3 mL and was left to cool to room temperature, the precipitate formed was filtered off, and the title compound **11** was obtained (0.13 g, 55%) as colorless plates: mp 243–245 °C; IR (KBr) ν_max_: 3313, 3199 (NH); 3092 (C_Ar_-H); 1632, 1589, 1526 (C=C, C=N); 1356, 1172 (SO_2_) cm^−1^; ^1^H NMR (DMSO-*d*_6_, 500 MHz) δ: 7.48 - 7.52 (m, 3 H, C=CH, H-3,5Ph), 7.70 (d, *J*=16.6 Hz, 1 H, CH=C), 7.86 (d, *J*=8.3Hz, 2 H, H-2,6Ph), 7.97 (s, 2 H, NH_2_), 8.17 (s, 1 H, H-3) 8.62 (s, 1 H, H-6) ppm (Supplementary Materials: Spectrum 4.); ^13^C NMR (DMSO-*d_6_*, 125 MHz) δ: 113.14, 124.19, 131.64, 132.40, 133.27, 136.11, 136.52, 136.73, 137.32, 138.27, 141.04, 143.16, 162.96, 167.15 ppm (Supplementary Materials: Spectrum 5.). Anal. C 44.62, H 2.34, N 9.76% calcd for C_16_H_11_Cl_2_N_3_O_3_S (430.69), C 44.28, H 2.30, N 9.65%

(*E*)-2,4-Dichloro-5-(5-(2,6-dichlorostyryl)-1,3,4-oxadiazol-2-yl)benzenesulfonamide (**13**). 

Using 2,6-dichlorobenzaldehyde (0.11 g) and crystalizing the crude product from glacial acetic acid, the title compound **13** was obtained (0.11 g, 45%) as an off white solid: mp 225-228; IR (KBr) ν_max_: 3390, 3296, 3174 (NH); 3089 (C_Ar_-H); 1586, 1534 (C=C, C=N); 1362, 1175 (SO_2_) cm^-1^; ^1^H NMR (DMSO-*d*_6_, 500 MHz) δ: 7.37 (d, *J*=16.5 Hz, 1H, C=CH), 7.44 (t, 1 H, H-4Ph) 7.61 (d, 2H, H-3,5Ph), 7.73 (d, *J*=16.5 Hz, 1H, CH=C), 7.97 (s, 2 H, NH_2_), 8.18 (s, 1H, H-3), 8.63 (s, 1H, H-6) ppm (Supplementary Materials: Spectrum 6.); ^13^C NMR (DMSO-*d_6_*, 125 MHz) δ 118.72, 121.90, 129.70, 131.23, 131.59, 131.75, 133.55, 134.38, 134.72, 136.15, 141.00, 161.28, 163.98 ppm (Supplementary Materials: Spectrum 7.). Anal. C 41.31, H 1.95, N 9.03% calcd for C_16_H_9_Cl_4_N_3_O_3_S (465.13), C 41.12, H 2.13, N.8.51 % 

(*E*)-5-(5-(4-Bromostyryl)-1,3,4-oxadiazol-2-yl)-2,4-dichlorobenzenesulfonamide (**15**). 

Using 4-bromobenzaldehyde (0.11 g) and crystalizing the crude product from glacial acetic acid, the title compound **15** was obtained (0.12 g, 48%) as a pale yellow powder: mp 249-253 °C decomp; IR (KBr) ν_max_: 3307, 3200 (NH); 3092 (C_Ar_-H); 1632, 1586, 1564 1528, (C=C, C=N); 1357, 1172 (SO_2_) cm^-1^; ^1^H NMR (DMSO-*d*_6_, 500 MHz) δ: 7.50 (d, *J*=16.5 Hz, 1H, C=CH), 7.63-7.69 (m, 3 H, H-3,5Ph, CH=C), 7.78 (d, 2H, H-2,6Ph), 7.97 (s, 2 H, NH_2_), 8.16 (s, 1H, H-3), 8.62 (s, 1H, H-6) ppm

Anal. C 40.44, H 2.12, N 8.84 % calcd for C_16_H_10_BrCl_2_N_3_O_3_S (475.14), C 40.37, H 2.11, N 8.84 % 

(*E*)-2,4-Dichloro-5-(5-(4-hydroxystyryl)-1,3,4-oxadiazol-2-yl)benzenesulfonamide (**17**). 

Using 4-hydroxybenzaldehyde (0.073 g) and crystalizing the crude product from ethanol, the title compound **17** was obtained (0.7 g, 35%) as white plates: mp 251-256 °C decomp; IR (KBr) ν_max_: 3345 (OH), 3224 (NH), 3088, 3077, 3051, 3020 (C_Ar_-H), 1637, 1604, 1591, 1560, 1531 (C=C, C=N), 1337, 1164 (SO_2_), 1233 (C-O) cm^−1^; ^1^H NMR (DMSO-*d*_6_, 500 MHz) δ: 6.83 (d, *J*=8.3 Hz, 2H, H-3,5 phenol), 7.20 (d, *J*=16.6 Hz, 1H, C=CH), 7.61 (d, *J*=16.5 Hz, 1H, C=CH), 7.66 (d, *J*=8,7Hz, 2H, H-2,6 phenol), 7.97 (s, 2H, NH_2_ ), 8.18 (s, 1H, H-3), 8.61 (s, 1H, H-6), 10.04 (s, 1H, OH) ppm. (Supplementary Materials: Spectrum 8.); ^13^C NMR (DMSO-*d_6_*, 125 MHz) δ 106.31, 116.30, 122.15, 126.07, 130.45, 130.97, 134.32, 135.95, 140.46, 140.98, 160.10, 160.31, 165.56 ppm (Supplementary Materials: Spectrum 9.). Anal. C 46.62, H 2.69, N 10.19 % calcd for C_16_H_11_Cl_2_N_3_O_4_S (412.25), C 46.45, H 2.70, N 10.06 % 

(*E*)-2-(Benzylthio)-4-chloro-5-(5-(4-hydroxystyryl)-1,3,4-oxadiazol-2-yl)benzenesulfonamide (**18**). 

Using 4-hydroxybenzaldehyde (0.07 g) and crystalizing the crude product from glacial acetic acid, the title compound **18** was obtained (0.13 g, 51%) as a yellow powder: mp 249–250 °C; IR (KBr) ν_max_ 3372 (OH), 3277 (NH), 3029, 3061 (C_Ar_-H), 2960, 2925, 2854 (C-H), 1635, 1604, 1584, 1527 (C=C, C=N), 1344, 1170 (SO_2_), 1251 (C-O) cm^−1^; ^1^H NMR (500 MHz, DMSO-d_6_) δ: 4.52 (s, 2H, CH_2_), 6.82 (d, 2H, H-3,5 phenol), 7.16 (d, 1H, C=CH), 7.30 (t, 1H, H-4 Ph), 7.36 (t, 2H, H-3,5 Ph), 7.47( d, 2H, H-2,6 Ph), 7.57 (d, 1H, C=CH), 7.63 (d, 2H, H-2,6 phenol), 7,80 (s, 3H, NH_2_, H-3), 8.47 (s, 1H, H-6), 10.03 (s, 1H, OH) ppm. Anal. C 55.25, H 3.63, N 8.40 % calcd for C_23_H_18_ClN_3_O_4_S_2_ (3.2), C 53.23, H 3.71, N 8.03 %

(*E*)-2,4-Dichloro-5-(5-(4-cyanostyryl)-1,3,4-oxadiazol-2-yl)benzenesulfonamide (**19**). 

Using 4-cyanobenzaldehyde (0.08 g) and crystalizing the crude product from glacial acetic acid, the title compound **19** was obtained (0.15 g, 72%) as a light yellow fluffy powder: mp 289-294 °C decomp.; IR (KBr) ν_max_ 3364, 3265 (NH), 3091 (C_Ar_-H), 2229(C≡N), 1630, 1601, 1586, 1543, 1531 (C=C, C=N), 1353, 1175 (SO_2_) cm^−1^; ^1^H NMR (DMSO-*d*_6_, 500 MHz) δ: 7.68 (d, *J*=16.6 Hz, 1H, CH=C), 7.77 (d, J=16,6 Hz, 1H, CH=C), 7.91 (d, *J*=8.3 Hz, 2H, H-2,6 Ph), 7.97 (brs, 2H, NH_2_), 8.03 (d *J*=8.3 Hz, 2H, H-3,5), 8.17 (s, 1H, H-3), 8.63 (s, 1H, H-6) ppm; ^13^C NMR (DMSO-*d_6_*, 125 MHz) δ: 112.42, 113.66, 119.09, 121.94, 129.14, 131.16, 133.25, 134.35, 134.65, 136.15, 138.22, 139.48, 140.99, 161.03, 164.70 ppm. Anal. C 48.47, H 2.39, N 13.30 % calcd for C_17_H_10_Cl_2_N_4_O_3_S (3.2), C 48.19, H 2.35, N 13.11% 

(*E*)-2-(Benzylthio)-4-chloro-5-(5-(4-cyanostyryl)-1,3,4-oxadiazol-2-yl)benzenesulfonamide (**20**). 

Using 4-cyanobenzaldehyde (0.08 g) and crystalizing the crude product from glacial acetic acid, the title compound **20** was obtained (0.16 g, 64%) as a white powder: mp 262-263°C; IR (KBr) ν_max_: 3411, 3293 (NH), 3045 (C_Ar_-H), 2226 (C≡N), 1643, 1604, 1579, 1558, 1521 (C=C, C=N), 1341, 1171 (SO_2_) cm^−1^; ^1^H NMR (DMSO-*d*_6_, 500 MHz) δ: 4.53 (s, 2H, CH_2_), 7.30 (t, 1H, H-4 Ph), 7.36 (t, 2H, H-3,5 Ph), 7.47 ( d, 2H, H-2,6 Ph), 7.65 (d, 1H, CH=C), 7.74 (d, 1H, CH=C), 7.77 (s, 2H, NH_2_), 7.81 (s, 1H, H-3), 7.90 (d, 2H, H-2,6), 8.00 (d, 2H, H-3,5), 8.49 (s, 1H, H-6) ppm; Anal. C 56.63, H 3.37, N 11.01 % calcd for C_24_H_17_ClN_4_O_3_S_2_ (509.00), C 56.01, H 3.14, N 10.75 %

(*E*)-2,4-Dichloro-5-(5-(4-(trifluoromethyl)styryl)-1,3,4-oxadiazol-2-yl)benzenesulfonamide (**21**). 

Using 4-trifluoromethylbenzaldehyde (0.11 g) and crystalizing the crude product from ethanol, the title compound **21** was obtained (0.14 g, 60%) as a white solid: mp 248–250 °C; IR (KBr) ν_max_: 3414 (NH), 3084, 3064 (C_Ar_-H), 1639,1582, 1532, 1440 (C=C), (C=N), 1335, 1172 (SO_2_), 1116 (C-F) cm^−1^; ^1^H NMR (DMSO-*d*_6_, 500 MHz) δ: 7.65 (d, *J*=16.6Hz, 1H, C=CH), 7.78-7.82 (m, 3H, C=CH, H-3,5 Ph), 7.98 (s, 2H, NH_2_), 8.06 (d, *J*=7.8 Hz, 2H, H-2,6), 8.18 (s, 1H, H-6), 8.64 (s, 1H, H-6) ppm; ^13^C NMR (DMSO-*d_6_*, 125 MHz) δ: 113.04, 121.97, 126.22 (k), 129.13, 131.14, 134.35, 134.63, 136.14, 138.43,138.96, 141.00, 160.97, 164.74 ppm; Anal. C 43.98, H 2.17, N 9.05 % calcd for C_17_H_10_Cl_2_F_3_N_3_O_3_S (464.25), C 43.87, H 2.15, N 8.94% 

(*E*)-2-(Benzylthio)-4-chloro-5-(5-(4-(trifluoromethyl)styryl)-1,3,4-oxadiazol-2-yl)benzenesulfonamide (**22**). 

Using 4-trifluorobenzaldehyde (0.11 g) and crystalizing the crude product from glacial acetic acid, the title compound **22** was obtained (0.16 g, 58%) as an off-white powder: mp 248–250 °C; IR (KBr) ν_max_ 3297 (NH), 3096, 3027 (C_Ar_-H), 2955, 2923, 2854 (C-H), 1641,1578, 1522, 1455 (C=C), (C=N), 1327, 1170 (SO_2_), 1128 (C-F) cm^−1^; ^1^H NMR (DMSO-*d*_6_, 500 MHz) δ: 4.53 (s, 2H, CH_2_), 7.30 (t, 1H, H-4 Ph), 7.37 (t, 2H, H-3,5 Ph), 7.47 (d, 2H, H-2,6 Ph), 7.63 (d, *J*=16.6 Hz 1H, C=CH), 7.76 (d, *J*=16.6 Hz, 1H, C=CH), 7.79-7.82 (m, 5H, NH_2_, H-3, H-3,5 Ar), 8.04 (d, 2H, H-2,6), 8,50 (s, 1H, H-6) ppm; Anal. C 52.22, H 3.10, N 7.61 % calcd for C_24_H_17_ClF_3_N_3_O_3_S_2_ (551.99), C 51.65, H 2.93, N 7.59%

(*E*)-2,4-Dichloro-5-(5-(2-(pyridin-4-yl)vinyl)-1,3,4-oxadiazol-2-yl)benzenesulfonamide (**23**). 

Using 4-pyridinecarboxaldehyde (0.07 g) and crystalizing the crude product from glacial acetic acid, the title compound **23** was obtained (0.11 g, 55%) as a white solid: mp 274 °C decomp.; IR (KBr) ν_max_:3244 (NH), 3085, 3069, 3031 (C_Ar_-H), 1641, 1603, 1584, 1560, 1525, 1524 (C=C, C=N), 1350, 1167 (SO_2_) cm^−1^; ^1^H NMR (DMSO-*d*_6_, 500 MHz) δ: 7.65 (d, *J*=16 Hz, 1H, C=CH), 7.69 (d, *J*=16 Hz, 1H, C=CH), 7.73-7.74 (m, 4H, NH_2_, H-3,5piryd), 8.09 (s, 1H, H-3), 8.65-8.66 (m, 3H, H-6, H-2,6piryd) ppm; ^13^C NMR (DMSO-*d_6_*, 125 MHz) δ: 114.72, 122.06, 122.21, 131.40, 134.22, 134.86, 136.19, 137.75, 141.30, 142.10, 150.80, 161.19, 164.59 ppm. Anal. C 45.35, H 2.54, N 14.10 % calcd for C_15_H_10_Cl_2_N_4_O_3_S (397.24), C 45.17, H 2.55, N 13.85%

(*E*)-2-(Benzylthio)-4-chloro-5-(5-(2-(pyridin-4-yl)vinyl)-1,3,4-oxadiazol-2-yl)benzenesulfonamide (**24**). 

Using 4-pyridinecarboxaldehyde (0.07 g) and crystalizing the crude product from glacial acetic acid, the title compound **24** was obtained (0.18 g, 74%) as an off-white powder: mp 265-266 °C; IR (KBr) ν_max:_ 3333, 3275 (NH), 3055, 3030 (C_Ar_-H), 2928 (C-H), 1635, 1600, 1584, 1556, 1523 (C=C, C=N), 1340, 1170 (SO_2_) cm^−1^; ^1^H NMR (DMSO-*d*_6_, 500 MHz) δ: 4.53 (s, 2H, CH_2_), 7.30 (t, 1H, H-4 Ph), 7.37 (t, 2H, H-3,5 Ph), 7.48 (d, 2H, H-2,6 Ph), 7.68(d, 1H, C=CH), 7.78 (d, 1H, C=CH), 7.80-7.83 (m, 5H, NH_2_, H-3, H-3,5 piryd), 8.50 (s, 1H, H-6), 8.68 (d, 2H, H-2,6 piryd.) ppm; Anal. C 55.48, H 3.53, N 11.55 % calcd for C_22_H_17_ClN_4_O_3_S_2_ (484.98), C 52.71, H 3.31, N 11.09%

(*E*)-2,4-Dichloro-5-(5-(2-(pyridin-3-yl)vinyl)-1,3,4-oxadiazol-2-yl)benzenesulfonamide (**25**). 

Using 3-pyridinecarboxaldehyde (0.07 g) and crystalizing the crude product from glacial acetic acid, the title compound **25** was obtained (0.11 g, 55%) as a pale yellow powder: mp 251-255 °C dec; IR (KBr) ν_max_:3173 (NH), 3076 (C_Ar_-H), 1633, 1585, 1561, 1525, 1523 (C=C, C=N), 1363, 1173 (SO_2_) cm^−1^; ^1^H NMR (DMSO-*d*_6_, 500 MHz) δ: 7.48 (d.d, 1H, H-5 piryd.), 7.63 (d, 1H, C=CH), 7.74 (d, 1H, C=CH), 7,97 (s, 2H, NH_2_), 8.18 (s, 1H, H-3), 8.30 (d, 1H, H-4 piryd.), 8.59 (d., 1H, H-6 piryd.), 8.62 (s, 1H, H-6), 8.97 (d, 1H, H-2 piryd.) ppm; ^13^C NMR (DMSO-*d_6_*, 125 MHz) δ: 112.14, 121.99, 124.42, 130.84, 131.13, 134.34, 134.60, 134.75, 136.12, 136.92, 140.98, 150.11, 151.13, 160.87, 164.80 ppm. Anal. C 45.35, H 2.54, N 14.10 % calcd for C_15_H_10_Cl_2_N_4_O_3_S (397.24), C 45.00, H 2.52, N 13.85%

(*E*)-2-(Benzylthio)-4-chloro-5-(5-(2-(pyridin-3-yl)vinyl)-1,3,4-oxadiazol-2-yl)benzenesulfonamide (**26**). 

Using 3-pyridinecarboxaldehyde (0.07 g) and crystalizing the crude product from glacial acetic acid, the title compound **26** was obtained (0.14 g, 70%) as a white powder: mp 273-275 °C; IR (KBr) ν_max_: 3374, 3266 (NH), 3061, 3030 (C_Ar_-H), 2925 (C-H), 1642, 1583, 1521 (C=C, C=N), 1353, 1168 (SO_2_) cm^−1^; ^1^H NMR (DMSO-*d*_6_, 500 MHz) δ: 4.53 (s, 2H, CH2), 7.30 (t, 1H, H-4 Ph), 7.37 (t, 2H, H-3,5 Ph), 7.48 (2H, H-2,6 Ph), 7.52 (d.d, 1H, H-5 piryd.), 7.62 (d, 1H, C=CH), 7.72 (d, 1H, C=CH), 7.80 (s, 2H, NH_2_), 7.82 (s, 1H, H-3), 8.32 (d, 1H, H-4 piryd.), 8.49 (s, 1H, H-6), 8.61 (d.d., 1H, H-6 piryd.), 8.97 (d, 1H, H-2 piryd.) ppm; Anal. C 55.48, H 3.53, N 11.55 % calcd for C_22_H_17_ClN_4_O_3_S_2_ (484.98), C 54.98, H 3.44, N 10.75%

(*E*)-2,4-Dichloro-5-(5-(2-(pyridin-2-yl)vinyl)-1,3,4-oxadiazol-2-yl)benzenesulfonamide (**27**). 

Using 2-pyridinecarboxaldehyde (0.07 g), the crude product was first crystalized from acetonitrile to give E/Z mixture 1:1, and thus it was dissolved in 6 mL of glacial acetic acid and refluxed for 24 h; next, solvent was evaporated, 1 mL of acetonitrile was added and the precipitate formed was filtered off. In this way, the title compound **27** was obtained (0.13 g, 64%) as an off-white powder: mp 252–256 °C decomp.; IR (KBr) ν_max_: 3387, 3348, 3247 (NH), 3087, 3018 (C_Ar_-H), 1586, 1562, 1441, (C=C, C=N); 1339, 1168 (SO_2_) cm^−1^; ^1^H NMR (DMSO-*d*_6_, 500 MHz) δ: 7.43 (dt, 1H, H-5 piryd.), 7.68 (d, *J*=16.1Hz, 1H, C=CH), 7.76 (d *J*=16.1Hz, 1H, C=CH), 7.85 (d, 1 H, H-3 piryd.), 7.91 (dt, 1H, H-4 piryd.), 7.98 (s, 2H, NH_2_), 8.19 (s, 1H, H-3), 8.64 (s,1H, H-6), 8,68 (d, 1H, H-6 piryd.) ppm (Supplementary Materials: Spectrum 10.); ^13^C NMR (DMSO-*d_6_*, 125 MHz) δ: 115.32, 124.13, 127.04, 127.26, 133.34, 136.56, 136.83, 138.31, 140.01, 141.62, 143.20, 152.69, 154.88, 163.23, 166.86 ppm (Supplementary Materials: Spectrum 11.). Anal. C 45.35, H 2.54, N 14.10 % calcd for C_15_H_10_Cl_2_N_4_O_3_S (397.24), C 45.16, H 2.53, N 13.91 %

(*E*)-2-(Benzylthio)-4-chloro-5-(5-(2-(pyridin-2-yl)vinyl)-1,3,4-oxadiazol-2-yl)benzenesulfonamide (**28**). 

Using 2-pyridinecarboxaldehyde (0.07 g) and crystalizing the crude product from glacial acetic acid, the title compound **28** was obtained (0.12 g, 50%) as a white fluffy powder: mp 254-257 °C decomp.; IR (KBr) ν_max_: 3321 (NH), 3057, 3027 (C_Ar_-H), 2929 (C-H), 1586, 1526 (C=C, C=N), 1347, 1145 (SO_2_) cm^−1^; ^1^H NMR (DMSO-*d*_6_, 500 MHz) δ: 4.54 (s, 2H, CH2), 7.30 (t *J* = 7.4 Hz, 1H, H-4 Ph), 7.37 (t, *J* = 7.4 Hz, 2H, H-3,5 Ph), 7.42 (dt, *J* = 6.0 Hz *J* = 1.6 Hz 1H, H-5 piryd.), 7.48 (d, *J* = 7.4 Hz 2H, H-2,6 Ph), 7.65 (d, *J* = 16.0 Hz, 1H, C=CH), 7.73 (d *J*=16.0 Hz, 1H, C=CH), 7.81-7.84 (m, 4H, NH_2_, H3, H-3 piryd.), 7.89 (dt, *J* = 7.7 Hz *J* = 1.6 Hz, 1H, H-4 piryd.), 8.51 (s, 1H, H-6), 8.67 (d, *J* = 4.1 Hz 1H, H-6 piryd.) ppm (Supplementary Materials: Spectrum 12.); ^13^C NMR (DMSO-*d_6_*, 125 MHz) δ: 36.44, 113.21, 118.83, 124.68, 125.01, 128.07, 129.02, 129.16, 129.65, 129.97, 133.94, 135.91, 137.62, 137.78, 139.30, 145.51, 150.50, 152.77, 161.65, 163.94 ppm (Supplementary Materials: Spectrum 13.). Anal. C 55.48, H 3.53, N 11.55 % calcd for C_22_H_17_ClN_4_O_3_S_2_ (484.98), C 54.27, H 3.35, N 11.39 %

(*E*)-2,4-Dichloro-5-(5-(2-(thiophen-2-yl)vinyl)-1,3,4-oxadiazol-2-yl)benzenesulfonamide (**29**). 

Using thiophene-2-carbaldehyde (0.07 g) and crystalizing the crude product from acetonitrile, the title compound **29** was obtained (0.12 g, 65%) as colorless plates: mp 264 °C decomp.; IR (KBr) ν_max_: 3389, 3352, 3245 (NH), 3095 (C_Ar_-H), 1632, 1561 (C=C, C=N), 1339, 1167 (SO_2_) cm^-1^; ^1^H NMR (DMSO-*d*_6_, 500 MHz) δ: 7.08 (d, *J*=16 Hz, 1H, C=CH), 7.18 (dd, *J*=3 Hz *J*=5 Hz, 1H, H4thiophene), 7.64 (d, *J*=3Hz 1H, thiophene), 7.75 (d, *J*=5 Hz, 1H, tiophene), 7.88 (d, *J*=16 Hz, 1H, C=CH), 7.96 (s, 2H, NH_2_), 8.17 (s, 1H, H-3), 8.61 (s, 1H, H-6) ppm; Anal. C 41.80, H 2.26, N 10.45% calcd for C_14_H_9_Cl_2_N_3_O_3_S_2_ (402.28), C 41.93, H 2.20, N 10.43 %

(*E*)-2-(Benzylthio)-4-chloro-5-(5-(2-(thiophen-2-yl)vinyl)-1,3,4-oxadiazol-2-yl)benzenesulfonamide (**30**). 

Using thiophene-2-carbaldehyde (0.07 g) and crystalizing the crude product from acetonitrile, the title compound **30** was obtained (0.14 g, 58%) as a pale yellow powder: mp 240-242 °C; IR (KBr) ν_max_: 3373, 3317, 3275 (NH), 3093, 3030 (C_Ar_-H), 2943, 2925 (C-H), 1630, 1583, 1537, 1526 (C=C, C=N), 1339, 1172 (SO_2_) cm^−1^; ^1^H NMR (DMSO-*d*_6_, 500 MHz) δ: 4.52 (s, 2H, CH_2_), 7.06 (d, 1H, C=CH), 7.17 (t, 1H, H-4 Ph), 7.30 (t, 1H, H-4 tiophene), 7.36 (t, 2H, H-3,5 Ph), 7.47 (d, 2H, H-2,6 Ph), 7.62 (d, 1H, H-3 tiophene), 7.75 (d, 1H, H-5 tiophene); 7.79 (s, 2H, NH_2_), 7.81 (s, 1H, H-3), 7.83 (d, 1H, C=CH), 8.47 (s, 1H, H-6) ppm. Anal. C 51.47, H 3.29, N 8.58% calcd for C_21_H_16_ClN_3_O_3_S_3_ (490.02), C 50.45, H 3.30, N 8.45 %

(*E*)-2,4-Dichloro-5-(5-(2-(5-nitrothiophen-2-yl)vinyl)-1,3,4-oxadiazol-2-yl)benzenesulfonamide (**31**). 

Using 5-nitrothiophene-2-carbaldehyde (0.10 g) and crystalizing the crude product from glacial acetic acid the title compound **31** was obtained (0.16 g, 71%) as yellow powder: mp 272–276 °C decomp.; IR (KBr) ν_max_ 3384, 3278 (NH), 3102, 3039 (C_Ar_-H), 1634, 1590, 1543, 1489 (C=C, C=N), 1512,1284 (NO_2_), 1333, 1170 (SO_2_) cm^−1^; ^1^H NMR (DMSO-*d*_6_, 500 MHz) δ: 7.61 (d, *J*=16.6 Hz, 1H, C=CH), 7.76 (d, *J* = 4.4 Hz 1H, H-3 thiofene), 7.97 (d, *J*=16.6 Hz, 1H, C=CH), 7.97 (s, 2H, NH_2_), 8.15 (d, *J* = 4.3 Hz, 1H, H-4 thiofene), 8.18 (s, 1H, H-3), 8.63 (s, 1H, H-6) ppm (Supplementary Materials: Spectrum 14.); ^13^C NMR (DMSO-*d_6_*, 125 MHz) δ: 114.13, 121.86, 130.09, 131.14, 131.17, 131.30, 134.38, 134.72, 136.18, 140.98, 146.83, 151.86, 161.18, 164.19 ppm. Anal. C 37.59, H 1.80, N 12.53% calcd for C_14_H_8_Cl_2_N_4_O_5_S_2_ (447.27), C 37.50, H 1.79, N 12.07 % 

(*E*)-2-(Benzylthio)-4-chloro-5-(5-(2-(5-nitrothiophen-2-yl)vinyl)-1,3,4-oxadiazol-2-yl)benzenesulfonamide (**32**). 

Using 5-nitrothiophene-2-carbaldehyde (0.10 g) and crystalizing the crude product from glacial acetic acid, the title compound **32** was obtained (0.25 g, 95%) as a yellow powder: mp 273-275 °C; IR (KBr) ν_max_ 3365, 3255 (NH), 3102, 3043 (C_Ar_-H), 1583, 1510, 1495 (C=C, C=N), 1518,1307 (NO_2_), 1337, 1172 (SO_2_) cm^−1^; ^1^H NMR (DMSO-*d*_6_, 500 MHz) δ: 4.53 (s, 2H, CH_2_), 7.30 (t, *J* = 7.6 Hz, 1H, H-4 Ph), 7.36 (t, *J* = 7.6 Hz, 2H, H-3,5 Ph), 7.47( d, *J* = 7.3 Hz, 2H, H-2,6 Ph), 7.59 (d, *J* = 16.6 Hz, 1H, C=CH), 7.75 (d, *J* = 4.4 Hz, 1H, H-3thiophene), 7.78 (s, 2H, NH_2_), 7.82 (s, 1H, H-3), 7.89 (d, *J* = 16.2 Hz 1H, C=CH), 8.16 (d, *J* = 4.3 Hz, 1H, H-4 thiophene), 8.48 (s, 1H, H-6) ppm (Supplementary Materials: Spectrum 15.); Anal. C 47.14, H 2.83, N 10.47% calcd for C_16_H_11_Cl_2_N_3_O_3_S (535.02), C 46.79, H 2.67, N 10.32%

(*E*)-2,4-Dichloro-5-(5-(2-(furan-2-yl)vinyl)-1,3,4-oxadiazol-2-yl)benzenesulfonamide (**33**). 

Using furan-2-carbaldehyde (0.06 g) and crystalizing the crude product from glacial acetic acid, the title compound **33** was obtained (0.13 g, 62%) as pale brown plates: mp 248–250 °C; IR (KBr) ν_max_: 3385, 3344, 3242 (NH), 3087 (C_Ar_-H), 1641, 1581, 1562 (C=C, C=N), 1339, 1168 (SO_2_) cm^−1^; ^1^H NMR (DMSO-*d*_6_, 500 MHz) δ: 6.68 (dd, *J*=1.9 Hz J= 3.4Hz, 1H, H-4furan), 6.98 (d, *J*=16.6 Hz, 1H, C=CH), 7.02 (d, J=3.5 Hz 1H, furan), 7,54 (d, *J*=16.6 Hz, 1H, C=CH), 7.90 (s, 1H, furan), 7.96 (s, 2H, NH_2_), 8.17 (s, 1H, H-3), 8.62 (s, 1H, H-6) ppm; ^13^C NMR (DMSO-*d_6_*, 125 MHz) δ: 106.76, 113.40, 115.77, 121.99, 127.14, 131.02, 134.35, 134.47, 135.99, 140.99, 146.35, 150.92, 160.62, 164.86 ppm. Anal. C 43.54, H 2.35, N 10.88% calcd for C_14_H_9_Cl_2_N_3_O_4_S (386.21), C 43.38, H 2.25, N 10.91 % 

(*E*)-2-(Benzylthio)-4-chloro-5-(5-(2-(furan-2-yl)vinyl)-1,3,4-oxadiazol-2-yl)benzenesulfonamide (**34**). 

Using furan-2-carbaldehyde (0.06 g) and crystalizing the crude product from glacial acetic acid, the title compound **34** was obtained (0.104 g, 54%) as an off-white powder: mp 238 °C decomp.; IR (KBr) ν_max_: 3355, 3260 (NH), 3090 (C_Ar_-H), 2925 (C-H), 1625, 1584, 1543, 1535 (C=C, C=N), 1356, 1155 (SO_2_) cm^−1^; ^1^H NMR (DMSO-*d*_6_, 500 MHz) δ: 4.53 (s, 2H, CH_2_), 7.08 (d, *J*=16.1 Hz, 1H, C=CH), 7.18 (t, 1H, H-4 Ph), 7.30 (t, 1H, H-4 furan), 7.37(t, 2H, H-3,5 Ph), 7.48( d, 2H, H-2,6 Ph), 7.63 (d, 1H, H- 3 furan), 7.76 (d, 1H, H-5 furan), 7.80 (s, 1H, NH_2_), 7.82 (s, 1H, H-3), 7.84 (d, *J*=16.1 Hz, 1H, C=CH), 8.48 (s, 1H, H-6) ppm; Anal. C 51.17, H 3.15, N 8.42% calcd for C_21_H_16_ClN_3_O_4_S_2_ (473.95), C 53.22, H 3.40, N 8.87 % 

(*E*)-2-(Benzylthio)-4-chloro-5-(5-(2-(5-nitrofuran-2-yl)vinyl)-1,3,4-oxadiazol-2-yl)benzenesulfonamide (**35**). 

Using 5-nitrofuran-2-carbaldehyde (0.09 g), the crude product was purified by quick heating to reflux in 10 mL of acetonitrile followed by 24 h of stirring at room temperature and then the precipitate was filtered off, thus the title compound **36** was obtained (0.15 g, 58%) as a yellow solid: mp 255–257 °C decomp.; IR (KBr) ν_max_: 3364, 3269, 3142 (NH), 3068, 3028 (C_Ar_-H), 2927, 2848, 2800 (C-H), 1584, 1519, 1505, 1458 (C=C, C=N); 1537,1393 (NO_2_) 1347, 1174 (SO_2_) cm^−1^; ^1^H NMR (DMSO-*d*_6_, 500 MHz) δ: 4.54 (s, 2H, CH_2_), 7.30-7.48 (m, 7 H, H-4 Ph, H-3,5 Ph, H-2,6 Ph C=CH, H-3 furan), 7.62 (d, *J*=16Hz, 1H, C=CH), 7.80-7.82 (m, 4H, H-4 furan. NH_2_, H-3), 8.51 (s, 1H, H-6) ppm (Supplementary Materials: Spectrum 16.); ^13^C NMR (DMSO-*d_6_*, 125 MHz) δ: 36.47, 113.88, 115.42, 116.70, 118.63, 125.19, 128.09, 129.02, 129.17, 129.66, 130.03, 134.08, 135.87, 137.61, 145.65, 152.36, 153.34, 161.91, 163.32 ppm (Supplementary Materials: Spectrum 17.). Anal. C 48.60, H 2.91, N 10.80% calcd for C_21_H_15_ClN_4_O_6_S_2_ (518.95), C 48.54, H 2.90, N 10.68 %

(*E*)-5-(5-(2-(1H-Pyrrol-2-yl)vinyl)-1,3,4-oxadiazol-2-yl)-2,4-dichlorobenzenesulfonamide (**36**). 

Using 1*H*-pyrrole-2-carbaldehyde (0.06 g), the crude product was purified by quick heating to reflux in 5 mL of acetonitrile followed by 24 h of stirring in room temperature and then filtered off the precipitate, the title compound **36** was obtained (0.08 g, 43%) as a yellow solid: mp 260 °C decomp.; IR (KBr) ν_max_: 3376, 3267 (NH), 3097 (C_Ar_-H), 1625, 1591, 1572, 1457 (C=C, C=N), 1348, 1163 (SO_2_) cm^−1^; ^1^H NMR (DMSO-*d*_6_, 500 MHz) δ: 6.19 (d, *J*=2.5Hz, 1H, pyrrole), 6.64 (s, 1H, pyrrole), 6.93 (d, *J*=16Hz, 1H, C=CH), 7.07 (s, 1H, pyrrole), 7,48 (d, *J*=16Hz, 1H, C=CH), 7.95 (s, 2H, NH_2_), 8.15 (s, 1H, H-3), 8.59 (s, 1H, H-6), 11.62 (s, 1H, NH pyrrole) ppm (Supplementary Materials: Spectrum 18.); ^13^C NMR (DMSO-*d_6_*, 125 MHz) δ: 102.16, 110.67, 114.94, 122.17, 123.93, 129.01, 130.55, 130.84, 134.15, 134.31, 135.76, 140.95, 159.97, 165.80 ppm (Supplementary Materials: Spectrum 19.). Anal. C43.65, H 2.62, N 14.54 % calcd for C_14_H_10_Cl_2_N_4_O_3_S (385.23), C 43.02, H 2.60, N 14.31 %.

#### 3.1.4. Procedure for the Preparation of 4-(5-(Chloromethyl)-1,3,4-Oxadiazol-2-yl)Benzenesulfonamide (**43**)

A mixture of 4-(carbazoyl)benzenesulfonamide (**41**) (4.64 g, 21 mmol) and 2-chloro-1,1,1-trimethoxyethane (4.95 g, 32 mmol) in a mixture of 1,4-dioxane (100 mL) and glacial acetic acid (7.5 mL) was refluxed for 14 h. Next, the solvent was evaporated under reduced pressure, acetonitrile (18 mL) was added and the suspension was stirred vigorously at room temperature for 1 h. The precipitate was filtered off, washed with cold ethanol and dried. Yield 4.16 g (71%) as white needles; mp 201 – 205 °C; (KBr) ν_max_: 3283, 3159 (NH); 3090, 3034 (C_Ar_-H); 2975 (C-H), 1570, 1552 (C=C, C=N); 1350, 1171 (SO_2_) cm^−1^; ^1^H NMR (DMSO-*d*_6_, 500 MHz) δ: 5.17 (s, 2H, CH_2_); 7.61 (s, 2H, NH_2_); 8.05 (d, *J* = 8.3 Hz, 2H, H-2,6); 8.22 (d, *J* = 8.3 Hz, 2H, H-3,5) ppm (Supplementary Materials: Spectrum 20.); ^13^C NMR (DMSO-*d_6_*, 125 MHz) δ: 33.66; 126.11; 127.28; 127.91; 147.54; 163.87; 164.57 ppm (Supplementary Materials: Spectrum 21.). Anal. C 39.50 H 2.95, N 15.35% calcd for C_9_H_8_ClN_3_O_3_S (273.70), C 38.95, H 2.88, N 15.01%

#### 3.1.5. Procedure for the Preparation of ((5-(4-Sulfamoylphenyl)-1,3,4-Oxadiazol-2-yl)Methyl)Triphenylphosphonium Chloride (**45**)

A mixture of compound **43** (3.28 g, 12 mmol) and triphenylphosphine (3.59 g, 13.7 mmol) in dry acetonitrile (120 mL) was refluxed for 24 h. After cooling to room temperature, the precipitated sticky solid was filtered off, suspended in anhydrous acetone and stirred for 1 h, filtered off and dried. 

Yield 3.20 g (52%) as a white powder, mp 194°C decomp.; IR (KBr) ν_max_ 3376, 3146 (NH); 3055, 3010 (C_Ar_-H), 1586, 1549 (C=C, C=N), 1334, 1161 (SO_2_) cm^−1^; ^1^H NMR (DMSO-*d_6_*, 500 MHz) δ 6.08 (d, 2H, CH_2_); 7.64 (s, 2H, NH_2_); 7.79-7.83 (m, 6H, Ar); 7.88-7.94 (m, 11H, Ar ); 8.00 (d, 2H, H-3,5) ppm. Anal. C 60.50, H 4.33, N 7.84% calcd for C_27_H_23_ClN_3_O_3_PS (535.98), C 60.71, H 3.87, N 7.40%

#### 3.1.6. Procedure for the Preparation of ((5-(2,4-Dichlorophenyl)-1,3,4-Oxadiazol-2-yl)Methyl)Triphenylphosphonium Chloride (**46**)

A mixture of compound **44** (2.7 g, 10 mmol) and triphenylphosphine (2.88 g, 11 mmol) in dry acetonitrile (120 mL) was refluxed for 45 h. Next, the solvent was evaporated to half under reduced pressure, and after cooling to 4°C the precipitate solid was filtered off and dried. Yield 4.38 g (85%) as a white powder, mp 115 °C decomp.; IR (KBr) ν_max_ 3482, 3412 (P^+^Cl^-^); 3044 (C_Ar_-H), 2838, 2767 (C-H); 1604, 1572, 1556 (C=C, C=N) cm^−1^; ^1^H NMR (DMSO-*d_6_*, 500 MHz) δ 6.08 (d, 2H, CH_2_); 7.66 (d.d., 2H, H-5); 7.75 (d, 1H, H-6); 7.78-7.82 (m, 6H, Ar); 7.86-7.96 (m, 10H, Ar) ppm. Anal. C 61.68, H 3.83, N 5.33% calcd for C_27_H_20_Cl_3_N_2_OP (525.79), C 61.21, H 3.70, N 4.97%

#### 3.1.7. General Procedure for the Preparation of (*E*)-4-(5-(2-arylvinyl)-1,3,4-Oxadiazol-2-yl)Benzenesulfonamides (**47**–**50**)

Triphenylphosphonium chloride **45** (0.40 g, 0.75 mmol) and appropriate aldehyde (0.9 mmol) in 20 mL of dry dichloromethane was put under nitrogen atmosphere and a solution of *N*,*N*-diisopropylethylamine (0.19 g, 1.5 mmol) in 5 mL of dichloromethane was added. The reaction mixture was stirred at room temperature for 24 h. Next, the solvent was evaporated under reduced pressure, ethanol (6 mL) was added and the suspension was stirred vigorously at room temperature for 1 h. The precipitate was filtered off and the crude product was purified as follows:

(*E*)-4-(5-Styryl-1,3,4-oxadiazol-2-yl)benzenesulfonamide (**47**).

Using benzaldehyde (0.10 g) and crystalizing the crude product from glacial acetic acid, the title compound was obtained. Yield 0.13 g (54%) as a white fluffy powder: mp 295-297 °C decomp.; IR (KBr) ν_max_ 3308 (NH); 3057 (C_Ar_-H), 1636, 1599, 1553, 1524 (C=C, C=N); 1343, 1160 (SO_2_) cm^−1^; ^1^H NMR (DMSO-*d_6_*, 500 MHz) δ 7.42-7.45 (d, *J*=16.6 Hz, 1H, C=CH), 7.44-7.48 (m, 3H, H-4′, H-3′,5′); 7.61 (s, 2H, NH_2_); 7.80-7.83 (m, 3H, H-2′,6′ =CH); 8.06 (d, *J* = 8.3 Hz, 2H, H-2,6); 8.30 (d, *J* = 8.3 Hz, 2H, H-3,5) ppm (Supplementary Materials: Spectrum 22.). ^13^C NMR (DMSO-*d_6_*, 125 MHz) δ: 110.28; 126.61; 127.16; 127.77; 128.39; 129.48; 130.58; 135.11; 140.08; 147.19; 162.90, 165.00 ppm (Supplementary Materials: Spectrum 23.). Anal. C 58.70, H 4.00, N 12.84% calcd for C_16_H_13_N_3_O_3_S (327.36), C 58.20, H 3.65, N 12.80%

(*E*)-4-(5-(4-chlorostyryl)-1,3,4-oxadiazol-2-yl)benzenesulfonamide (**48**). 

Using 4-chlorobenzaldehyde (0.13 g) and crystalizing the crude product from glacial acetic acid, the title compound was obtained. Yield 0.07 g (25%) as a white powder: mp 296 °C decomp.; IR (KBr) ν_max_ 3304 (NH); 3053 (C_Ar_-H), 1641,1582, 1522, 1484 (C=C, C=N); 1341, 1160 (SO_2_) cm^−1^; ^1^H NMR (DMSO-*d_6_*, 500 MHz) δ 7.47 (d, *J* = 16.1, 1H, C=CH); 7.53 (d, 2H, H-3,5 Cl-phenyl); 7.61 (s, 2H, NH_2_); 7.81 (d,, *J* = 16.6 Hz 1H, C=CH); 7.86 (d, 2H, H-2′,6′); 8.06 (d, 2H, H-2,6); 8.29 (d, 2H, H-3,5) ppm. Anal. C 53.11, H 3.34, N 11.61% calcd for C_16_H_12_ClN_3_O_3_S (361.80), C 53.04, H 3.28, N 11.60%

(*E*)-4-(5-(4-Cyanostyryl)-1,3,4-oxadiazol-2-yl)benzenesulfonamide (**49**). 

Using 4-cyanobenzaldehyde (0.13 g) and crystalizing the crude product from glacial acetic acid, the *E*/*Z* isomers mixture was obtained. Then, it was refluxed in 20 mL of glacial acetic acid for 24 h. Next, the acid was evaporated under reduced pressure and 5ml of ethanol was added, stirred and the obtained precipitate was filtered off, giving pure *E* isomer of the title compound. Yield 0.12 g (45%) as a white powder: mp 270-278 °C decomp.; IR (KBr) ν_max_ 3296, 3191 (NH); 3093 (C_Ar_-H), 2222 (C≡N); 1644, 1604, 1530, 1480 (C=C, C=N); 1345, 1165 (SO_2_) cm^−1^; ^1^H NMR (DMSO-*d_6_*, 500 MHz) δ 7.59 (s, 2H, NH_2_); 7.64 (d, *J* = 16.6, 1H, C=CH); 7.87 (d, *J* = 16.6, 1H, C=CH); 7.93 (d, 2H, H-2′,6′); 8.01 (d, 2H, H-3′,5′); 8.07 (d, 2H, H-2,6); 8.29 (d, 2H, H-3,5) ppm. Anal. C 57.95, H 3.43, N 15.90% calcd for C_17_H_12_N_4_O_3_S (352.37), C 57.47, H 3.60, N 15.64%

(*E*)-4-(5-(4-(Trifluoromethyl)styryl)-1,3,4-oxadiazol-2-yl)benzenesulfonamide (**50**). 

Using 4-(trifluoromethyl)benzaldehyde (0.16 g) and crystalizing the crude product from glacial acetic acid, the title compound was obtained. Yield 0.18 g (61%) as white plates: mp 276-278 °C decomp.; IR (KBr) ν_max_ 3338, 3156 (NH); 3072 (C_Ar_-H), 1634, 1580, 1554, 1533, 1510 (C=C, C=N); 1333, 1160 (SO_2_) cm^−1^; ^1^H NMR (DMSO-*d_6_*, 500 MHz) δ 7.61-7.64 (m, 3H, C=CH, NH_2_); 7.83 (d, *J* = 8.3, 2H, H-3′,5′); 7.91 (d, *J* = 16.6, 1H, C=CH); 8.04-8.07 (m, 4H, H-2′,6′, H-2,6); 8.30 (d, *J* = 8.3, 2H, H-3,5) ppm (Supplementary Materials: Spectrum 24.). ^13^C NMR (DMSO-*d_6_*, 125 MHz) δ: 113.14; 126.27; 126.30; 126.52; 127.17; 127.84; 129.00; 138.27; 139.12; 147.30; 163.16; 164.65 ppm (Supplementary Materials: Spectrum 25.). Anal. C 51.65, H 3.06, N 10.63% calcd for C_17_H_12_F_3_N_3_O_3_S (395.36), C 51.33, H 2.92, N 10.52%

#### 3.1.8. General Procedure for the Preparation of (*E*)-2-(2,4-Dichlorophenyl)-5-(2-Arylvinyl)-1,3,4-Oxadiazols **51**–**54**

Triphenylphosphonium chloride **46** (0.52 g, 1 mmol) and the appropriate aldehyde (1.2 mmol) in 20 mL of dry dichloromethane were put under nitrogen atmosphere and a solution of *N*,*N*-diisopropylethylamine (0.19 g, 1.5 mmol) in 5 mL of dichloromethane was added. The reaction mixture was stirred at room temperature for 24 h. Next, the solvent was evaporated under reduced pressure, ethanol (5 mL) was added and the suspension was stirred vigorously at room temperature for 1 h. The precipitate was filtered off and the crude product was purified as follows:

(*E*)-2-(2,4-Dichlorophenyl)-5-styryl-1,3,4-oxadiazole (**51**) 

Using benzaldehyde (0.13 g) and crystalizing the crude product from ethanol, the title compound was obtained. Yield 0.24 g (75%) as white needles: mp 154-155 °C; IR (KBr) ν_max_ 3087, 3066, 3028 (C_Ar_-H), 1647, 1591, 1525, 1456 (C=C, C=N); 1345, 1165 (SO_2_) cm^−1^; ^1^H NMR (CDCl_3_, 500 MHz) δ: 7.15 (d, *J* = 16.1, 1H, C=CH); 7.43-7.47 (m, 4H, H-5, H-3′,4′,5′); 7.61–7.63 (m, 3H, H-3, H-2′,6′); 7.67 (d, *J* = 16.1, 1H,C=CH); 8.07 (d, *J* = 8.8, 1H, H-6) ppm (Supplementary Materials: Spectrum 26.). ^13^C NMR (CDCl_3_, 125 MHz) δ: 109,65; 121,66; 127,64; 127,67; 129,07; 130,20; 131,26; 131,87; 133,83; 134,61; 138,11; 139,79; 161,74; 164,81 ppm (Supplementary Materials: Spectrum 27.). Anal. C 60.59, H 3.18, N 8.83% calcd for C_16_H_10_Cl_2_N_2_O (317.17), C 60.28, H 2.99, N 8.82%

(*E*)-2-(4-Chlorostyryl)-5-(2,4-dichlorophenyl)-1,3,4-oxadiazole (**52**) 

Using 4-chlorobenzaldehyde (0.17 g) and crystalizing the crude product from glacial acetic acid, the title compound was obtained. Yield 0.19 g (82%) as a white fluffy powder: mp 187-189 °C; IR (KBr) ν_max_ 3093, 3060, 3026 (C_Ar_-H); 1648, 1595, 1523, 1486 (C=C, C=N) cm^−1^; ^1^H NMR (CDCl_3_, 500 MHz) δ: 7.11 (d, *J* = 16.6, 1H, C=CH); 7.42-7.45 (m, 3H, H-5, H-2′,6′); 7.54 (d, 2H, H-3′,5′); 7.61 (d, *J* = 14.7, 1H,C=CH); 7.62 (s, 1H, H-3); 8.07 (d, 1H, H-6) ppm. Anal. C 54.65, H 2.58, N 7.97% calcd for C_16_H_9_Cl_3_N_2_O (351.61), C 54.29, H 2.40, N 7.92%

(*E*)-4-(2-(5-(2,4-Dichlorophenyl)-1,3,4-oxadiazol-2-yl)vinyl)benzonitrile (**53**) 

Using 4-cyanobenzaldehyde (0.16 g) and crystalizing the crude product from glacial acetic acid, the title compound was obtained. Yield 0.19 g (57%) as a white fluffy powder: mp 226-236 °C; IR (KBr) ν_max_ 3048, 3021 (C_Ar_-H); 2225 (C≡N); 1643, 1592, 1567, 1519, 1455 (C=C, C=N) cm^−1^; ^1^H NMR (CDCl_3_, 500 MHz) δ: 7.24 (d, *J* = 16.6, 1H, C=CH); 7.46 (dd, 1H, H-5); 7.63-7.67 (m, 2H, H-3, C=CH); 7.70 (d, 2H,H-2′,6′); 7.75 (d, 2H, H-3′,5′); 8.07 (d, 1H, H-6)ppm. Anal. C 59.67, H 2.65, N 12.28% calcd for C_17_H_9_Cl_2_F_3_N_3_O (342.18), C 59.48, H 2.54, N 12.10%

(*E*)-2-(2,4-Dichlorophenyl)-5-(4-(trifluoromethyl)styryl)-1,3,4-oxadiazole (**54**) 

Using 4-(trifluoromethyl)benzaldehyde (0.21 g) and crystalizing the crude product from ethanol, the title compound was obtained. Yield 0.32 g (82%) as white needles: mp 168-170 °C; IR (KBr) ν_max_ 3063, 3047 (C_Ar_-H), 1645, 1592, 1563,1456 (C=C, C=N) cm^−1^; ^1^H NMR (DMSO-d_6_, 500 MHz) δ: 7.61 (d, *J* = 16.1, 1H, C=CH); 7.71 (dd, *J* = 8.6, *J* = 1.7, 1H, H-5); 7.80-7.84 (m, 3H, H-3′,5′, C=CH); 7.94 (d, *J* = 2.0, 1H,H-3); 8.04 (d, *J* = 8.3, 2H, H-2′,6′); 8.14 (d, *J* = 8.7, 1H, H-6) ppm (Supplementary Materials: Spectrum 28.). ^13^C NMR (DMSO-*d_6_*, 125 MHz) δ: 113.08; 121.97; 126.17; 126.20 128.62; 129.03; 131.25; 132.94; 133.53; 137.73; 138.37; 139.08; 161.58; 164.54 ppm (Supplementary Materials: Spectrum 29.). Anal. C 53.01, H 2.36, N 7.27% calcd for C_17_H_9_Cl_2_F_3_N_2_O (385.17), C 52.78, H 2.10, N 7.12%

### 3.2. Cell Culture and Cell Viability Assay

All chemicals, if not stated otherwise, were obtained from Sigma-Aldrich (St. Louis, MO, USA). The MCF-7, HeLa and HaCaT cell lines were purchased from Cell Lines Services (Eppelheim, Germany), the HCT-116 cell line were obtained from ATCC (ATCC-No: CCL-247). Cells were cultured in Dulbecco’s modified Eagle’s medium (DMEM) supplemented with 10% fetal bovine serum, 2 mM glutamine, 100 units/mL penicillin, and 100 μg/mL streptomycin. Cultures were maintained in a humidified atmosphere with 5% CO_2_ at 37 °C in an incubator (HeraCell, Heraeus, Langenselbold, Germany). Cell viability was determined using the MTT [3-(4,5-dimethylthiazol-2-yl)-2,5-diphenyl-tetrazolium bromide] assay. Stock solutions of the studied compounds were obtained in 100% DMSO. Working solutions were prepared by diluting the stock solutions with DMEM medium, the final concentration of DMSO did not exceed 0.5% in the treated samples. Cells were seeded in 96-well plates at a density of 5 × 10^3^ cells/well and treated for 72 h with the examined compounds in the concentration range 1–100 μM (1, 10, 25, 50 and 100 μM). Following treatment, MTT (0.5 mg/mL) was added to the medium and cells were further incubated for 2 h at 37 °C. Cells were lysed with DMSO and the absorbance of the formazan solution was measured at 550 nm with a plate reader Victor 1420 multilabel counter, (PerkinElmer, Inc. Waltham, MA USA). The optical density of the formazan solution was measured at 550 nm with a plate reader (1420 multilabel counter, Victor). The experiment was performed in triplicate. Values are expressed as the mean ± SD of at least three independent experiments

### 3.3. QSAR Studies

Two dimensional structures of compounds were draw using MarvinSketch (MarvinSketch 18.24.0 2018, ChemAxon, Budapest, Hungary) and were exported to the database created in Molecular Operating Environment (MOE, 2019.01) (Chemical Computing Group, Montreal, QC, Canada). Structures were pre-optimized to three-dimensions with the molecular mechanic method using the MMFF94 forcefield. Next, to search for the lowest energy conformation, pre-optimized structures were subjected to a conformational search using the LowModeMD method implemented in MOE. For every compound, 50 lowest energy conformations were treated as the starting point to perform geometry optimization with semi empirical PM6 method implemented in MOPAC2016 (MOPAC2016, Version 18.305W, Stewart Computational Chemistry, Colorado Springs, CO, USA.). If, after PM6 calculation, the total energy of two or more conformers was equal, the selection of the lowest energy one was based on a single-point energy calculated with Hartree-fock method and STO-3G* basis set using GAMESS software [27] (GAMESS version 14 FEB 2018 (R1), Iowa State University Quantum Chemistry Group, Ames, IA, USA). For structures optimized in this way, 293 two- and three-dimensional molecular descriptors were calculated using MOE. The descriptors matrix was then subjected to standardization procedure according to the formula:

z = (x − µ)/σ

where x is the descriptor value; µ is the average; and σ is the standard deviation.

Cluster analysis, as well as multiple linear regression, along with forward stepwise algorithm and model validation, was performed in Statistica (Statistica version 13, TIBCO Software Inc Palo Alto, CA, USA) software [28] on a personal computer. In cluster analysis, the distances between all samples were computed using the Euclidean distance, and Ward’s method of clustering was employed.

## 4. Conclusions

A new series of (*E*)-2-R1-4-chloro-5-[5-(2-Ar-vinyl)-1,3,4-oxadiazol-2-yl]benzenesulfonamides **7**–**36**, (*E*)-4-[5-styryl1,3,4-oxadiazol-2-yl]benzenesulfonamides **47**–**50** and (*E*)-2-(2,4-Dichlorophenyl)-5-(2-arylvinyl)-1,3,4-oxadiazols **51**–**55** have been synthesized using Wittig reaction. The molecular structures of these novel compounds were confirmed by elemental analyses and spectroscopic methods: NMR and IR. The newly synthesized compounds were tested for their in vitro cytotoxic activity against the MCF-7, HCT-116 and HeLa cancer cells and on the noncancerous keratinocyte cell line HaCaT.

Compound **31** proved to be the most active among all tested derivatives, showing IC_50_ values for tumor cell lines in the range 0.5-4.5 μM. Analysis of the structure–activity relationship showed that the activity of compounds substituted with a chlorine atom in position 2 of benzenesulfonamide scaffold (R^1^ = Cl) is much more diverse and more dependent on the structure of Ar substituent than for compounds substituted with benzylthio in position 2 of benzenesulfonamide scaffold (R^1^ = SBn) which, however, show lower average activity. Analyzing the influence of different benzenesulfonamide scaffold, we have observed a more favorable effect of the sulfonamide group in the *meta* position to the oxadiazole ring, than in the *para* position, or for the total absence of sulfonamide moiety.

Hierarchical cluster analysis of compounds **7**–**36** with the use of 293 quantitative molecular descriptors obtained by Molecular Operating Environment (MOE) software. Analysis showed a reasonable division of compounds into two clusters (R^1^ = Cl and R^1^ = SBn), and showed that the most active compound **31** exhibits outlying molecular properties.

Using the MLR method, QSAR equations **E1**–**E6** with good predicting properties were obtained, useful for assessing the activity of new derivatives during further optimization steps. The molecular descriptors used in the QSAR Equations indicate the potentially beneficial effect of increasing the proportion of the surface of negatively charged atoms and increasing the number of atoms with greater polarity or molar refraction.

## Figures and Tables

**Table 1:**
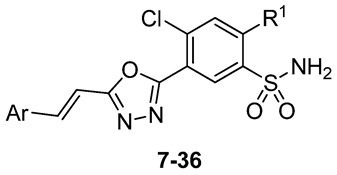
Cytotoxicity of compounds **7**–**36** towards human cancer cell lines.

Compd	Ar	R^1^	IC_50_ [µM] ^a^			
			HCT-116	MCF-7	HeLa	HaCaT
**7**	Ph	Cl	86 ± 3	165 ± 5	455 ±14	n.t.
**8**[18]		SCH_2_Ph	18.1 ± 0.2	16.3 ± 0.2	13.8 ± 0.1	20 ± 1
**9**	3-Cl-Ph	Cl	24 ± 1	150 ± 10	19 ± 1	98 ± 2
**10**[18]		SCH_2_Ph	12.0 ± 0.2	11.3 ± 0.4	16.1 ± 0.1	16 ± 0.5
**11**	4-Cl-Ph	Cl	22 ± 1	28 ± 1	42 ±1	51 ± 3
**12**[18]		SCH_2_Ph	11.7 ± 0.4	10.7 ± 0.3	40 ± 1	n.t.
**13**	2,6-diCl-Ph	Cl	16 ± 1	92 ± 5	25 ± 1	95 ± 2
**14**[18]		SCH_2_Ph	31.8 ± 0.3	29.4 ± 0.3	12.5 ± 0.1	160 ± 2
**15**	4-Br-Ph	Cl	34 ± 1	41 ± 1	34 ± 2	55 ± 2
**16**[18]		SCH_2_Ph	15.0 ± 0.3	12.9 ± 0.4	29.2 ± 0.8	31 ± 1
**17**	4-OH-Ph	Cl	49 ± 1	73 ± 3	99 ± 1	100 ± 5
**18**		SCH_2_Ph	18 ± 1	59 ± 3	18.2 ± 0.5	25.6 ± 0.5
**19**	4-CN-Ph	Cl	13.7 ± 0.5	10.0 ± 0.3	117 ± 6	102 ± 3
**20**		SCH_2_Ph	10.3 ± 0.2	8.9 ± 0.5	12.2 ± 0.5	20 ± 1
**21**	4-CF_3_-Ph	Cl	18 ± 1	75 ± 2	90 ± 2	95 ± 4
**22**		SCH_2_Ph	15.2 ± 0.1	30 ± 1	33.4 ± 0.5	33 ± 1
**23**	Pyridin-4-yl	Cl	255 ± 13	640 ± 13	740 ± 37	n.t.
**24**		SCH_2_Ph	31±1	225 ± 11	135 ± 8	150 ± 8
**25**	Pyridin-3-yl	Cl	220 ± 11	760 ± 24	570 ±17	n.t.
**26**		SCH_2_Ph	85 ± 5	350 ± 17	135 ± 4	n.t.
**27**	Pyridin-2-yl	Cl	117 ± 4	110 ± 2	170 ± 5	n.t.
**28**		SCH_2_Ph	25 ± 1	115 ± 3	130 ± 6	140 ± 10
**29**	Thiophen-2-yl	Cl	108 ± 4	175 ± 12	245 ±12	n.t.
**30**		SCH_2_Ph	32 ± 2	66 ± 2	87 ± 3	85 ± 4
**31**	5-Nitrothiophen-2-yl	Cl	0.52 ± 0.01	4.0 ± 0.1	4.5 ±0.3	8.1 ± 0.5
**32**		SCH_2_Ph	17 ± 1	55 ± 3	58 ± 2	57 ± 1
**33**	Furan-2-yl	Cl	33 ± 1	170 ± 9	210 ± 8	200 ± 12
**34**		SCH_2_Ph	27.8 ± 0.3	62 ± 1	85 ± 3	91 ± 3
**35**	5-Nitrofuran-2-yl	SCH_2_Ph	4.1 ± 0.2	150 ± 8	69 ± 3	87 ± 4
**36**	Pyrrol-2-yl	Cl	39 ± 1	40 ± 1	73 ± 3	98 ± 4
**Cisplatin**			3.8 ± 0.2	3.1 ± 0.1	2.2 ±0.1	n.t.

^a^ Analysis was performed using the MTT assay after 72 h of incubation. Values are expressed as the mean ± SD of at least three independent experiments; n.t.—not tested.

**Table 2:**
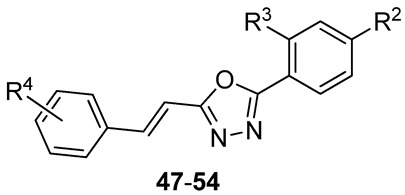
Cytotoxicity of compounds **47**–**54**.

Compd	R^2^	R^3^	R^4^	IC_50_ [µM] ^a^			
				HCT-116	MCF-7	HeLa	HaCaT
**47**	SO_2_NH_2_	H	H	185 ± 11	168 ± 8	130 ± 7	n.t.
**48**	SO_2_NH_2_	H	4-Cl	105 ± 5	76 ± 4	25 ± 3	95 ± 4
**49**	SO_2_NH_2_	H	4-CN	32 ± 2	75 ± 4	99 ± 3	98 ± 4
**50**	SO_2_NH_2_	H	4-CF_3_	155 ± 7	280 ± 15	520 ± 25	n.t.
**51**	Cl	Cl	H	92 ± 2	125 ± 6	190 ± 10	n.t.
**52**	Cl	Cl	4-Cl	64 ± 3	110 ± 6	215 ± 6	n.t.
**53**	Cl	Cl	4-CN	106 ± 3	142 ± 6	225 ± 11	n.t.
**54**	Cl	Cl	4-CF_3_	98 ± 1	102 ± 5	104 ± 3	n.t.

^a^ Analysis was performed using the MTT assay after 72 h of incubation. Values are expressed as the mean ± SD of at least three independent experiments; n.t.—not tested.

**Table 3:** Basic statistical parameters for anticancer activity (IC_50_ [µM]) of two groups of compounds R^1^ = Cl and R^1^ = SBn.

		IC_50_ [µM] R^1^ = Cl			IC_50_ [µM] R^1^ = SBn			
Cell Line	Min	Max	Average	Standard Deviation	Min	Max	Average	Standard Deviation
HCT-116	0.5	255	69	76.8	4	85	24	19.0
MCF-7	4	760	169	224.3	9	350	80	96.3
HeLa	4.5	740	193	222.9	12	135	58	46.3

**Table 4:** The QSAR equations **E1**–**E6** and their statistical parameters of the two groups of derivatives (R^1^ = Cl and R^1^ = S Bn) against MCF-7 and HCT-116 cell lines.

Cell Line	R^1^ = Cl		R^1^ = SBn	
	Equation	Statistics	Equation	Statistics
HCT-116	“**E1**” pIC_50_= 4.4571 + 0.5082(SMR_VSA2) − 0.2849(logS) + 0.1829(SMR_VSA3) ± 0.1579	F = 75.21 p < 10^−6^ R^2^ = 0.94 Q^2^ = 0.92	“**E2**” pIC_50_ = 4.7247 + 0.2994(FCASA−) + 0.2222(vsurf_DD23) − 0.1596(SMR_VSA1) ± 0.1415	F = 17.19 p = 2 × 10^−4^ R^2^ = 0.78 Q^2^ = 0.72
MCF-7	“**E3**” pIC_50_ = 4.0959 + 0.5488(vsurf_Wp2) + 0.2418(vsa_other) − 0.2190(vsurf_HB1) ± 0.2263	F = 29.43 p = 2 × 10^−5^ R^2^ = 0.86 Q^2^ = 0.83	“**E4**” pIC_50_ = 4.3633 + 0.5904(PEOE_VSA_FNEG) + 0.2738(vsurf_ID8) − 0.2166(vsurf_IW2) ± 0.1365	F = 60.43 p < 10^−6^ R^2^ = 0.93 Q^2^ = 0.80
HeLa	“**E5**” pIC_50_ = 4.0280 + 0.4800(FASA−) + 0.3253(SMR_VSA2) − 0.2252(E_ele) ± 0.2094	F = 35.47 p = 6 × 10^−6^ R^2^ = 0.88 Q^2^ = 0.81	“**E6**” pIC_50_ = 4.3872 + 0.4976(chi1v_C) + 0.2609(BCUT_SMR_2) − 0.1388(pmi) ± 0.1258	F = 42.30 p = 3 × 10^−6^ R^2^ = 0.90 Q^2^ = 0.85

pIC_50_ = −log_10_(IC_50_); F—Fisher’s test, p—*p*-value for Fisher’s test; R^2^—squared correlation coefficient for training set; Q^2^—5-fold cross-validation squared correlation coefficient.

## Supplementary Materials

Supplementary materials can be found at https://www.mdpi.com/1422-0067/21/6/2235/s1.
