# Supplementary material for: Synthesis, Anticancer Evaluation and Structure-Activity Analysis of Novel (E)- 5-(2-Arylvinyl)-1,3,4-oxadiazol-2-yl)benzenesulfonamides
Source: Int J Mol Sci. 2020 Mar 23;21(6):2235. doi: 10.3390/ijms21062235 (PMC7139731; doi:10.3390/ijms21062235)

Supplementary Materials

Synthesis, Anticancer Evaluation and Structure-Activity Analysis of Novel (E)- 5-(2-Arylvinyl)-1,3,4-oxadiazol-2-yl)benzenesulfonamides

Krzysztof Szafrański [1]*, Jarosław Sławiński [1], Łukasz Tomorowicz [1] and Anna Kawiak [2]

[1] Department of Organic Chemistry, Medical University of Gdańsk, Al. Gen. J. Hallera 107, 80-416 Gdańsk, Poland; krzysztof.szafranski@gumed.edu.pl (K.S.), jaroslaw.slawinski@gumed.edu.pl (J.S.), lukasz.tomorowicz@gumed.edu.pl (Ł .T)
[2] Department of Biotechnology, Intercollegiate Faculty of Biotechnology, University of Gdańsk and Medical University of Gdańsk, ul. Abrahama 58, 80-307 Gdańsk, Poland; anna.kawiak@biotech.ug.edu.pl (A.K.)
* Correspondence krzysztof.szafranski@gumed.edu.pl; Tel.: +48-58-349-12-77

Table of Contents

Spectrum 1. ^{1}H-NMR of compound **4** (500 MHz, DMSO-d_6). 3
Spectrum 2. ^{1}H-NMR of compound **7** (500 MHz, DMSO-d_6). 4
Spectrum 3. ^{13}C-NMR of compound **7** (125 MHz, DMSO- d_6). 5
Spectrum 4. ^{1}H-NMR of compound **11**(500 MHz, DMSO-d_6). 6
Spectrum 5. ^{13}C-NMR of compound **11** (125 MHz, DMSO- d_6). 7
Spectrum 6. ^{1}H-NMR of compound **13** (500 MHz, DMSO-d_6). 8
Spectrum 7. ^{13}C-NMR of compound **13** (125 MHz, DMSO- d_6). 9
Spectrum 8. ^{1}H-NMR of compound **17** (500 MHz, DMSO-d_6). 10
Spectrum9. ^{13}C-NMR of compound **17** (125 MHz, DMSO- d_6). 11
Spectrum 10. ^{1}H-NMR of compound **27** (500 MHz, DMSO-d_6). 12
Spectrum11. ^{13}C-NMR of compound **27** (125 MHz, DMSO- d_6). 13
Spectrum 12. ^{1}H-NMR of compound **28** (500 MHz, DMSO-d_6). 14
Spectrum13. ^{13}C-NMR of compound **28** (125 MHz, DMSO- d_6). 15
Spectrum 14. ^{1}H-NMR of compound **31** (500 MHz, DMSO-d_6). 16
Spectrum15. ^{1}H-NMR of compound **32** (500 MHz, DMSO- d_6). 17
Spectrum 16. ^{1}H-NMR of compound **35** (500 MHz, DMSO-d_6). 18
Spectrum17. ^{13}C-NMR of compound **35** (125 MHz, DMSO- d_6). 19
Spectrum 18. ^{1}H-NMR of compound **36** (500 MHz, DMSO-d_6). 20
Spectrum 19. ^{13}C-NMR of compound **36** (125 MHz, DMSO- d_6). 21
Spectrum 20. ^{1}H-NMR of compound **43** (500 MHz, DMSO-d_6). 22
Spectrum 21. ^{13}C-NMR of compound **43** (125 MHz, DMSO- d_6). 23

Spectrum 22. ^{1}H-NMR of compound **47** (500 MHz, DMSO-d_6). 24
Spectrum 23. ^{13}C-NMR of compound **47** (125 MHz, DMSO- d_6). 25
Spectrum 24. ^{1}H-NMR of compound **50** (500 MHz, DMSO-d_6). 26
Spectrum 25. ^{13}C-NMR of compound **50** (125 MHz, DMSO- d_6). 27
Spectrum 26. ^{1}H-NMR of compound **51** (500 MHz, $CDCl_3$)... 28
Spectrum 27. ^{13}C-NMR of compound **51** (125 MHz, $CDCl_3$).. 29
Spectrum 28. ^{1}H-NMR of compound **54** (500 MHz, DMSO-d_6). 30
Spectrum 29. ^{13}C-NMR of compound **54** (125 MHz, DMSO- d_6). 31

Spectrum 1. ^{1}H-NMR of compound **4** (500 MHz, DMSO-d_6).

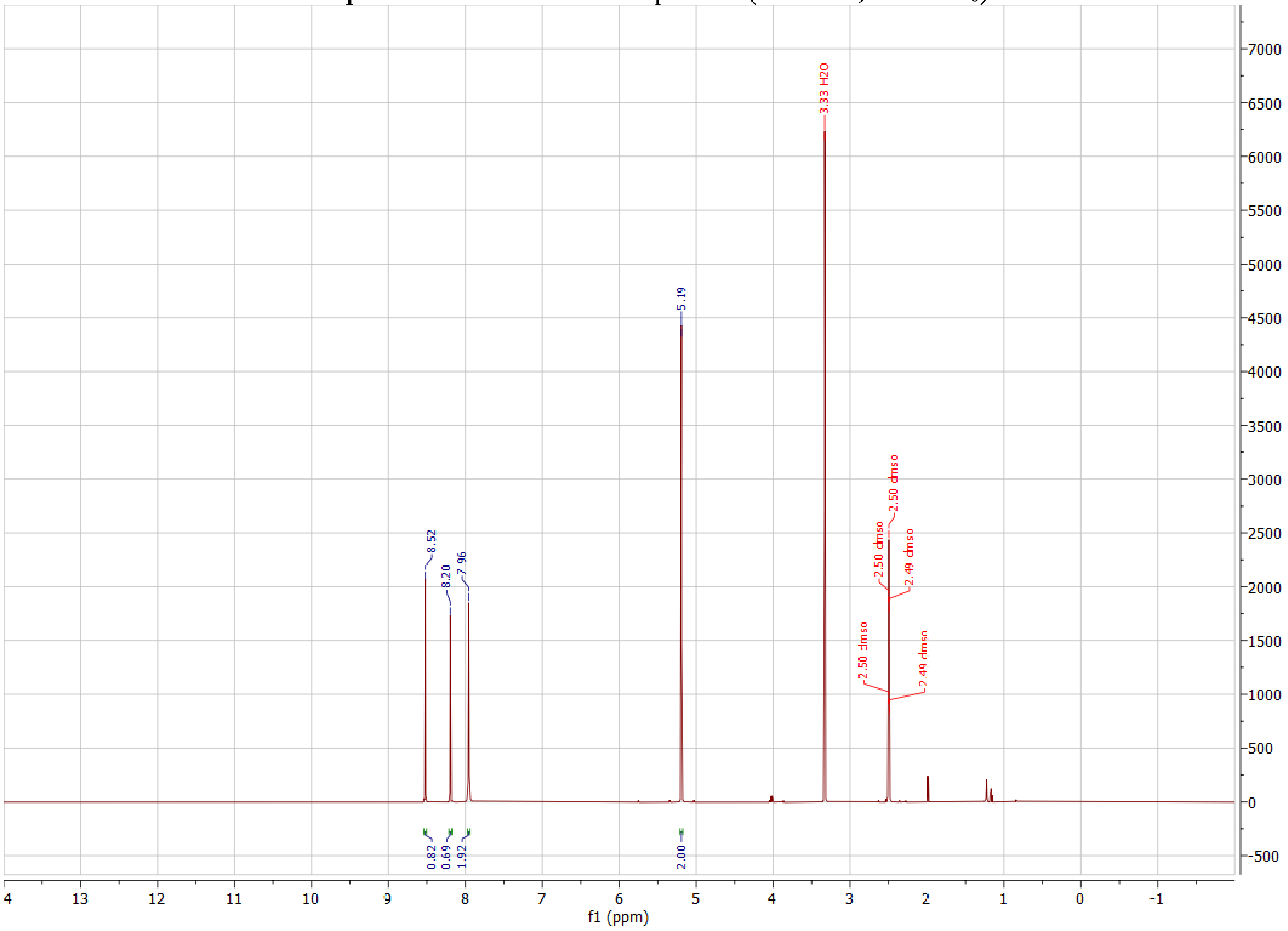

Spectrum 2. ^{1}H-NMR of compound **7** (500 MHz, DMSO-d_6).

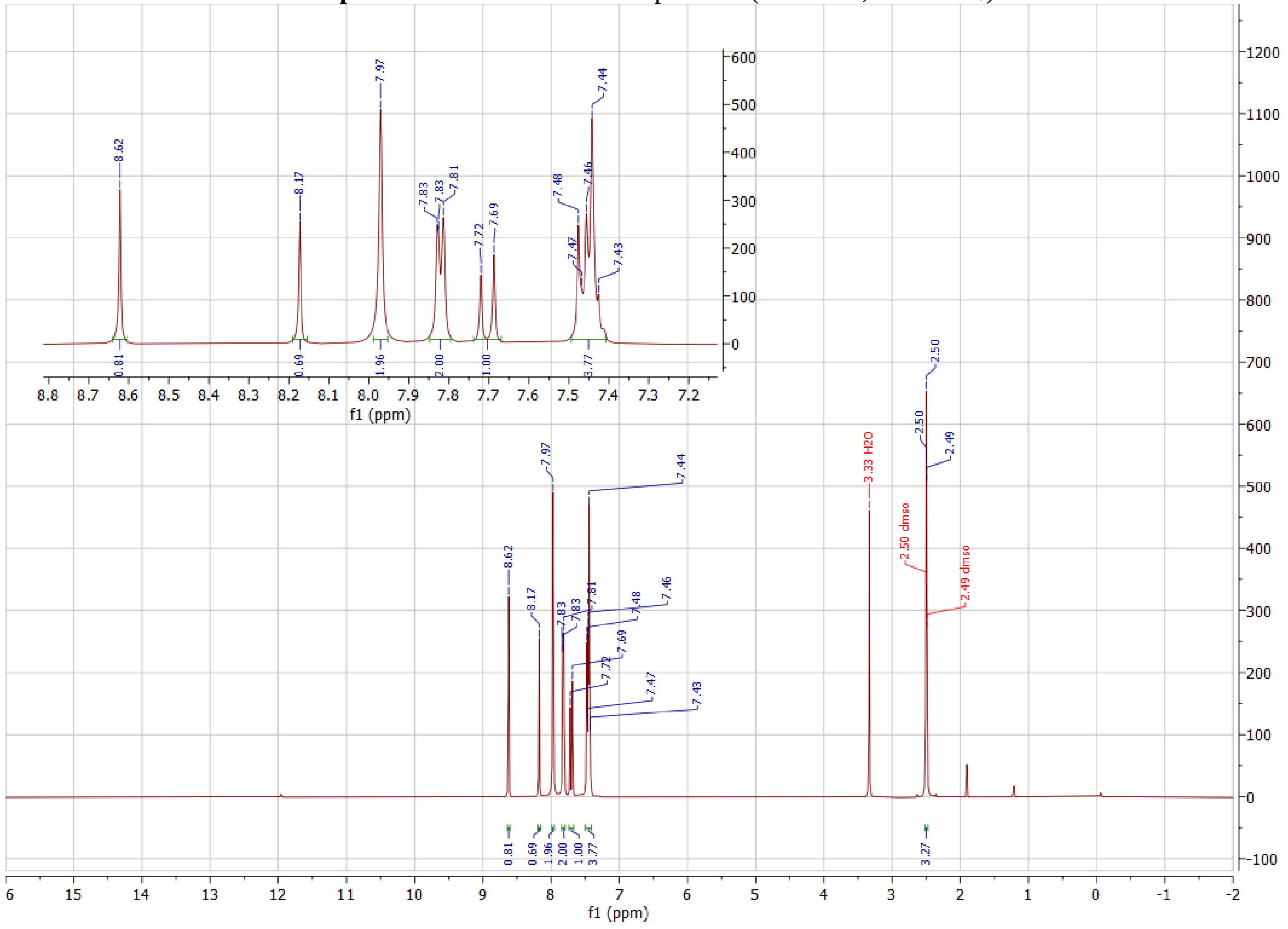

Spectrum 3. ^{13}C-NMR of compound **7** (125 MHz, DMSO- d_6).

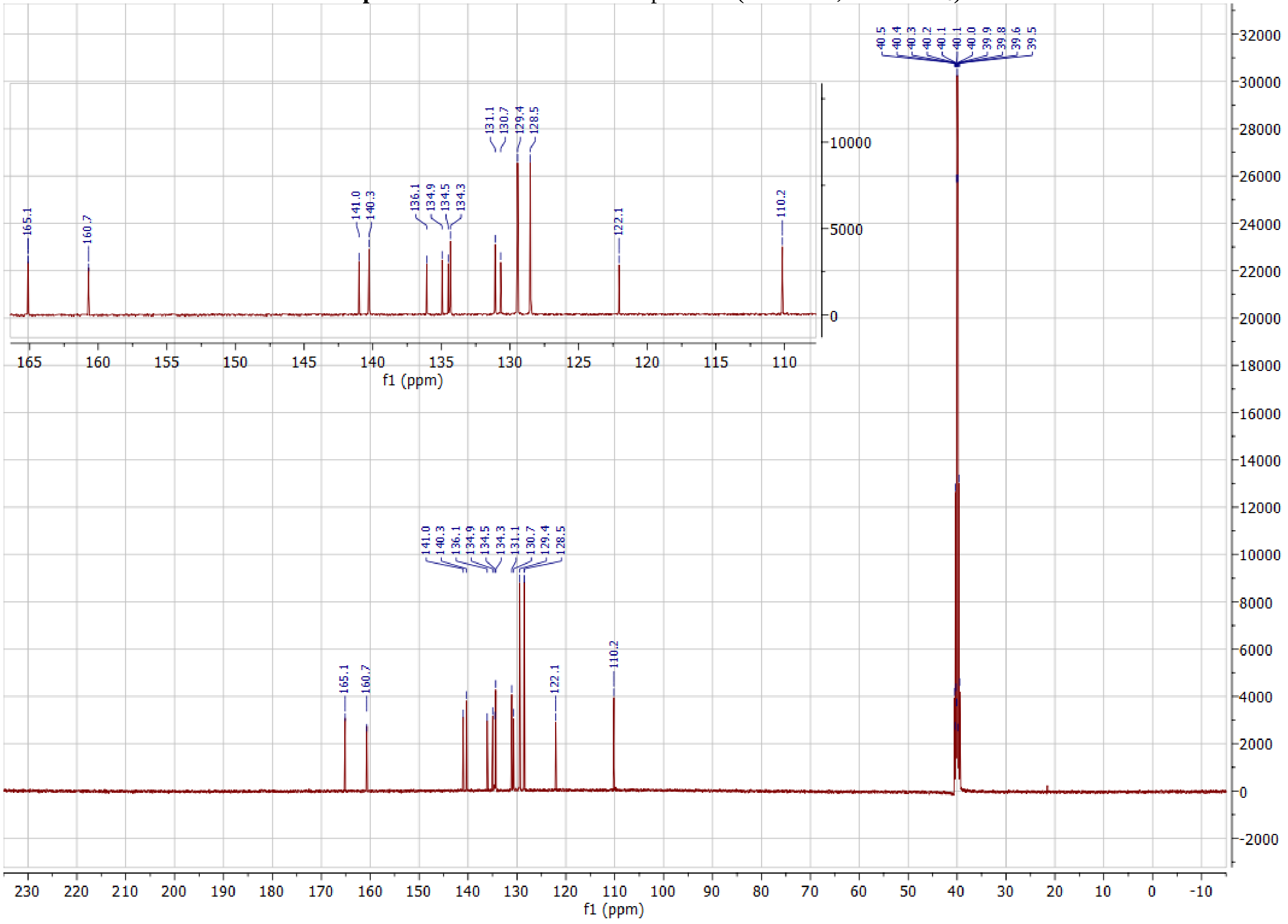

Spectrum 4. ^{1}H-NMR of compound **11**(500 MHz, DMSO-d_6).

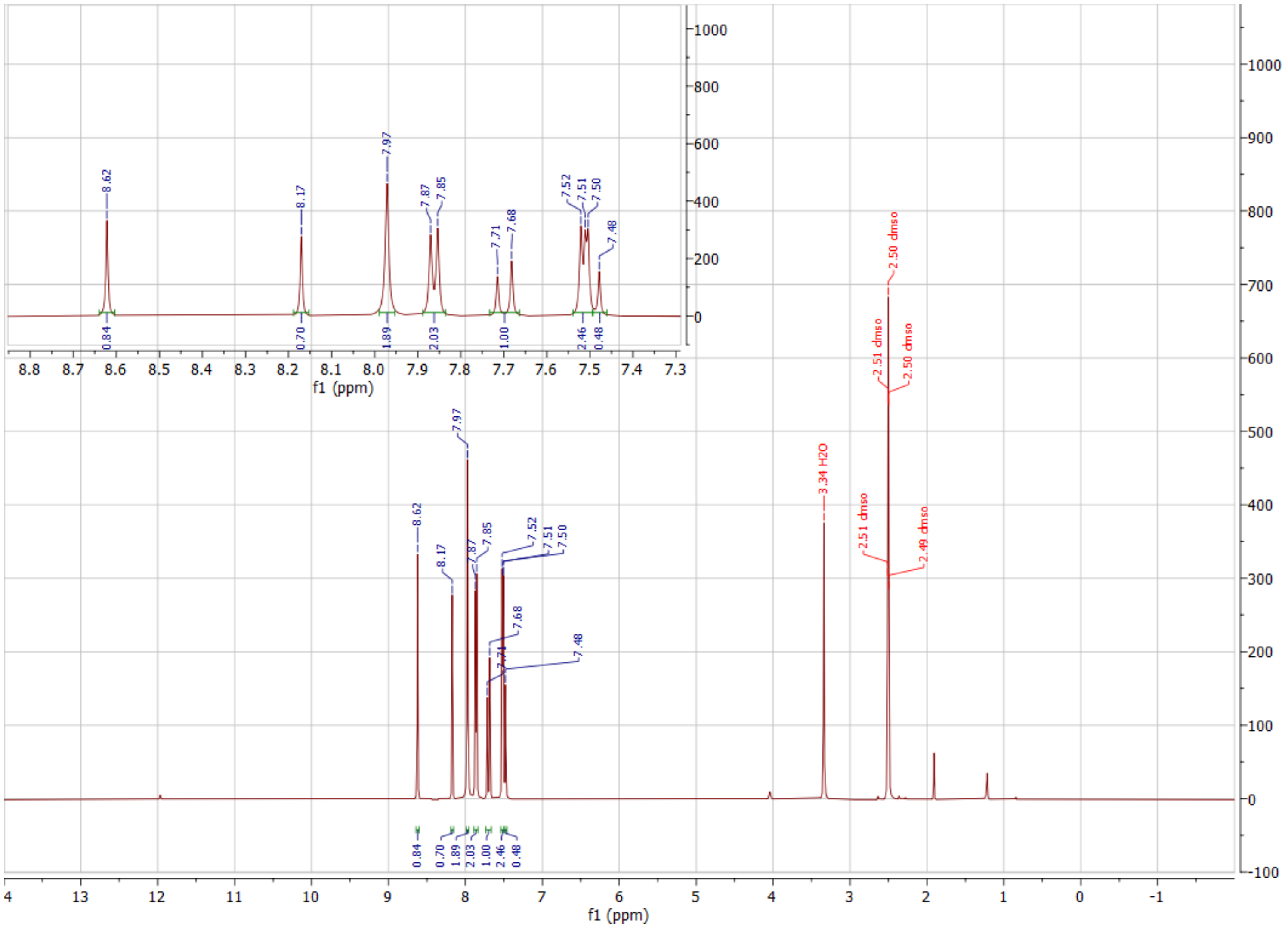

Spectrum 5. ^{13}C-NMR of compound **11** (125 MHz, DMSO- d_6).

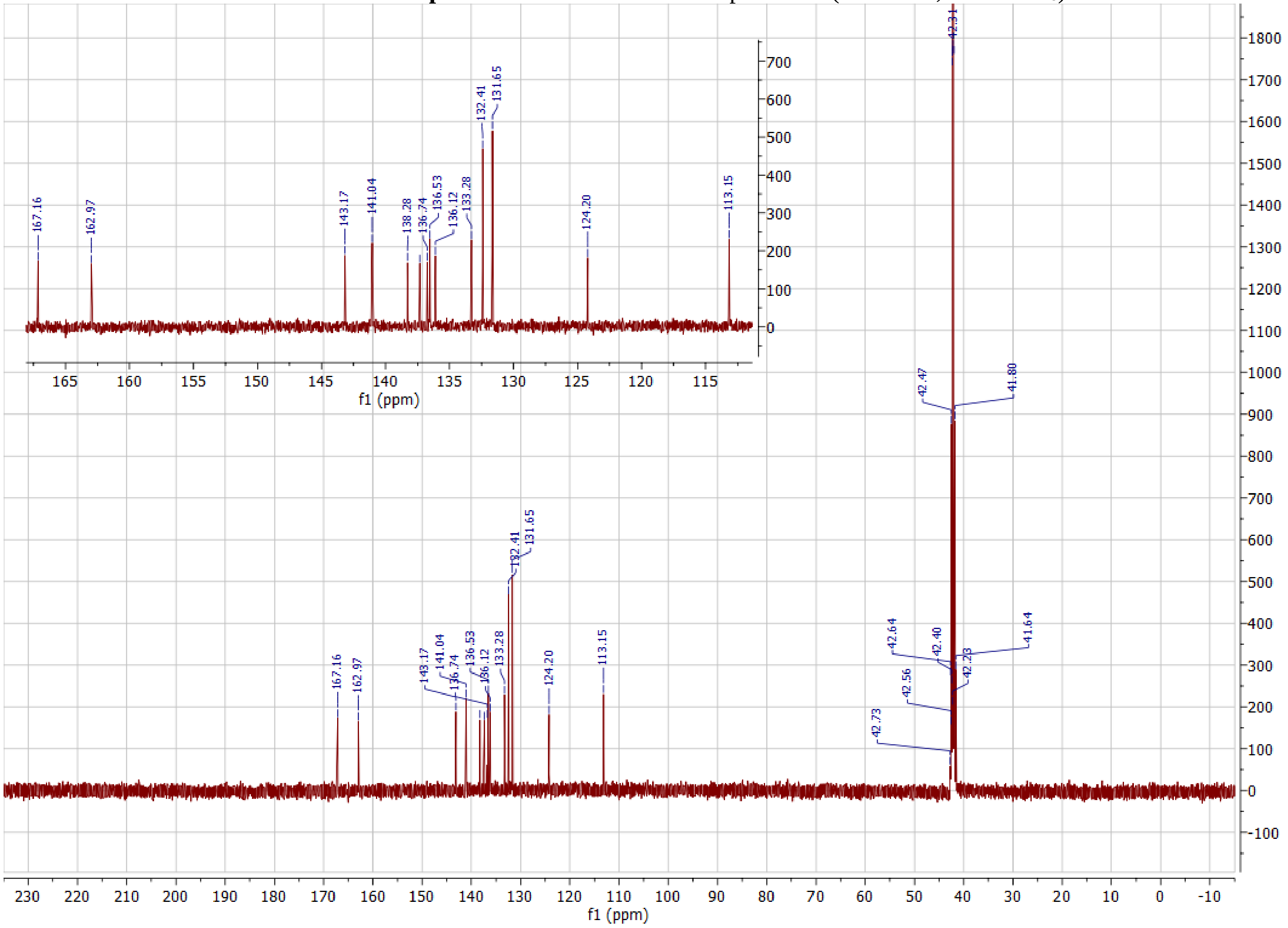

Spectrum 6. ^{1}H-NMR of compound **13** (500 MHz, DMSO-d_6).

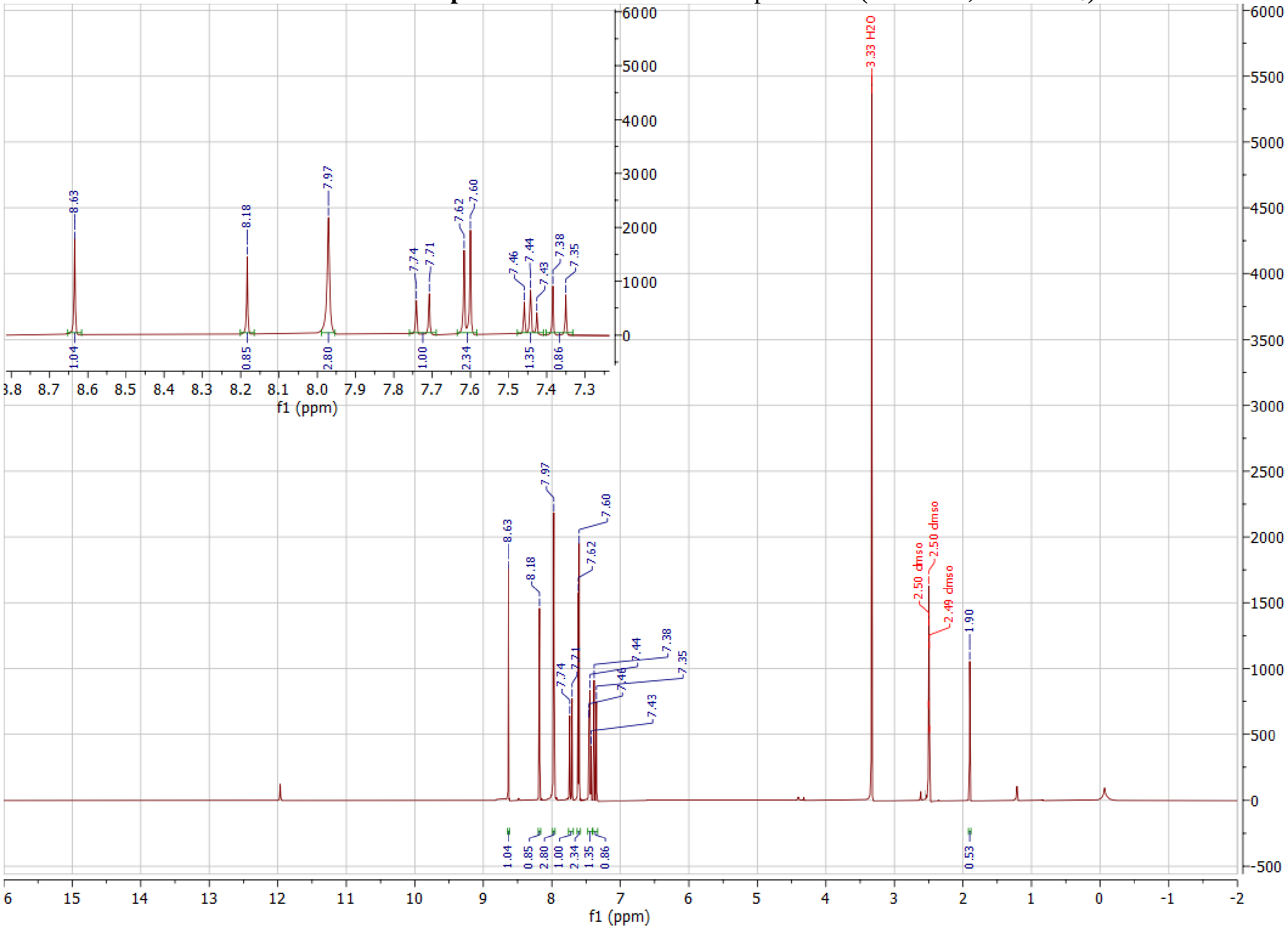

Spectrum 7. ^{13}C-NMR of compound **13** (125 MHz, DMSO- d_6).

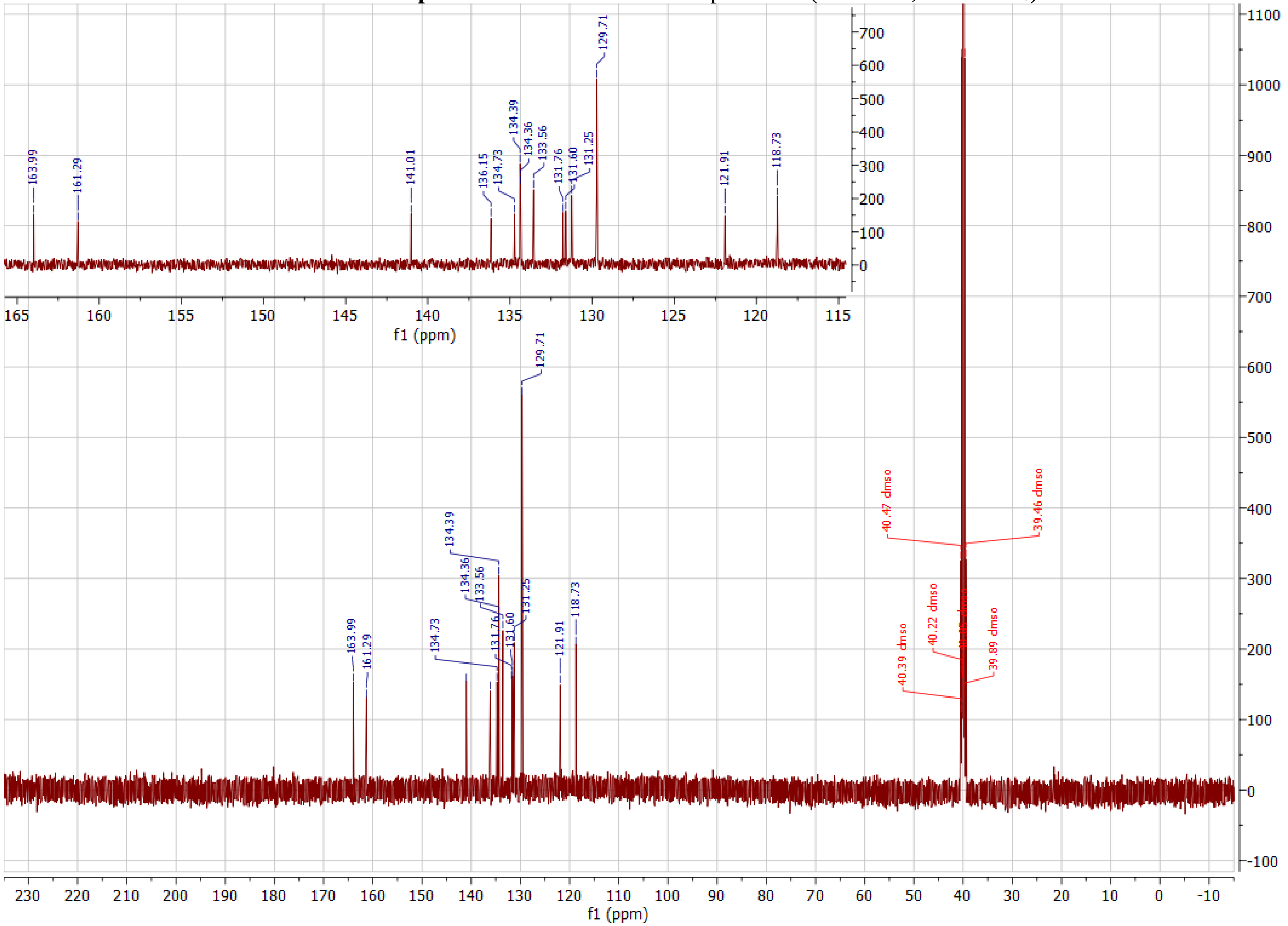

Spectrum 8. ^{1}H-NMR of compound **17** (500 MHz, DMSO-d_6).

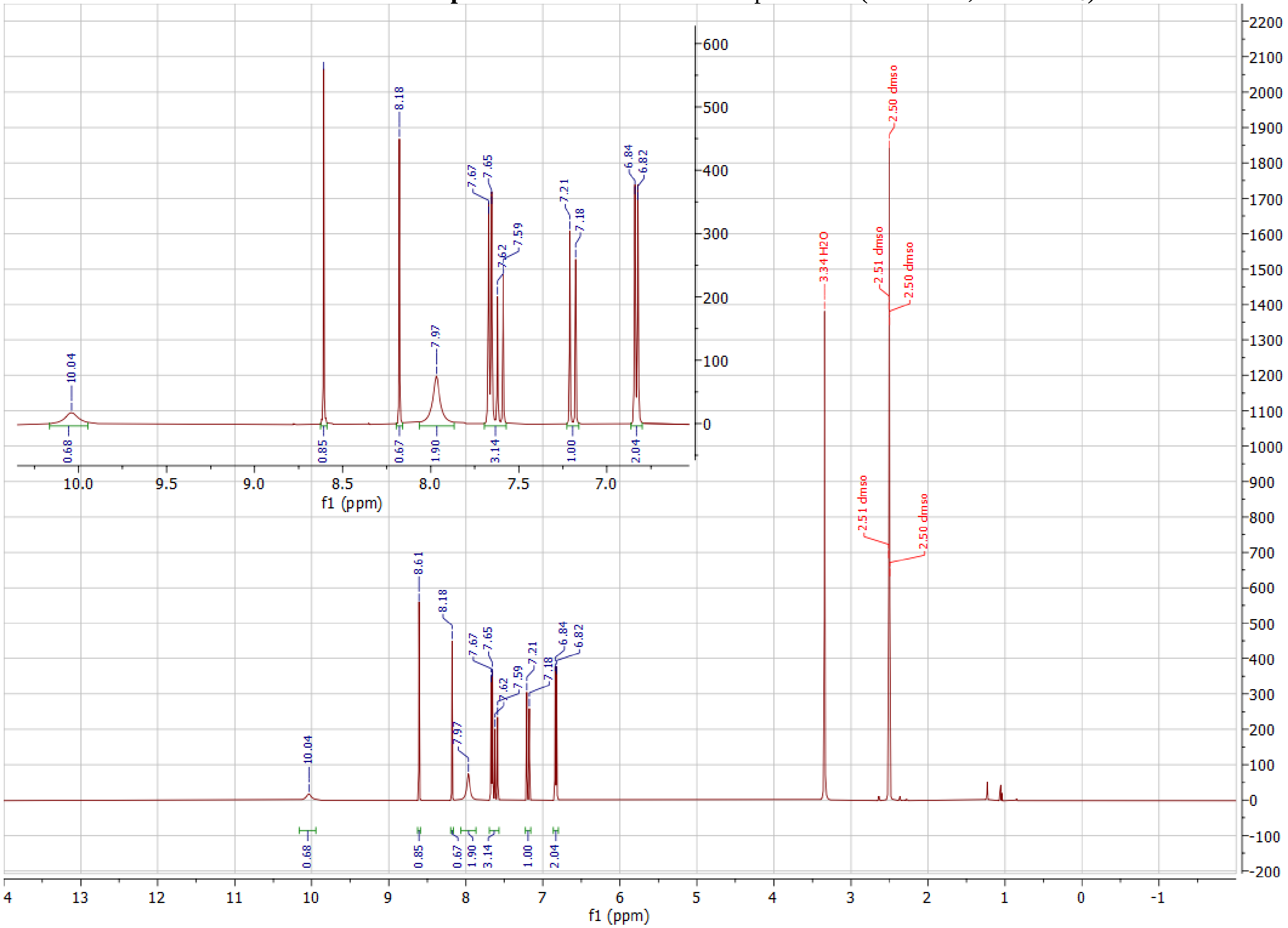

Spectrum9. ^{13}C-NMR of compound **17** (125 MHz, DMSO- d_6).

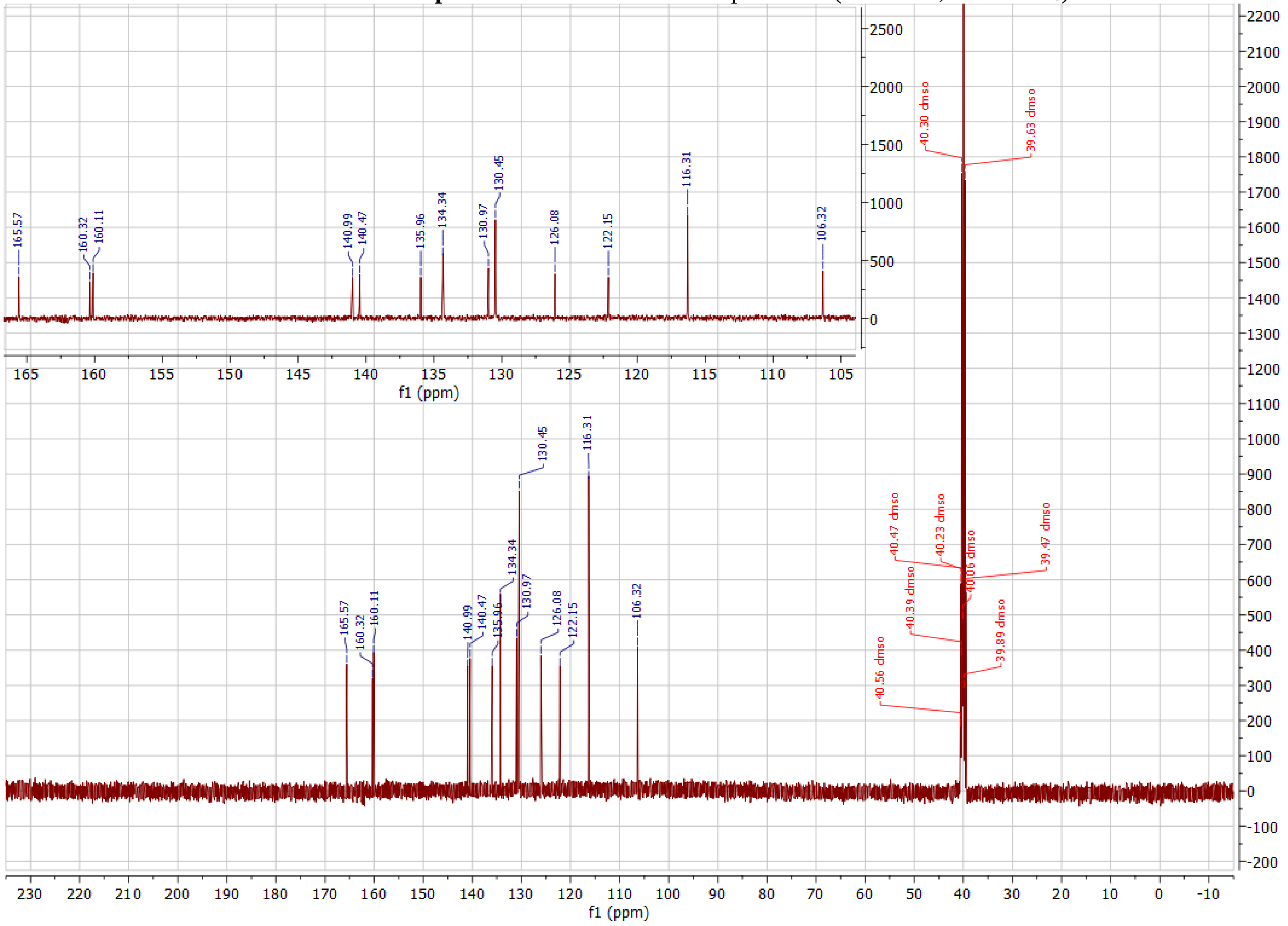

Spectrum 10. ^{1}H-NMR of compound **27** (500 MHz, DMSO-d_6).

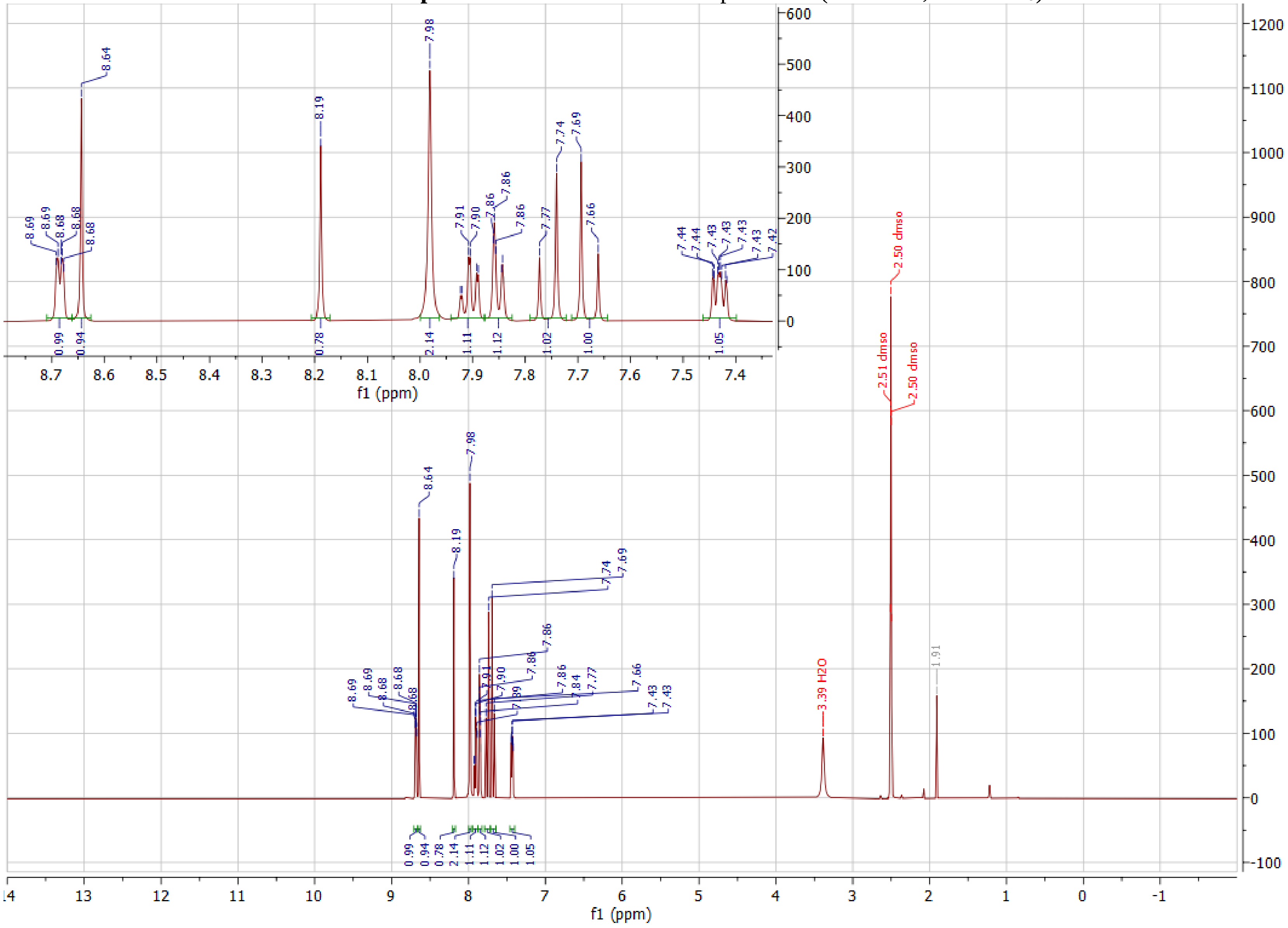

Spectrum11. ^{13}C-NMR of compound **27** (125 MHz, DMSO- d_6).

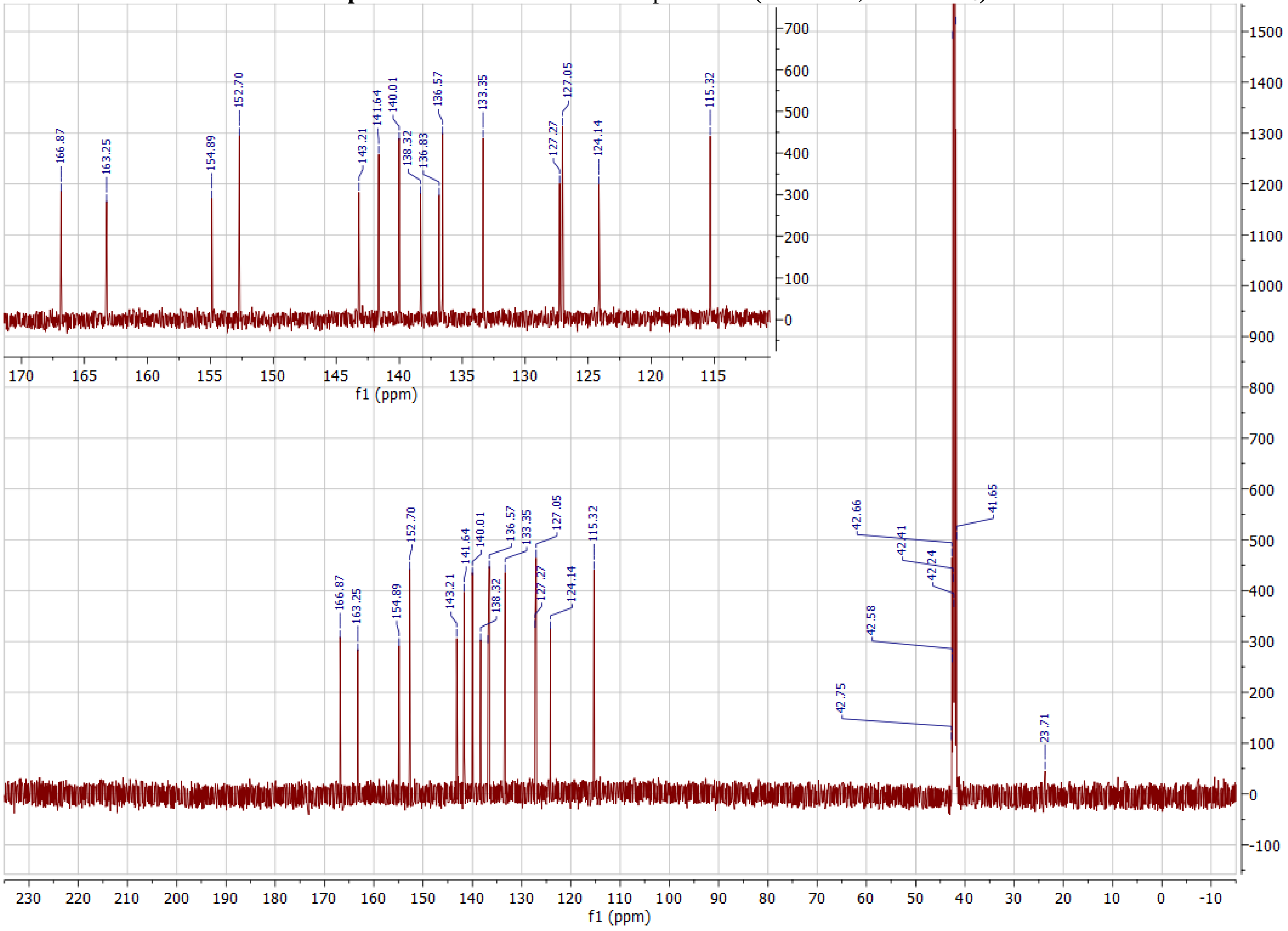

Spectrum 12. ^{1}H-NMR of compound **28** (500 MHz, DMSO-d_6).

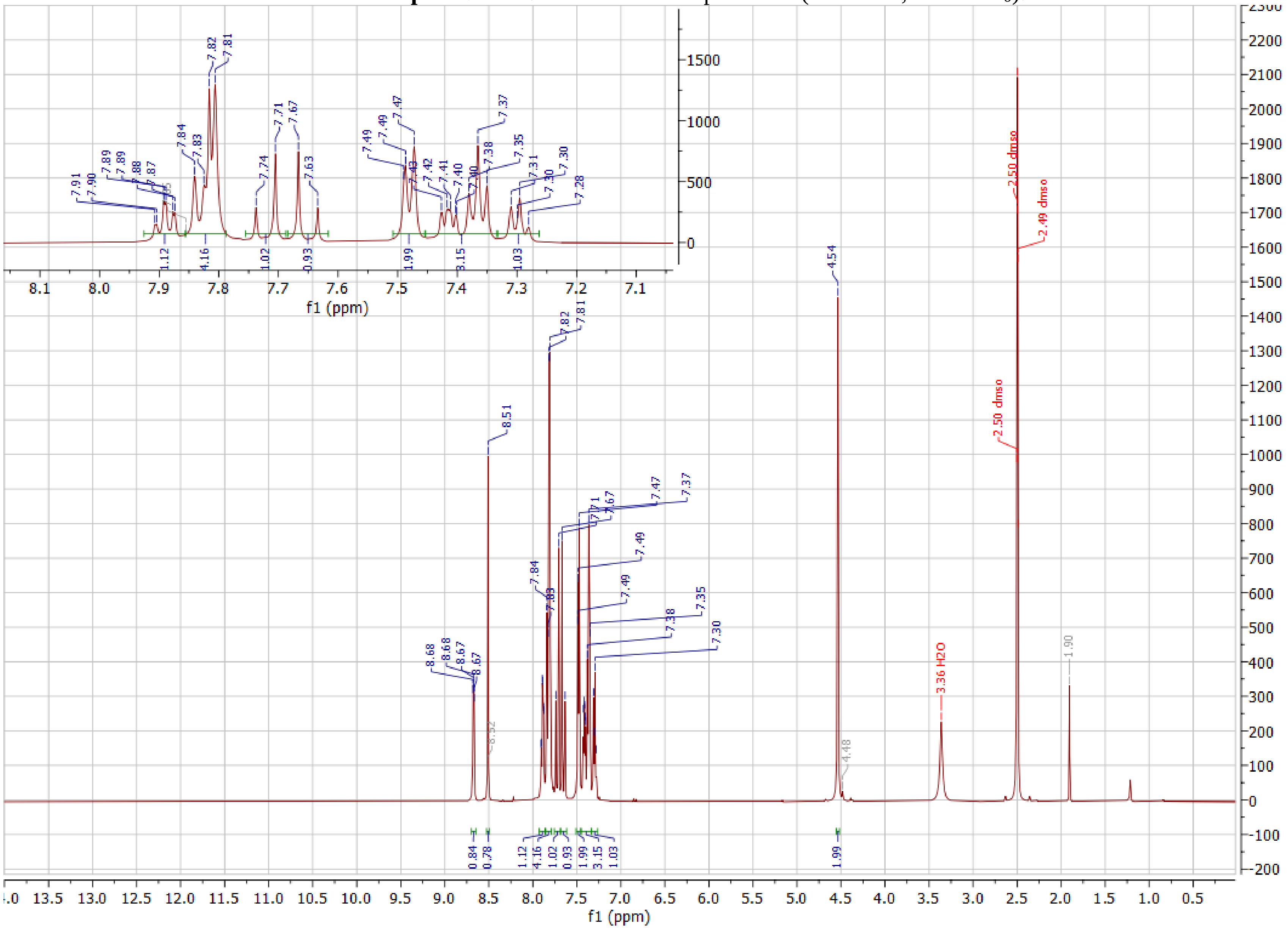

Spectrum13. ^{13}C-NMR of compound **28** (125 MHz, DMSO- d_6).

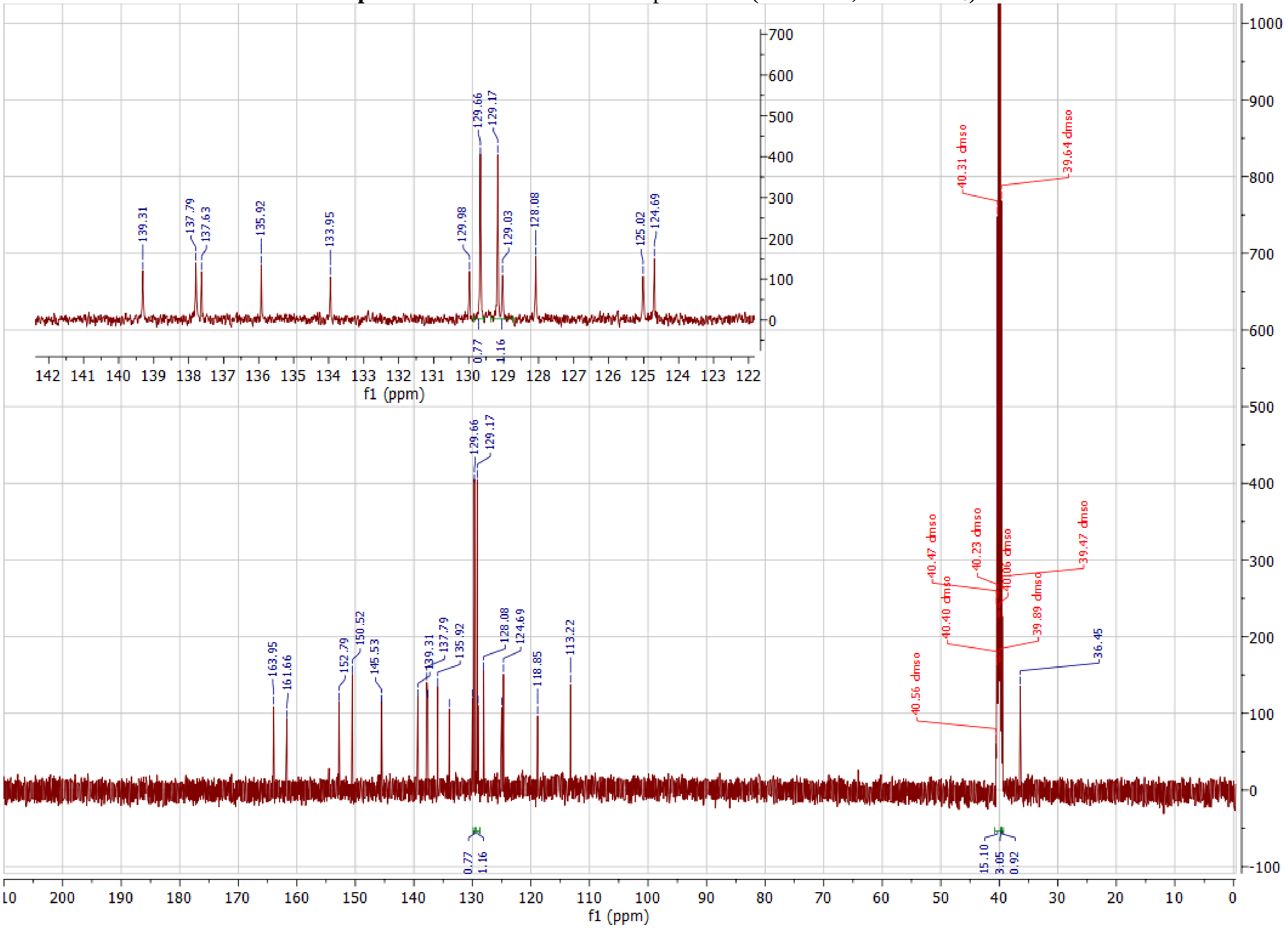

Spectrum 14. ^{1}H-NMR of compound **31** (500 MHz, DMSO-d_6).

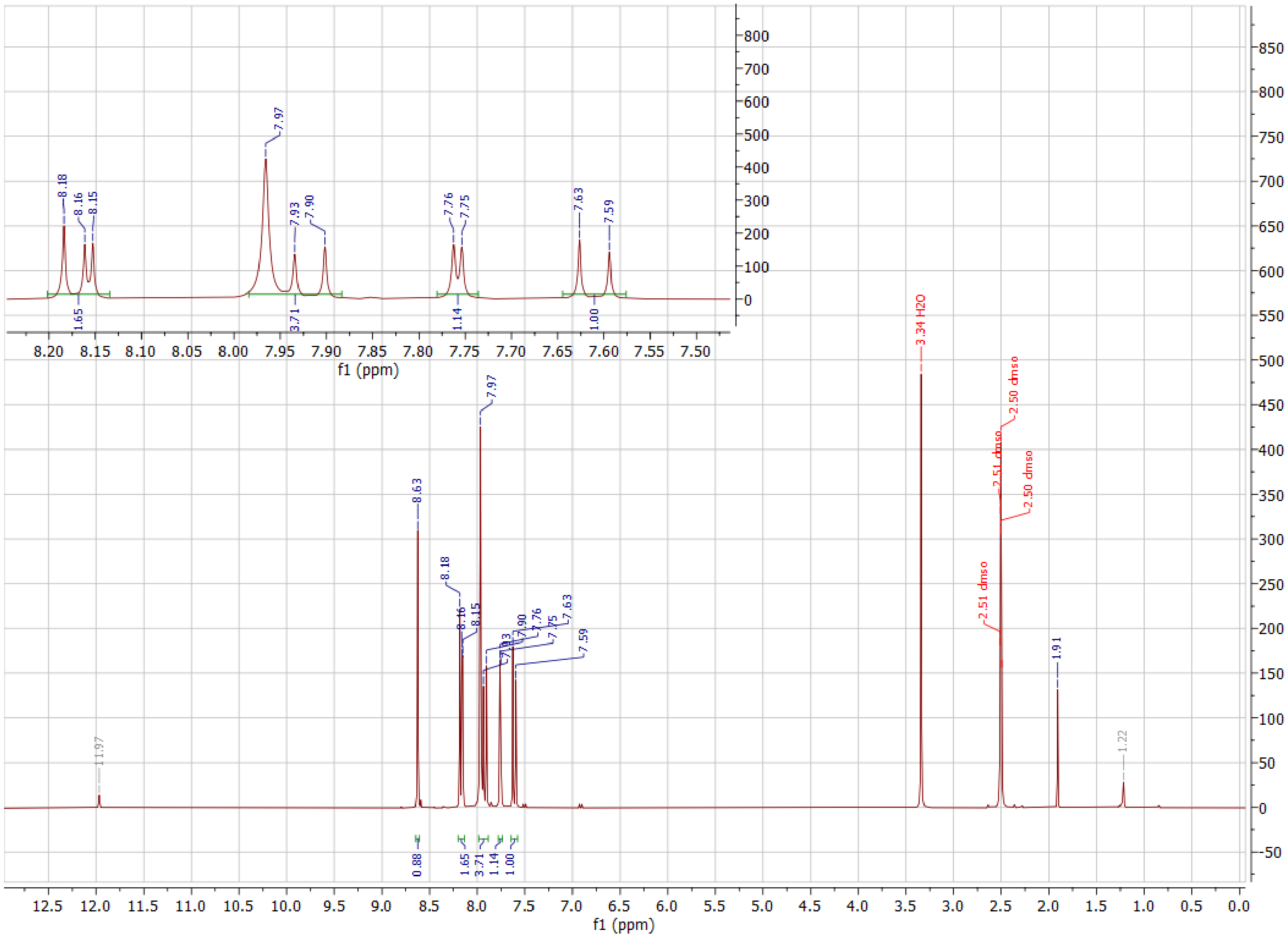

Spectrum15. ^{1}H-NMR of compound **32** (500 MHz, DMSO- d_6).

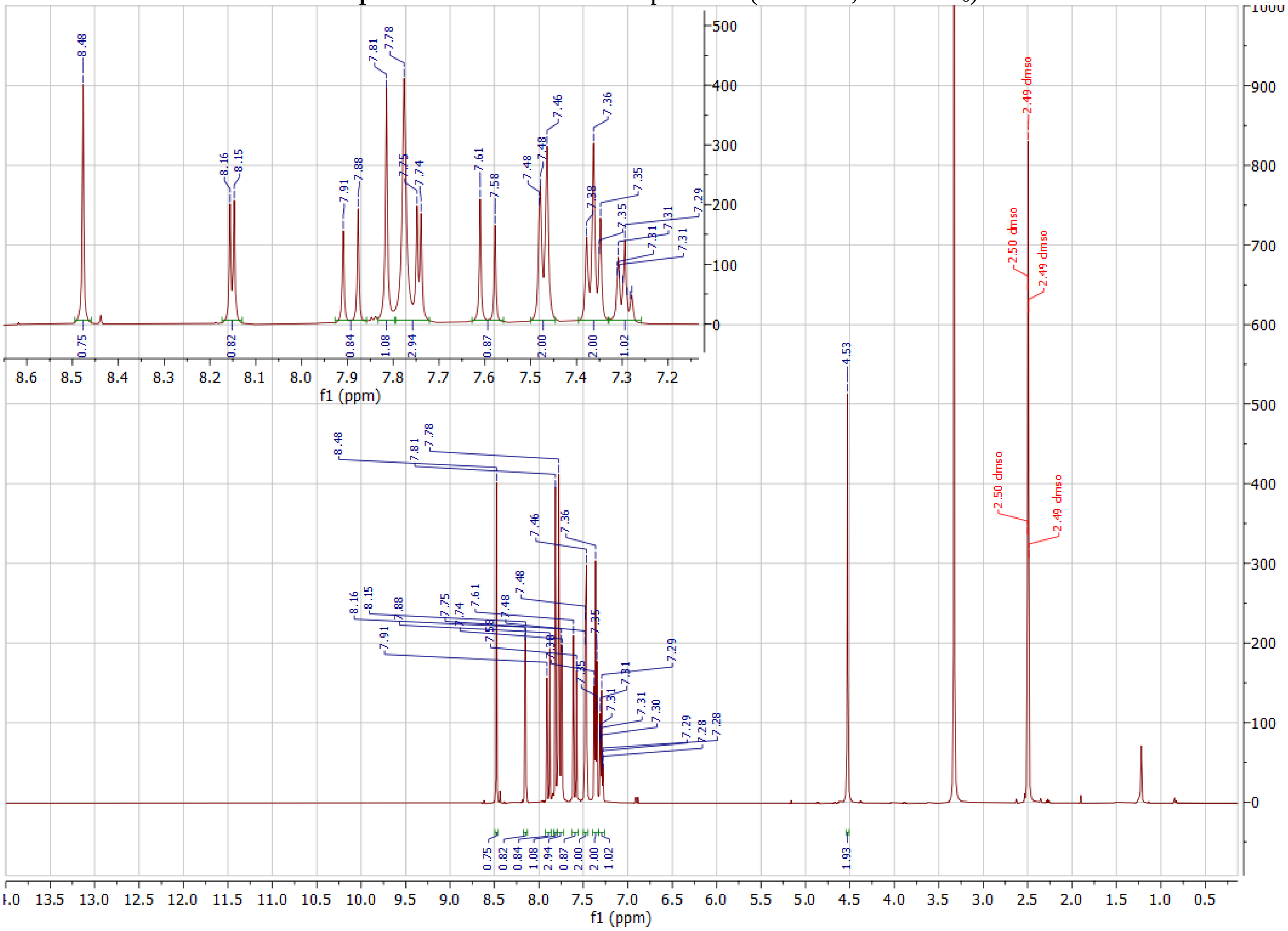

Spectrum 16. ^{1}H-NMR of compound **35** (500 MHz, DMSO-d_6).

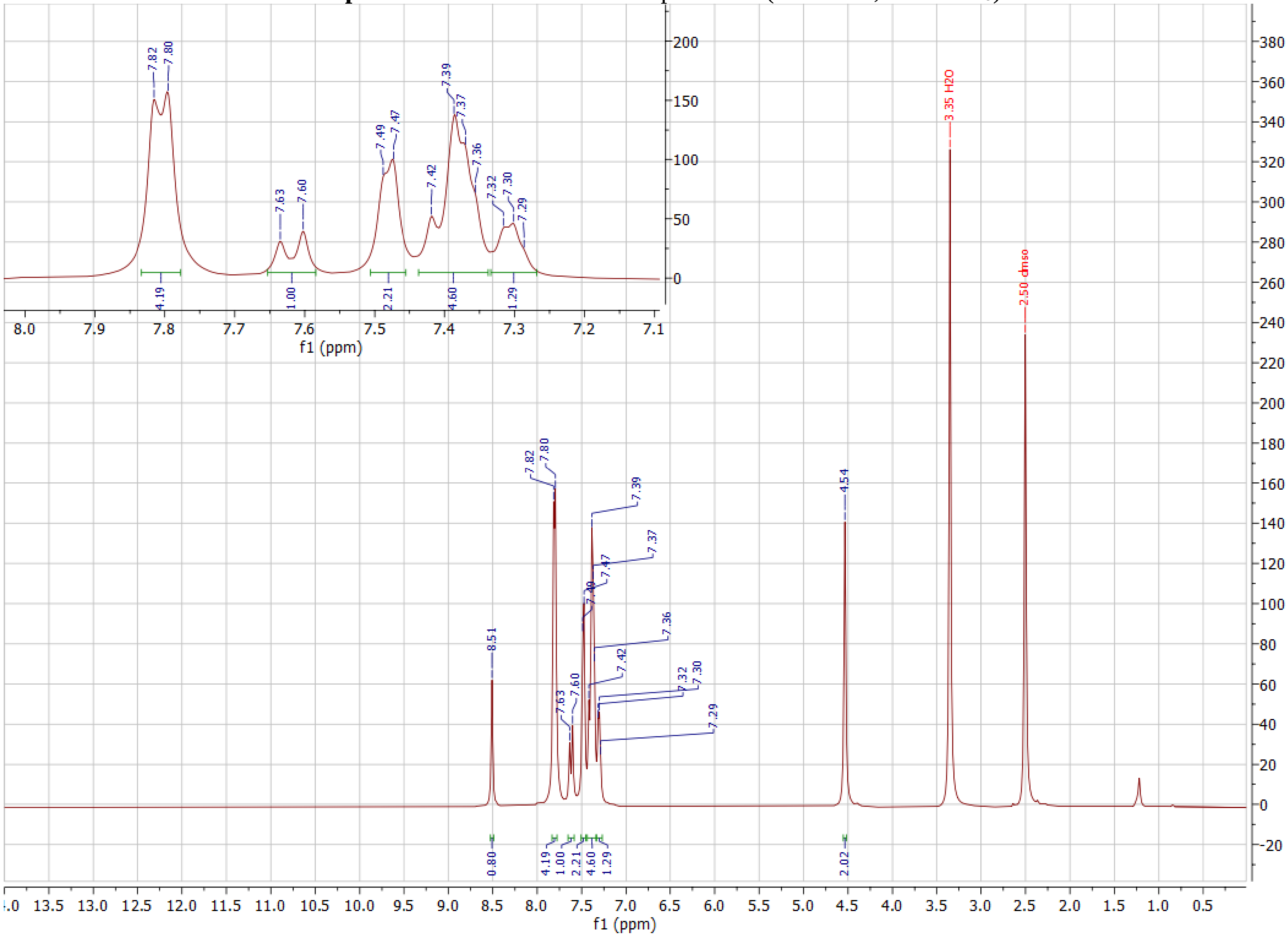

Spectrum17. ^{13}C-NMR of compound **35** (125 MHz, DMSO- d_6).

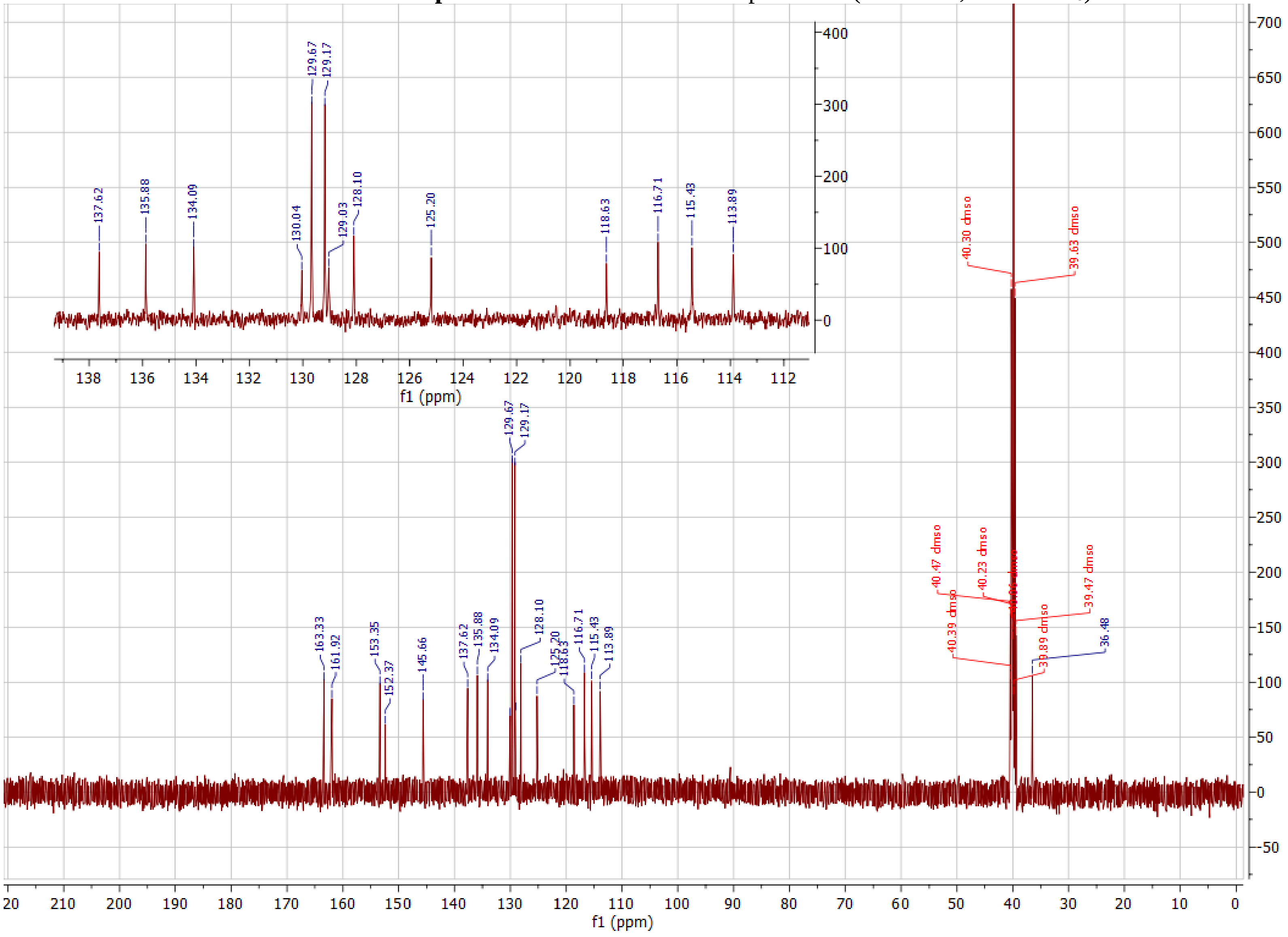

Spectrum 18. ^{1}H-NMR of compound **36** (500 MHz, DMSO-d_6).

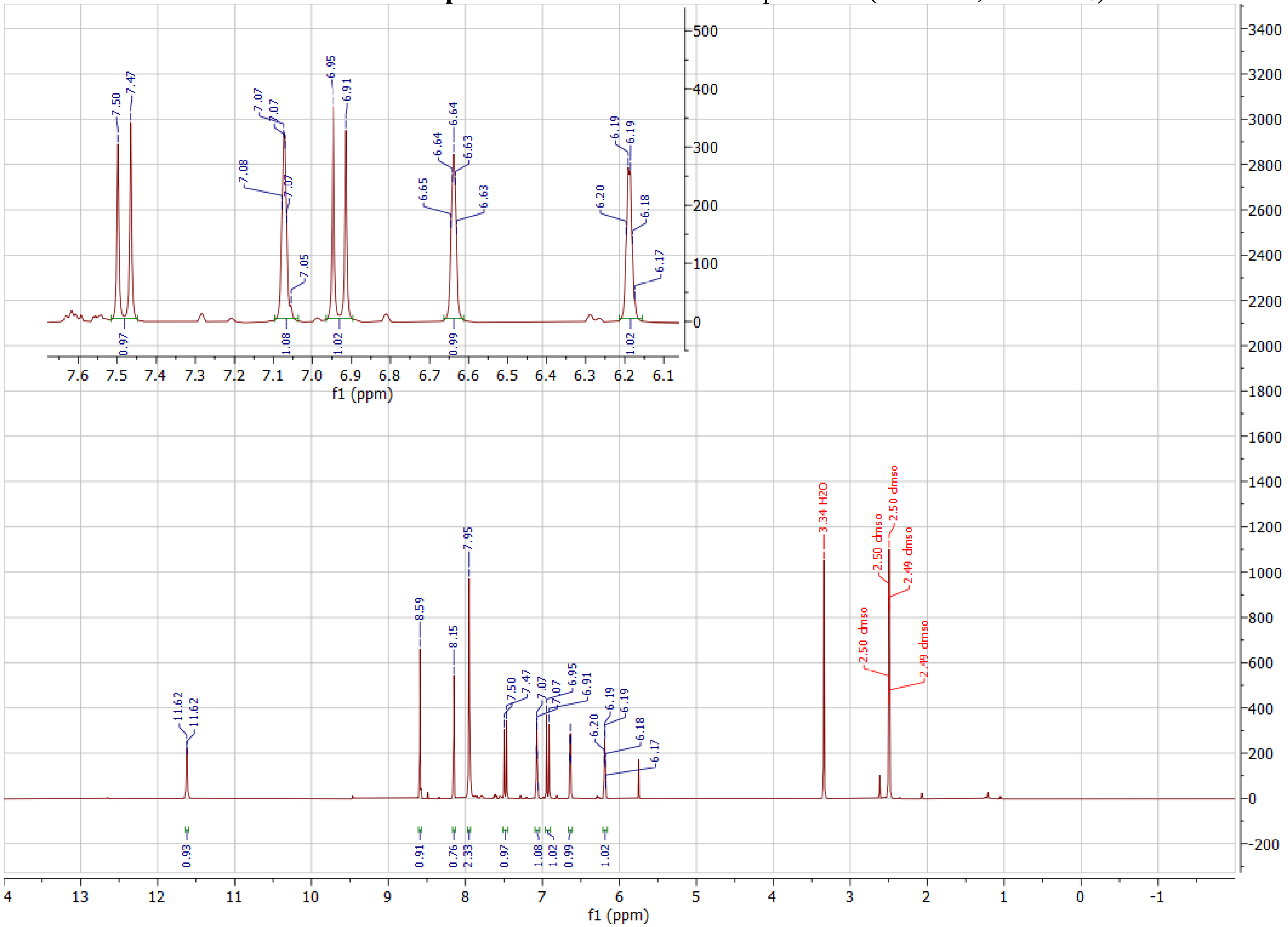

Spectrum 19. ^{13}C-NMR of compound **36** (125 MHz, DMSO- d_6).

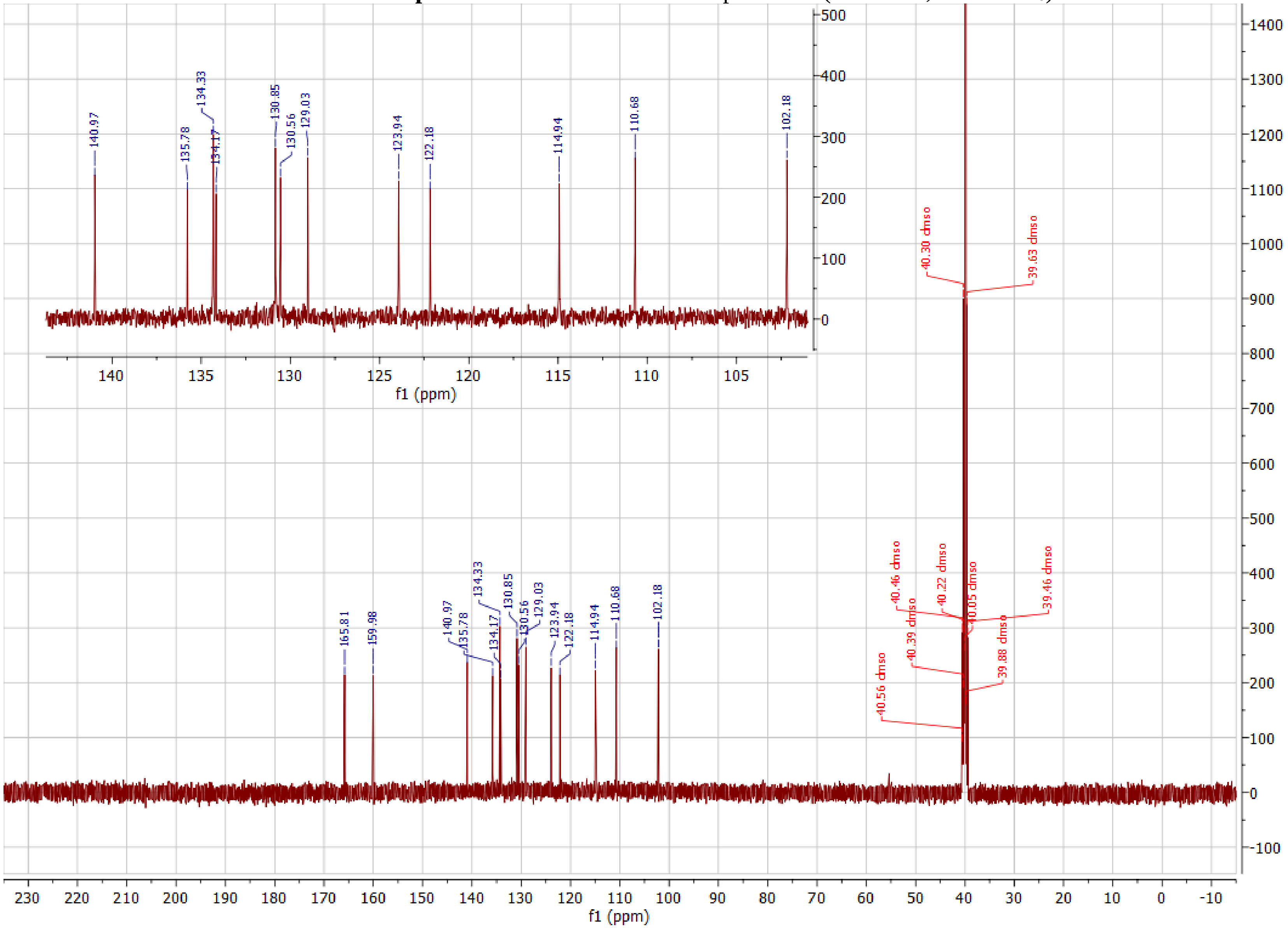

Spectrum 20. ^{1}H-NMR of compound **43** (500 MHz, DMSO-d_6).

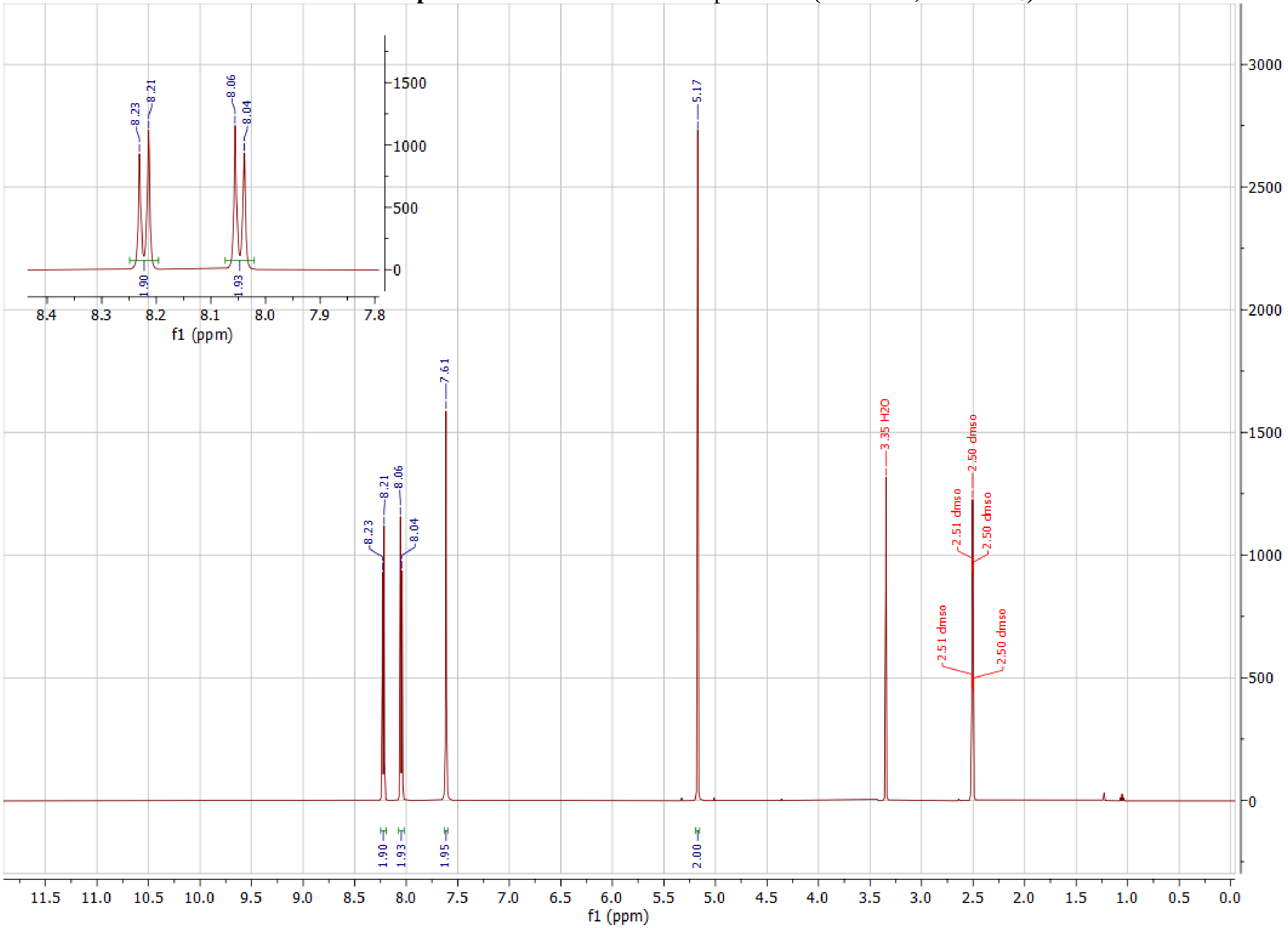

Spectrum 21. ^{13}C-NMR of compound **43** (125 MHz, DMSO- d_6).

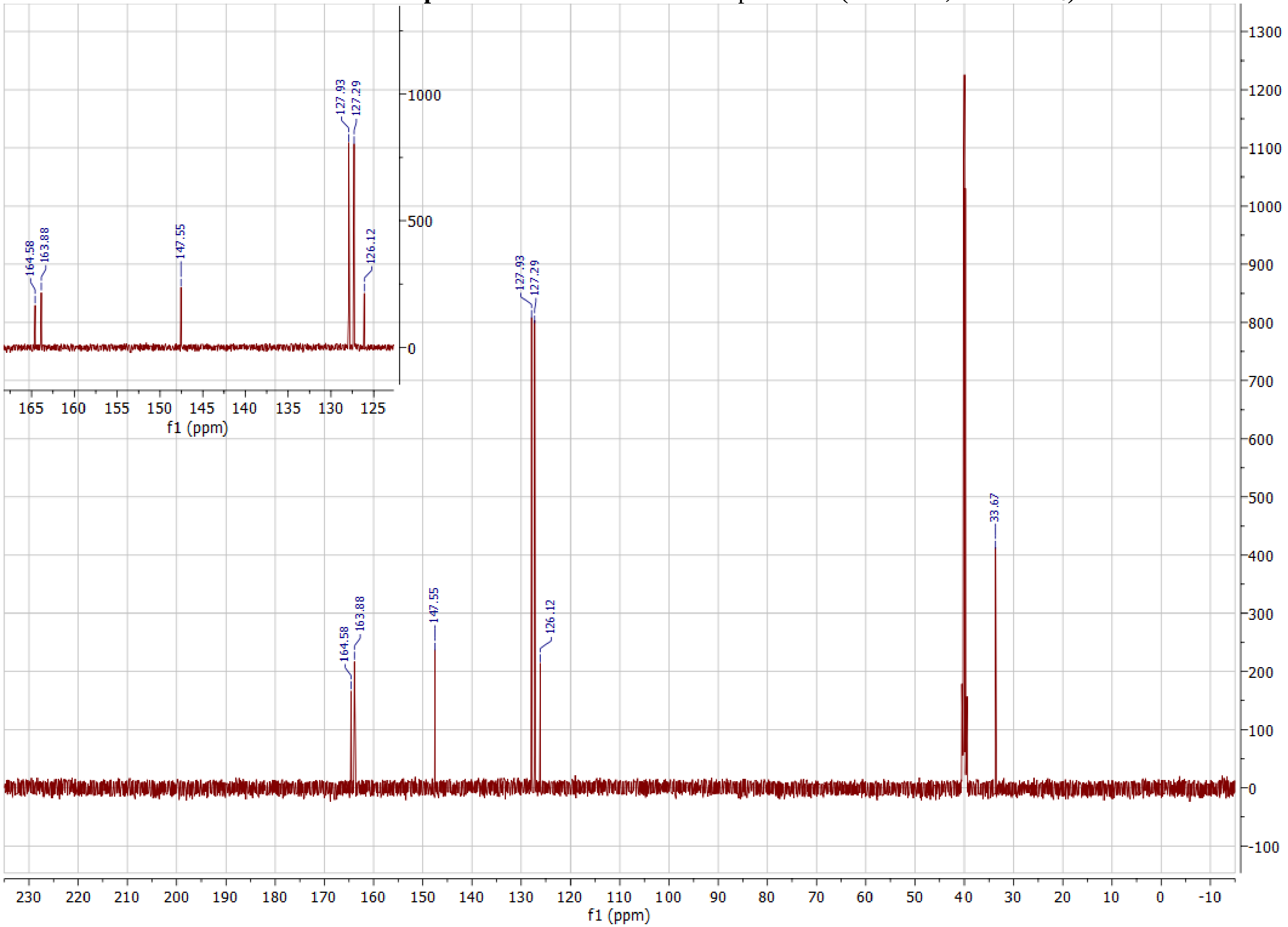

Spectrum 22. ^{1}H-NMR of compound **47** (500 MHz, DMSO-d_6).

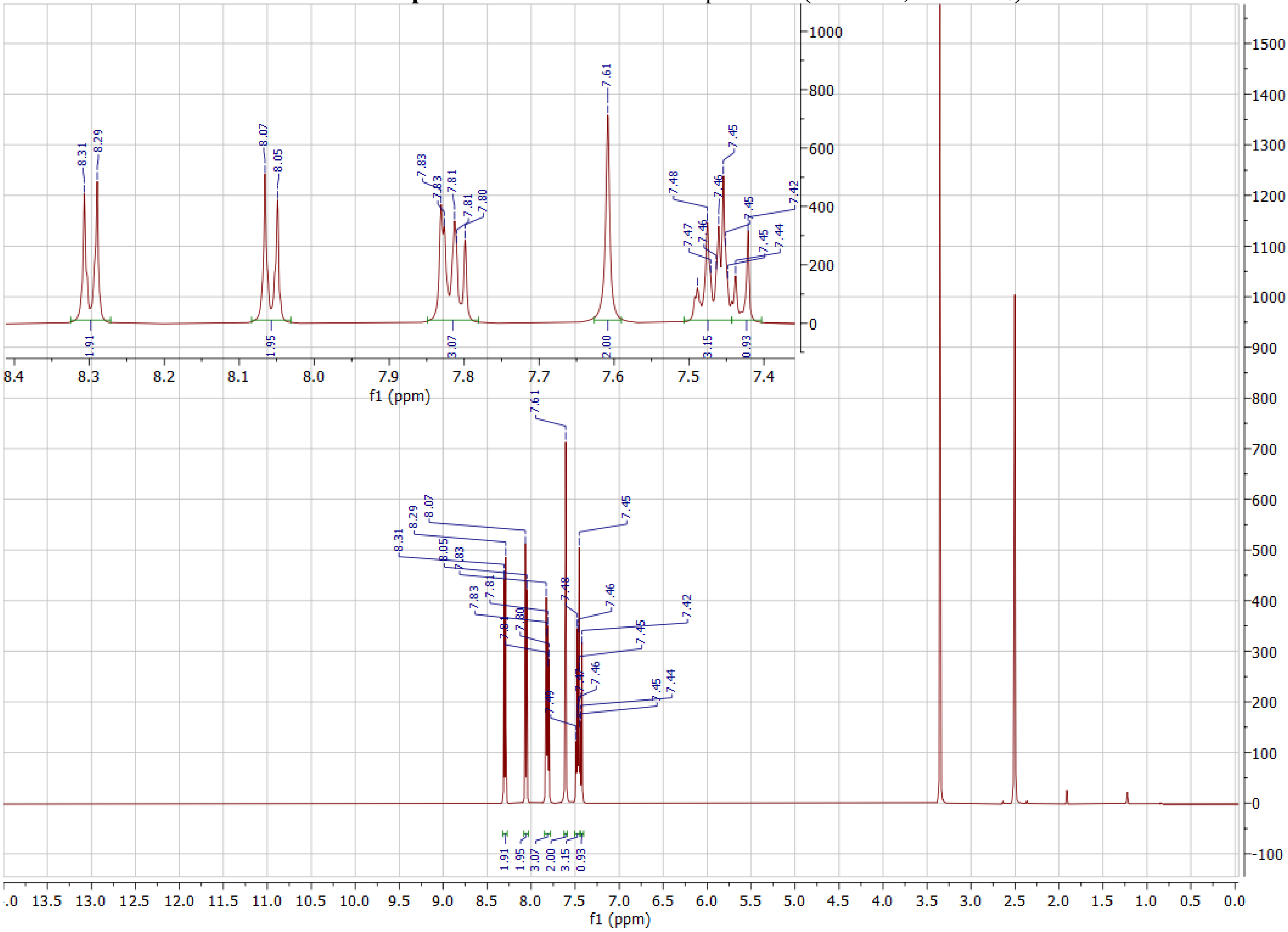

Spectrum 23. ^{13}C-NMR of compound **47** (125 MHz, DMSO- d_6).

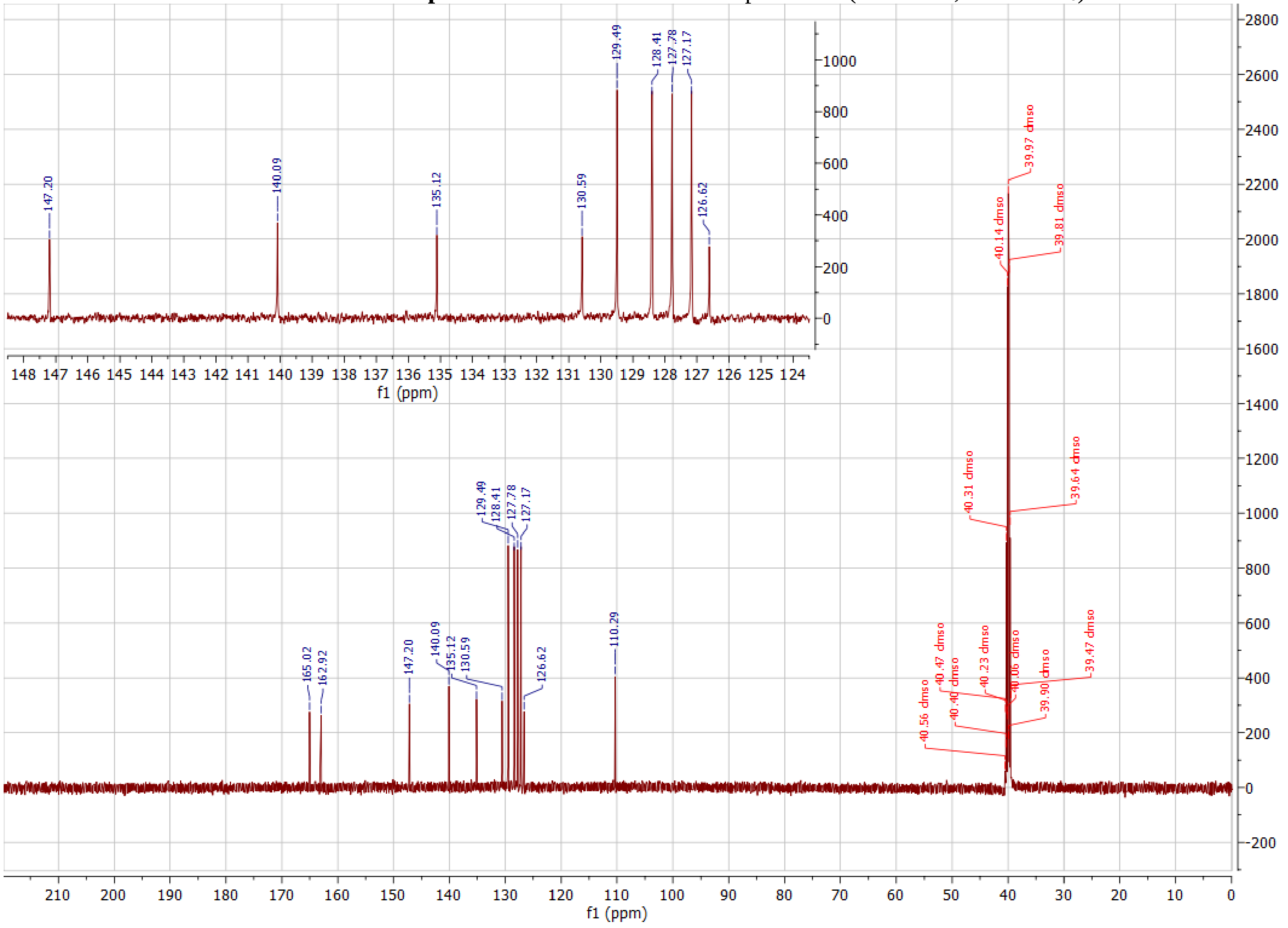

Spectrum 24. ^{1}H-NMR of compound **50** (500 MHz, DMSO-d_6).

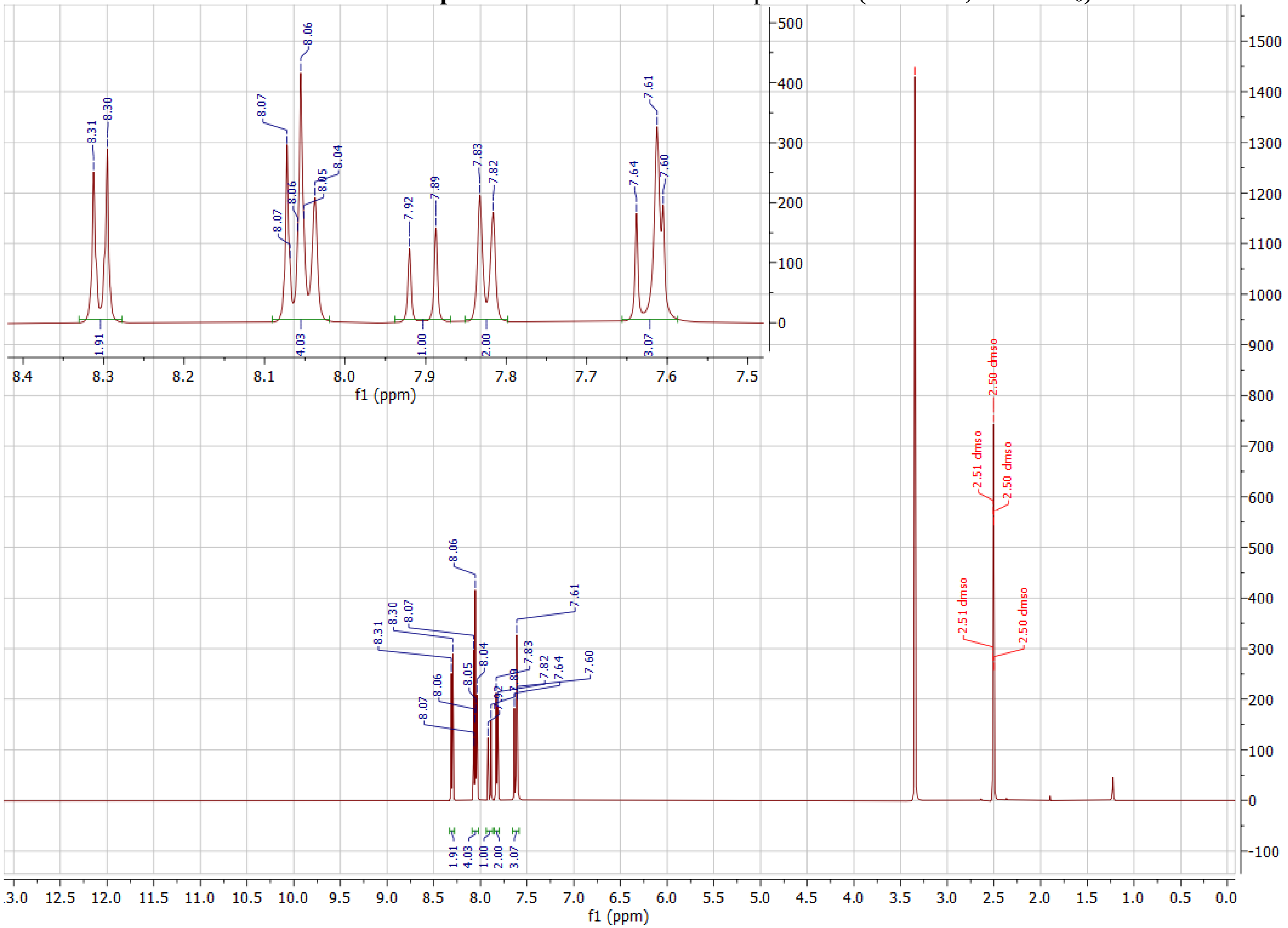

Spectrum 25. ^{13}C-NMR of compound **50** (125 MHz, DMSO- d_6).

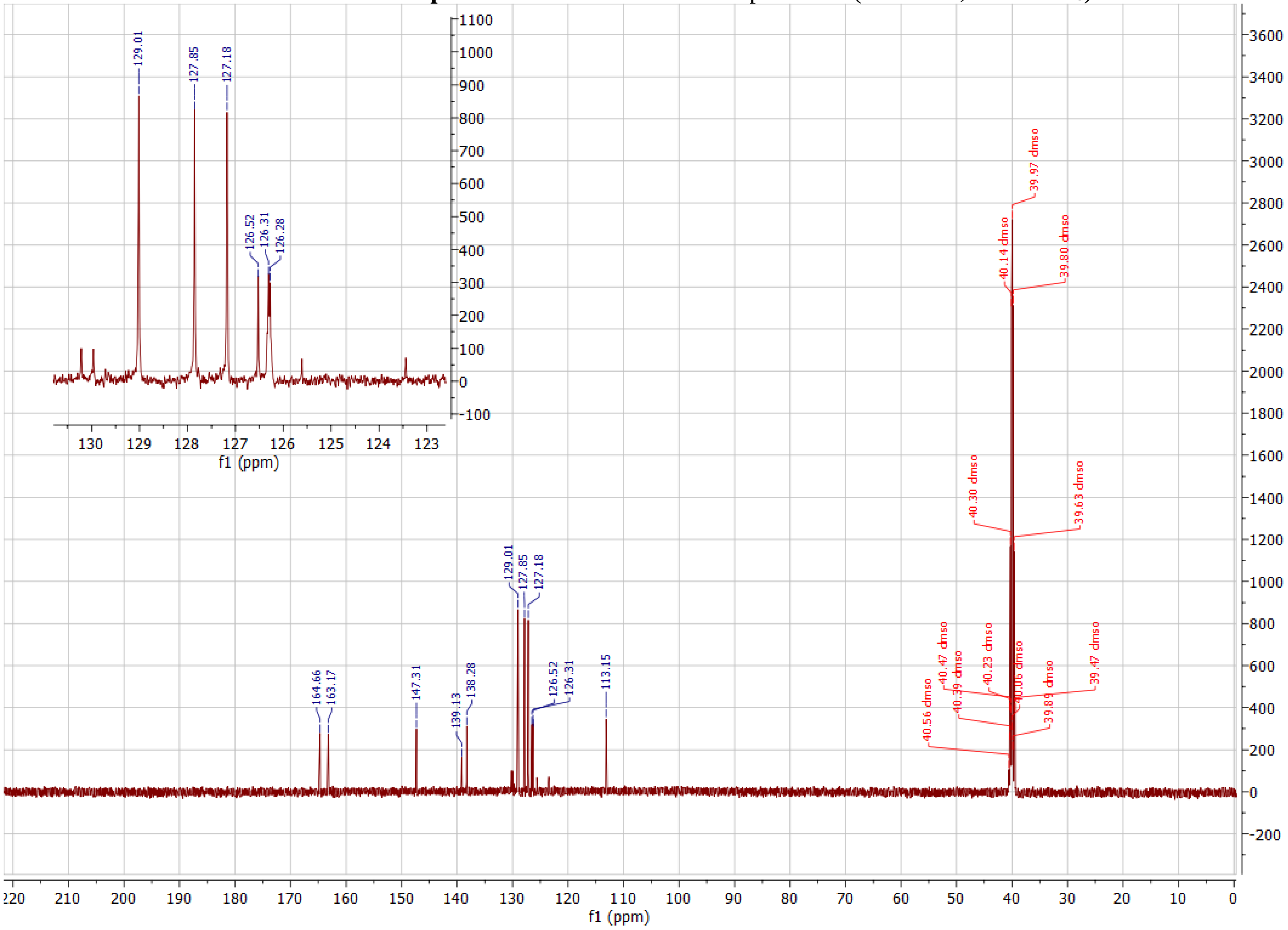

Spectrum 26. ^{1}H-NMR of compound **51** (500 MHz, $CDCl_3$).

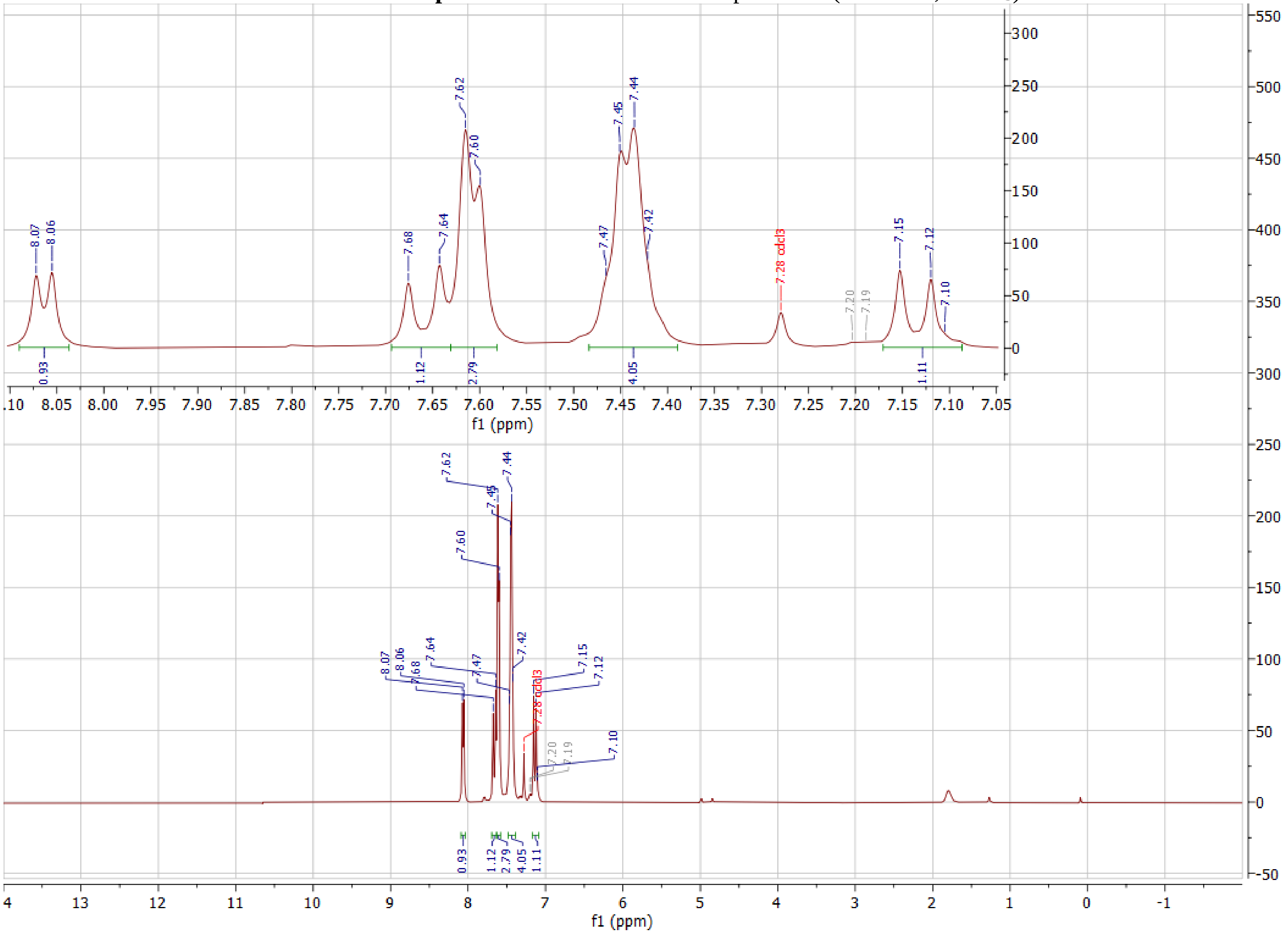

Spectrum 27. ^{13}C-NMR of compound **51** (125 MHz, $CDCl_3$).

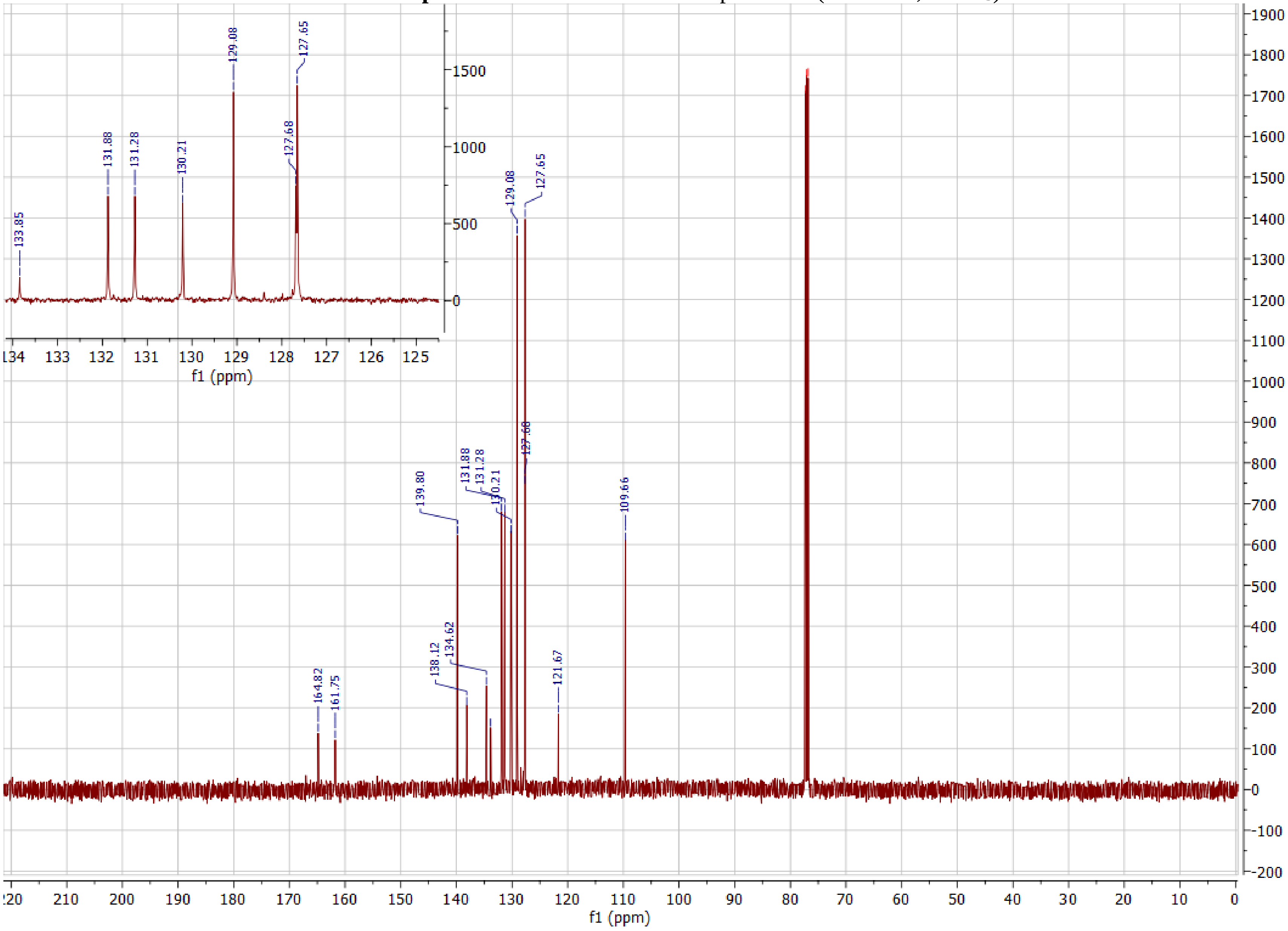

Spectrum 28. ^{1}H-NMR of compound **54** (500 MHz, DMSO-d_6).

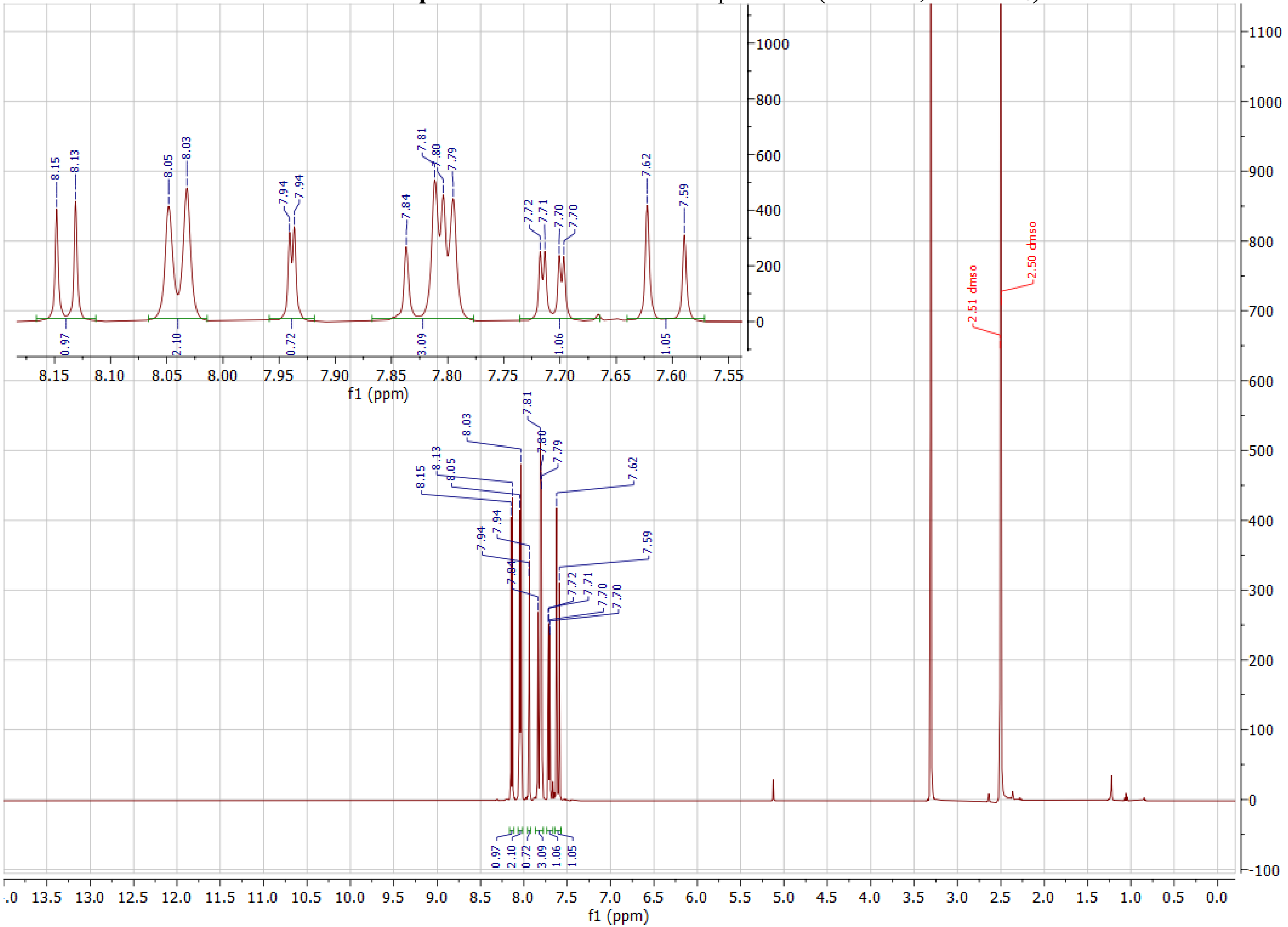

Spectrum 29. ^{13}C-NMR of compound **54** (125 MHz, DMSO- d_6).

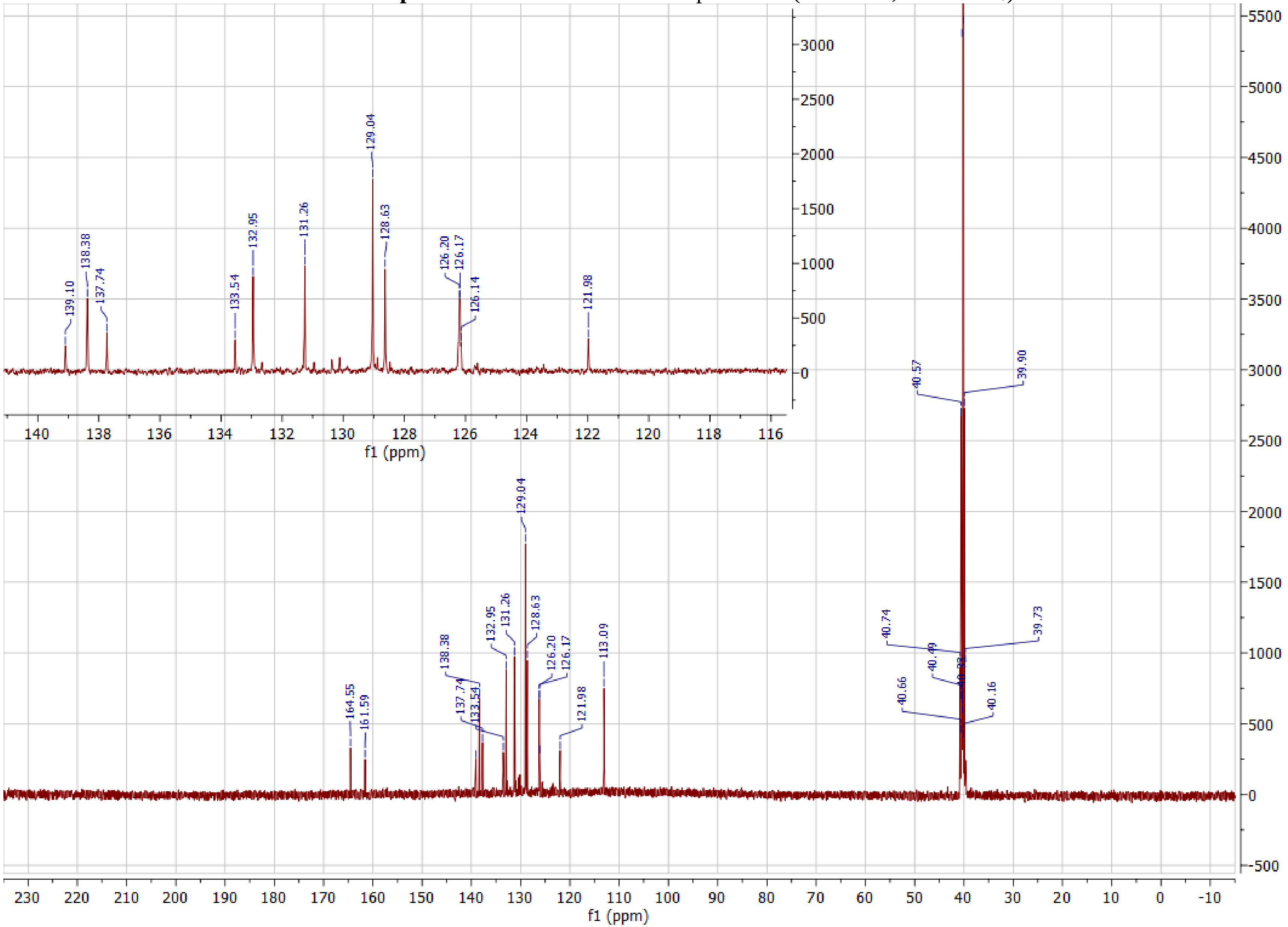

Supplement: Supplementary file 1 [file ijms-21-02235-s001.pdf]
